# Modifications to residential neighbourhood characteristics and risk of 79 common health conditions: a prospective cohort study

**DOI:** 10.1016/S2468-2667(21)00066-9

**Published:** 2021-05-26

**Authors:** Mika Kivimäki, G David Batty, Jaana Pentti, Solja T Nyberg, Joni V Lindbohm, Jenni Ervasti, Carlos Gonzales-Inca, Sakari B Suominen, Sari Stenholm, Pyry N Sipilä, Payam Dadvand, Jussi Vahtera

**Affiliations:** a Department of Epidemiology and Public Health, University College London, London, UK; b Clinicum, Faculty of Medicine, University of Helsinki, Helsinki, Finland; c Finnish Institute of Occupational Health, Helsinki, Finland; d Department of Public Health, University of Turku, Turku, Finland; e Department of Geography and Geology, Turku, Finland; f Centre for Population Health Research, University of Turku and Turku University Hospital, Turku, Finland; g University of Skövde, Skövde, Sweden; h Barcelona Institute for Global Health, Barcelona, Spain; i Universitat Pompeu Fabra, Barcelona, Spain; j CIBER Epidemiología y Salud Pública, Madrid, Spain

## Abstract

**Background:**

Observational studies have identified a link between unfavourable neighbourhood characteristics and increased risk of morbidity, but it is unclear whether changes in neighbourhoods affect future disease risk. We used a data-driven approach to assess the impact of neighbourhood modification on 79 health outcomes.

**Methods:**

In this prospective cohort study, we used pooled, individual-level data from two Finnish cohort studies: the Health and Social Support study and the Finnish Public Sector study. Neighbourhood characteristics (mean educational level, median income, and employment rate of residents, and neighbourhood green space) and individual lifestyle factors of community-dwelling individuals were assessed at baseline (at different waves starting between 1998 and 2013). We repeated assessment of neighbourhood characteristics and lifestyle factors approximately 5 years from each baseline assessment, after which follow-up began for health conditions diagnosed according to the WHO International Classification of Diseases for 79 common health conditions using linkage to electronic health records. We used Cox proportional hazard regression models to compute adjusted hazard ratios (HRs) of incident disease associated with neighbourhood characteristics and changes in neighbourhood characteristics over time and logistic regression analysis to compute adjusted odds of association between changes in neighbourhood characteristics and individual lifestyle factors.

**Findings:**

114 786 individuals (87 012 [75·8%] women; mean age 44·4 years [SD 11·1]) had complete data and were included in this cohort study. During 1·17 million person-years at risk, we recorded 164 368 new-onset health conditions and 3438 deaths. Favourable changes in neighbourhood characteristics were associated with reduced risk of all-cause mortality and incidence of 19 specific health conditions. Unfavourable changes were correspondingly associated with increased risk of mortality and 27 specific health conditions. Among participants who did not move residence during the observation period, relative to individuals who continually lived in disadvantaged neighbourhoods, those who experienced favourable modifications in neighbourhood characteristics had a lower risk of future diabetes (HR 0·84, 95% CI 0·75–0·93), stroke (0·49, 0·29–0·83), skin disease (0·72, 0·53–0·97), and osteoarthritis (0·87, 0·77–0·99). Living in a neighbourhood with improving characteristics was also associated with improvements in individual-level health-related lifestyle factors. Among participants who lived in advantaged residential environments at baseline, unfavourable changes in neighbourhood characteristics were associated with an increased risk of diabetes, stroke, skin disease, and osteoarthritis compared with individuals who lived in advantaged neighbourhoods throughout the study period.

**Interpretation:**

Favourable modifications to residential neighbourhoods showed robust, longitudinal associations with a range of improvements in health outcomes, including improved health behaviours and reduced risk of cardiometabolic, infectious, and orthopaedic conditions.

**Funding:**

UK Medical Research Council, US National Institute on Aging, NordForsk, and Academy of Finland.

## Introduction

Residential neighbourhoods might influence human health by affecting social cohesion and access to health care, healthy foods, and education, availability of recreational facilities, and environmental exposures. Findings from observational studies suggest that unfavourable neighbourhood characteristics can have a number of health effects, including an association between neighbourhood socioeconomic disadvantage and increased risk of diabetes, cardiovascular disease, and behavioural disorders;^1^, ^2^, ^3^, ^4^, ^5^ and an association between outdoor pollution and an excess of cardiovascular and cerebrovascular diseases and respiratory conditions such as asthma.^6^, ^7^ An association between absence of residential green space and risk of metabolic syndrome, poor mental health, and premature mortality has also been reported.^8^, ^9^, ^10^, ^11^, ^12^ Furthermore, various other characteristics of built environments (eg, access to public transport, walkability, and location of employment and services) might also be associated with human health.^13^, ^14^, ^15^

**Research in context**

**Evidence before this study**

It is increasingly recognised that residential neighbourhoods have an impact on human health and wellbeing. We searched PubMed from database inception to Nov 10, 2020, for publications on the effects of neighbourhood characteristics on health, without language or date restrictions, using the search terms “neighbourhood” (title word) in combination with “morbidity”, “mortality”, “disease”, “disorder”, and “injury”. Our search yielded more than 3000 publications, which included meta-analyses of studies on obesity, infectious diseases, respiratory diseases, psychiatric disorders, and mortality. Although some studies included a range of health endpoints, we found no outcome-wide studies on mental and physical diseases that covered the full array of bodily systems. Few longitudinal studies assessed whether favourable and unfavourable changes in neighbourhood characteristics were associated with corresponding changes in health.

**Added value of this study**

To facilitate a comprehensive assessment of morbidity and mortality associated with residential neighbourhood characteristics, we did an outcome-wide study based on two large prospective cohorts, comprising 114 786 adults. In conventional epidemiological analyses accounting for multiple comparisons, one or more of employment, education, income, and green space were associated with 30 health conditions, including mortality. Favourable changes in neighbourhood characteristics were associated with death and 19 other health outcomes and unfavourable changes were associated with death and 27 health outcomes. By comparing the health of people who stayed living in the same neighbourhood throughout the study, but whose neighbourhood's characteristics changed over time, with those residing in areas with stable conditions, we aimed to investigate the effect of favourable or unfavourable modifications to neighbourhoods on health. Compared with those continually living in disadvantaged neighbourhoods, participants living in neighbourhoods with favourable modification had a lower risk of future diabetes, stroke, skin disease, and osteoarthritis. Among participants who originally lived in advantaged residential environments, a deterioration of neighbourhood characteristics was associated with an increased risk of diabetes, stroke, skin disease, and osteoarthritis compared with those who continually lived in advantaged neighbourhoods. Improving or deteriorating neighbourhood conditions were also associated with favourable or unfavourable changes in selected lifestyle factors, including smoking, alcohol consumption, physical activity, and body-mass index, which supports the plausibility of associations between changing neighbourhood characteristics and health outcomes. Additionally, linking neighbourhood characteristics to health outcomes among residents enabled the generation of a health atlas for neighbourhood research, which includes associations between four neighbourhood characteristics (education, income, unemployment, and green space) and 79 outcomes and associations between changes in the four neighbourhood characteristics and these health outcomes.

**Implications of all the available evidence**

The findings of this data-driven study add to existing evidence suggesting that residential neighbourhoods might have wide-ranging effects on human health. Our observation that improvements in residential neighbourhoods were associated with positive lifestyle changes and reduced risk of incident cardiometabolic, infectious, and orthopaedic diseases supports the notion that policies addressing neighbourhood disadvantage might be beneficial in terms of improving public health. Additionally, the data atlas generated, which includes all prospective associations of four major neighbourhood characteristics with 79 morbidity and mortality outcomes from conventional epidemiological analyses, provides a large reference database for future studies on the health effects of residential neighbourhoods.

However, there are important limitations to such research. Comprehensive evaluations of health outcomes associated with neighbourhood characteristics are scarce. Most studies to date have had a narrow research focus on specific health outcomes, which hampers insights into specificity of health effects. Outcome-wide approaches covering a wide range of diseases simultaneously have several advantages. Such approaches are less susceptible to investigator bias; more likely to recognise and report null findings; and provide a platform for comparing effect sizes across different health conditions.^16^ To the best of our knowledge, no outcome-wide studies have assessed the association between residential neighbourhood characteristics and health.

Another limitation of previous research is that evidence is often obtained from comparisons of disease incidence between different residential neighbourhoods and might therefore be confounded by health-related self-selection into certain residential environments. This bias occurs when peoples’ health influences their choices to move to a particular area, which artificially inflates observed associations. Natural experiments focused on health changes in people who do not move residence but experience modifications in neighbourhood characteristics minimise health-related selection bias.

In this prospective study of neighbourhood characteristics and health, we used an outcome-wide approach^16^ to simultaneously assess 79 common health outcomes among individuals in advantaged and disadvantaged neighbourhoods.

## Methods

### Study population

In this prospective cohort study, we used individual participant data from two Finnish prospective cohort studies: the Health and Social Support (HeSSup) study^17^ and the Finnish Public Sector (FPS) study.^18^ Ethical approval for both studies was obtained from local ethics committees. Written informed consent was obtained from all participants.

The selection of the analytical samples and data collection phases for each study are described in appendix 1 (pp 3–5). The HeSSup study included a stratified random sample of the Finnish population based on four age groups (20–24, 30–34, 40–44, and 50–54 years); the FPS sample comprised the entire public sector personnel of ten cities and 21 hospitals in the same geographical areas. In the HeSSup study, 23 655 adults who responded to the baseline survey between June 1, 1998, and May 31, 2000, were sent a follow-up survey between Jan 1 and Aug 31, 2003, had data on residential neighbourhoods, and were successfully linked to electronic health records until Dec 31, 2012. The FPS occupational cohort comprised 91 131 adults who responded to at least one of four surveys done between March 1, 2000, and Nov 15, 2013, had data on residential neighbourhoods and were linked to health records until Dec 31, 2018. Geocoded maps of participants’ residential locations are in appendix 1 (p 5).

### Neighbourhood characteristics and covariates

To assess change in neighbourhood characteristics from baseline, we measured residential neighbourhood characteristics using three annual indices of neighbourhood socioeconomic composition (education, income, and unemployment) and an index of green space in the area. Data for these indices were obtained at year 1 and year 5 (ie, exposure period), after which follow-up for morbidity and mortality started (appendix 1 p 5).

For each participant, we obtained geocoded residential addresses and dates of changes in residence from the Population Register Centre of Finland; these data were positioned to the Statistics Finland Grid Database. We obtained data on neighbourhood characteristics from the Statistics Finland Grid Database, which assigns these indices (education, income, and employment status) to all Finnish residents in 250 m × 250 m grids. Our main analysis was based on these grid dimensions (32 904 neighbourhood locations) since previous studies have shown stronger neighbourhood-health relationship at this scale relative to larger spatial units.^19^ For comparison, we used 750 m × 750 m grids in which the participants’ address was in the middle grid for sensitivity analyses.

The socioeconomic composition of each grid comprised the mean number of years of education of residents aged older than 18 years, the median annual income of households, and the proportion of unemployed adult residents in each year. Green space was defined as any open land surface that was partly or completely covered with grass, trees, shrubs, or other vegetation (eg, parks, forests, and community gardens). To assess the degree of residential surrounding green space, we calculated the mean normalised difference vegetation index (NDVI) for each grid from a satellite image composite using Google Earth Engine (appendix 1 p 5). Since the summer months are the greenest months in Finland, we obtained NDVI maps for June, July, and August to maximise the contrast in estimated exposure.

Covariates for the morbidity and mortality analyses were measured at year 4 or 5 of the study period—ie, between 13 months and up to 1 day before the start of follow-up for morbidity and mortality. Covariates included participant age, sex, education (primary, secondary, or tertiary), marital status (married or cohabiting *vs* single), employment during the 5-year period before the start of follow-up (4·5–5·0 years *vs* <4·5 years), population density in the neighbourhood (continuous index), and place of residence (urban area *vs* rural area). In FPS, data also included type of residence (one family, terraced, apartment), total number of rooms in the residence (1–3 *vs* >3 [dichotomous individual-level measures**]**), and floor area of residence (<87 m^2^ *vs* ≥87 m^2^ [based on median]).

### Morbidity and mortality outcomes

Participants were linked by their unique national identification number to national registries of hospital discharge information (recorded by the National Institute for Health and Welfare) and mortality (recorded by Statistics Finland). Follow-up for morbidity and mortality started after the fifth year of exposure measurement (ie, after the point at which we had measured whether there was a change in neighbourhood characteristics) and continued until disease onset, death, or end of follow-up. The electronic health records included cause (primary diagnosis) and date of hospital discharge or mortality. Additional information for specific health conditions was available via record linkage to the Drug Reimbursement Register of the Social Insurance Institution of Finland. The diagnosis for incident disease was coded according to the WHO International Classification of Diseases, Tenth Revision (ICD-10; appendix 1 pp 6–7).

### Lifestyle factors

We assessed the following individual-level lifestyle factors at baseline and again during the fourth or fifth year after baseline using identical standard survey instruments in both cohorts:^20^ smoking status (current, ex-smoker, or never smoker); alcohol consumption (heavy [>14 units of ethanol for women, >21 units of ethanol for men per week], moderate [1–14 units for women, 1–21 units for men per week], or non-drinker); physical activity based on metabolic equivalent of task-hours (low [<14 h per week], moderate [≥14 to <30 h per week], or high [≥30 h per week]); body-mass index (BMI; obese [BMI >30 kg/m^2^], overweight [BMI >25 to <30 kg/m^2^], or normal weight [BMI <25 kg/m^2^]); and a 5% or more change in bodyweight between years 1 and years 4 or 5 (appendix 1 pp 5–6).

### Statistical analysis

The five analytical steps included conventional epidemiological analyses (step 1, 2, and 5) and those emulating non-randomised neighbourhood modification trials (step 3 and 4; appendix 1 p 10). We pooled individual-level data from the two cohort studies. Linked records captured 1204 ICD codes, including 79 common diseases, health conditions, or death, used in this analysis (a complete list is included in appendix 1 [pp 6–7]).^18^

In step 1, having assessed the proportional hazards assumption (appendix 1 pp 8, 11–12), we examined the association of each neighbourhood characteristic with the incidence of 79 morbidity and mortality endpoints using Cox proportional hazards regression models. Each participant was followed up from the the end of the 5-year exposure period to the date of recorded disease onset, death, or end of follow-up, whichever occurred first. We categorised each neighbourhood characteristic as advantaged or disadvantaged using median values as the cutoff thresholds; higher income, higher educational level, lower unemployment rate, and larger green space were used to define an advantaged neighbourhood. We calculated hazard ratios (HRs) and their 95% CIs for living in an advantaged versus disadvantaged neighbourhood separately for each neighbourhood characteristic and health outcome pair. In the base model, HRs were adjusted for age, sex, education, and cohort. Bonferroni correction for 79 tests (the number of health outcomes for each neighbourhood characteristic) was used to adjust for multiple testing with a p value of less than 0·0006 considered to indicate statistical significance (appendix 1 p 7).

In steps 2–4, we restricted analyses to health outcomes that were significantly associated with one or more neighbourhood characteristics after Bonferroni correction. In the second step, we analysed separately associations of favourable change (ie, in-situ changes in location from disadvantaged to advantaged or moving from disadvantaged to advantaged neighbourhoods) and unfavourable change (ie, in-situ changes in location from advantaged to disadvantaged or moving from advantaged to disadvantaged neighbourhoods) with subsequent health outcomes using individuals with stable disadvantaged or stable advantaged neighbourhoods as the reference in Cox regression analysis.

Step 3 of the analyses emulated non-randomised trials of favourable and unfavourable neighbourhood modifications (appendix 1 pp 8, 33).^21^ To assess associations between improvement in each neighbourhood characteristic and health outcomes, we selected all participants living in a disadvantaged neighbourhood at baseline. For each neighbourhood characteristic and health outcome, we computed a Cox regression model in which the treatment group included participants whose neighbourhood classification changed from disadvantaged to advantaged. The reference group included participants who lived in a disadvantaged neighbourhood throughout the exposure period. To minimise bias due to health-related selection into residential environments, we focused this analysis on a subgroup of participants who did not move residence during the 5-year exposure period (ie, excluding any individual who moved address). Similarly, we fitted corresponding Cox regression models to assess the associations between unfavourable change in each neighbourhood characteristic and health outcomes in participants living in an advantaged neighbourhood at baseline. In sensitivity analyses, we adjusted for covariates measured before or at the start of morbidity and mortality follow-up to assess the robustness of the associations. Covariates included age, sex, education, marital status, employment during the 5-year exposure period, population density in the neighbourhood, and place of residence (urban area *vs* rural area). In a further sensitivity analysis based on the FPS study only, adjusted covariates included type of residence, total number of rooms in the residence, and floor area of residence. To further minimise bias due to differences in employment status, we excluded participants who were not fully employed during the 5-year exposure period from the sensitivity analysis.

In step 4, we assessed the association between change in neighbourhoods and change in lifestyle factors between year 1 (baseline) and year 4 or 5 using logistic regression analysis with the generalised estimating equations method for repeat data. These analyses also emulated non-randomised trial designs,^21^ assessing the impact of favourable neighbourhood change for participants with disadvantaged neighbourhoods and unhealthy lifestyle at baseline and of unfavourable neighbourhood change in those with advantaged neighbourhoods and a healthy lifestyle at baseline.

We also did three post-hoc analyses with alternative operationalisations for neighbourhood characteristics and their associations with the 79 health outcomes, including *Z* scores (mean 0 [SD 1]) for the level of, and change in, neighbourhood characteristics, and a single indicator of the number of different advantaged (above median) neighbourhood characteristics (range 0–4). In the first two analyses, HRs and 95% CIs were computed per 1 SD increase in continuous neighbourhood characteristics. In the third analysis, HRs and 95% CIs were computed per additional advantaged neighbourhood characteristic compared with none. These effect estimates were adjusted for age, sex, education, and cohort.

For analyses not corrected for multiple comparison, a two-sided p value of less than 0·05 was considered to indicate statistical significance. We did all analyses using SAS (version 9.4) and the statistical code used is provided in appendix 1 (pp 45–49).

### Role of the funding source

The funders of the study had no role in study design, data collection, data analysis, data interpretation, or writing of the report. All authors had final responsibility for the decision to submit for publication.

## Results

The eligible population included 178 375 people (120 192 [67·4%] women), of whom 91 131 individuals from the FPS study and 23 655 individuals from the HeSSup study (87 012 [75·8%] women; mean age 44·4 years [SD 11·1]) had complete data and thus were included in this cohort study (appendix 1 pp 13–14). Compared with the national mean for the Finnish population, study participants lived in more advantaged residential neighbourhoods.

**Figure 1:**
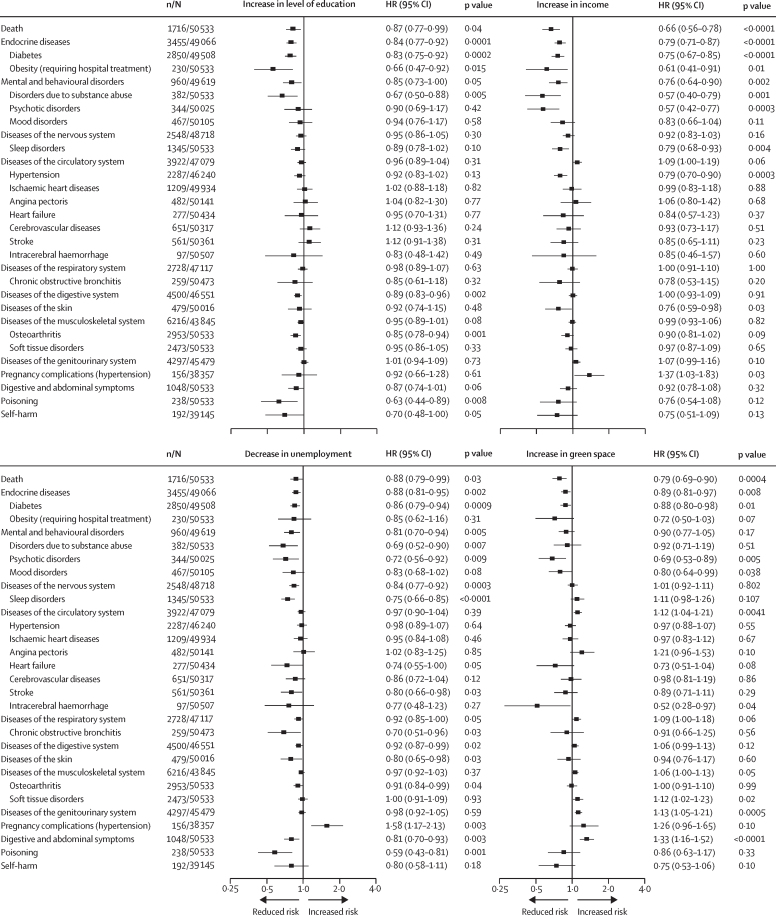
Association of favourable change in neighbourhood characteristics with subsequent health outcomes in participants residing in disadvantaged neighbourhoods at baseline* HR=hazard ratio. *HRs are adjusted for age, sex, education, and cohort.

**Figure 2:**
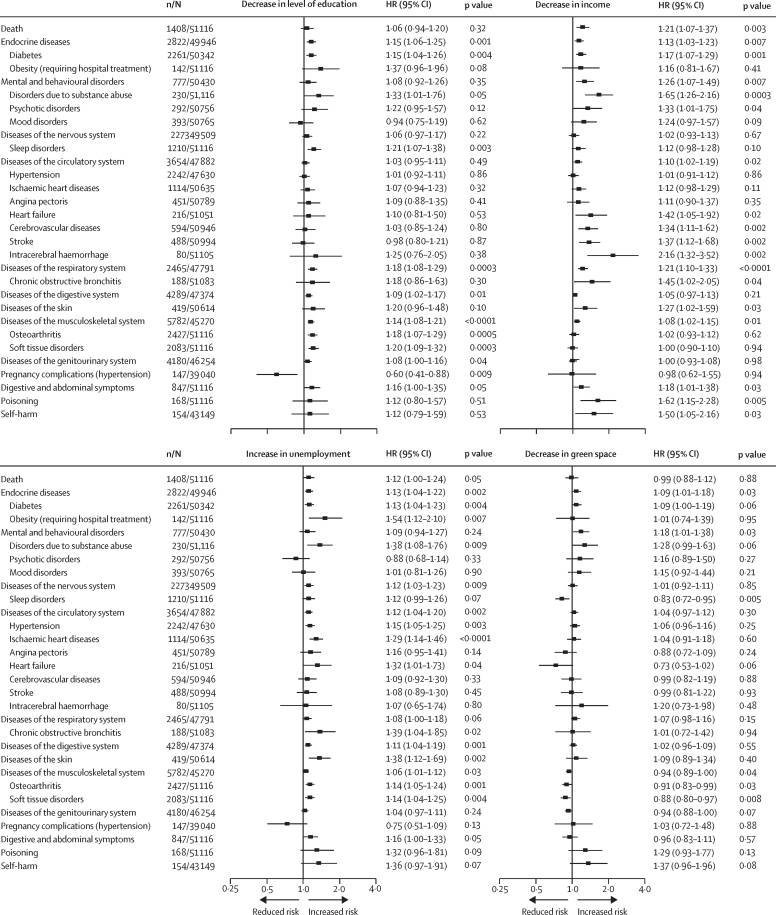
Association of unfavourable change in neighbourhood characteristics with subsequent health outcomes in participants residing in advantaged neighbourhoods at baseline* HR=hazard ratio. *HRs are adjusted for age, sex, education, and cohort.

During 1·17 million person-years at risk (median follow-up duration 14·0 years [IQR 9–14]), we recorded 164 368 new-onset health outcomes, including 3438 deaths. In the first analysis step including 79 endpoints, 30 health outcomes were associated with one or more neighbourhood characteristics after correction for multiple testing (table 1; appendix 1 pp 8, 15–19). In step 2, comparison of participants whose neighbourhood classification changed with those who remained in a similar neighbourhood showed mean favourable change in neighbourhood characteristics was associated with a reduced risk of death and 19 health outcomes (figure 1). Unfavourable changes in neighbourhood characteristics were associated with increased risk of death and increased risk of 27 health conditions or disease groups (figure 2). 14 health outcomes were associated with both favourable and unfavourable changes in neighbourhood characteristics in the total cohort or among individuals with unchanged residential address during the 5-year exposure period (death, mental and behavioural disorders, and specific diseases of the endocrine, nervous, respiratory, digestive, and musculoskeletal systems; appendix 1 pp 20–23).

**Table 1:** Associations between neighbourhood characteristics and new-onset diseases and health conditions*

		**n/N**	**HR (95% CI)**†			
			Education (high *vs* low)	Income (high *vs* low)	Unemployment (low *vs* high)	Green space (high *vs* low)
Death		3438/114 786	0·86 (0·80–0·92)	0·72 (0·67–0·77)	0·84 (0·78–0·90)	0·87 (0·81–0·93)
Infections		3032/112 644	0·97 (0·90–1·05)	0·92 (0·85–0·99)	0·96 (0·89–1·03)	0·93 (0·86–1·00)
Cancer		5502/112 666	1·02 (0·96–1·08)	1·03 (0·98–1·09)	1·01 (0·96–1·07)	1·06 (1·00–1·11)
Diseases of the blood		618/114 382	0·95 (0·80–1·12)	0·76 (0·64–0·90)	0·96 (0·81–1·13)	0·96 (0·82–1·12)
Endocrine diseases		6885/113 086	0·82 (0·78–0·86)	0·83 (0·79–0·88)	0·85 (0·81–0·90)	0·92 (0·87–0·96)
	Diabetes	5611/112 785	0·80 (0·76–0·85)	0·81 (0·77–0·86)	0·84 (0·80–0·89)	0·91 (0·87–0·96)
	Obesity (requiring hospital treatment)	415/114 786	0·61 (0·50–0·76)	0·73 (0·59–0·89)	0·69 (0·56–0·85)	0·94 (0·78–1·14)
Mental and behavioural disorders		1941/112 942	0·85 (0·77–0·93)	0·73 (0·66–0·80)	0·82 (0·75–0·90)	0·82 (0·75–0·90)
	Disorders due to substance abuse	696/114 786	0·64 (0·54–0·75)	0·61 (0·52–0·72)	0·70 (0·59–0·82)	0·85 (0·73–0·99)
	Psychotic disorders	720/113 800	0·88 (0·75–1·03)	0·63 (0·54–0·74)	0·83 (0·71–0·97)	0·70 (0·60–0·81)
	Mood disorders	951/113 892	0·94 (0·82–1·08)	0·75 (0·66–0·86)	0·85 (0·74–0·97)	0·79 (0·69–0·90)
Diseases of the nervous system		5426/110 948	0·93 (0·87–0·98)	0·96 (0·90–1·01)	0·86 (0·81–0·91)	1·03 (0·97–1·08)
	Sleep disorders	2846/114 786	0·82 (0·76–0·89)	0·84 (0·78–0·91)	0·81 (0·75–0·87)	1·07 (0·99–1·15)
Diseases of the eye		5472/113 149	0·99 (0·94–1·05)	0·94 (0·89–1·00)	0·98 (0·92–1·03)	0·96 (0·91–1·01)
Diseases of the ear		930/113 829	0·93 (0·81–1·07)	0·92 (0·81–1·06)	0·83 (0·72–0·95)	1·02 (0·89–1·16)
Diseases of the circulatory system		8410/107 313	0·94 (0·90–0·98)	0·95 (0·90–0·99)	0·89 (0·85–0·93)	1·03 (0·99–1·08)
	Hypertension	5008/106 221	0·95 (0·90–1·01)	0·98 (0·92–1·03)	0·89 (0·84–0·95)	1·02 (0·96–1·07)
	Ischaemic heart diseases	2558/113 585	0·95 (0·88–1·03)	0·95 (0·87–1·03)	0·81 (0·74–0·88)	1·02 (0·95–1·11)
	Angina pectoris	1036/113 988	0·97 (0·85–1·10)	1·08 (0·94–1·23)	0·88 (0·77–1·00)	1·27 (1·12–1·43)
	Heart failure	539/114 604	0·84 (0·70–1·00)	0·69 (0·57–0·82)	0·72 (0·60–0·87)	1·00 (0·84–1·18)
	Cerebrovascular diseases	1382/114 346	1·00 (0·89–1·12)	0·77 (0·69–0·86)	0·85 (0·76–0·96)	0·94 (0·84–1·04)
	Stroke	1155/114 449	0·98 (0·87–1·11)	0·75 (0·66–0·84)	0·82 (0·73–0·93)	0·91 (0·81–1·02)
	Intracerebral haemorrhage	191/114 738	0·78 (0·58–1·06)	0·54 (0·40–0·73)	0·72 (0·53–0·96)	0·68 (0·51–0·91)
Diseases of the respiratory system		5882/106 991	0·89 (0·85–0·94)	0·85 (0·81–0·90)	0·90 (0·85–0·95)	0·96 (0·91–1·01)
	Chronic obstructive bronchitis	491/114 681	0·80 (0·66–0·97)	0·55 (0·45–0·67)	0·67 (0·56–0·82)	0·77 (0·64–0·92)
Diseases of the digestive system		9909/106 081	0·89 (0·85–0·93)	0·97 (0·93–1·01)	0·90 (0·86–0·94)	1·03 (0·99–1·08)
Diseases of the skin		1008/113 627	0·87 (0·76–0·99)	0·81 (0·71–0·92)	0·73 (0·64–0·83)	0·99 (0·88–1·13)
Diseases of the musculoskeletal system		13 435/100 749	0·90 (0·86–0·93)	1·02 (0·98–1·06)	0·94 (0·91–0·98)	1·11 (1·07–1·14)
	Osteoarthritis	5964/114 786	0·81 (0·77–0·85)	0·97 (0·92–1·02)	0·87 (0·82–0·91)	1·09 (1·04–1·15)
	Soft tissue disorders	5128/114 786	0·87 (0·82–0·92)	1·05 (0·99–1·12)	0·92 (0·87–0·97)	1·18 (1·11–1·24)
Diseases of the genitourinary system		9517/103 653	0·95 (0·91–0·99)	1·04 (1·00–1·09)	0·96 (0·92–1·01)	1·10 (1·06–1·15)
Pregnancy complications (hypertension)		377/87 012	1·19 (0·96–1·48)	1·22 (0·98–1·53)	1·47 (1·19–1·83)	1·02 (0·83–1·25)
Digestive and abdominal symptoms		2160/114 786	0·84 (0·76–0·92)	0·89 (0·81–0·97)	0·82 (0·75–0·90)	1·13 (1·04–1·23)
Poisoning		453/114 786	0·73 (0·59–0·89)	0·61 (0·50–0·75)	0·63 (0·52–0·78)	0·76 (0·63–0·91)
Self-harm		369/91 131	0·78 (0·63–0·97)	0·65 (0·52–0·80)	0·71 (0·58–0·89)	0·67 (0·54–0·83)

HR=hazard ratio. ICD-10=International Classification of Diseases, Tenth Revision. Detailed results for all 79 health outcomes are provided in appendix 1 (pp 15–18).

* All disease groups (ICD-10 chapters) are presented, but only specific health conditions that were associated with any of the neighbourhood characteristics after Bonferroni correction are shown.

† Adjusted for age, sex, education, and cohort.

**Figure 3:**
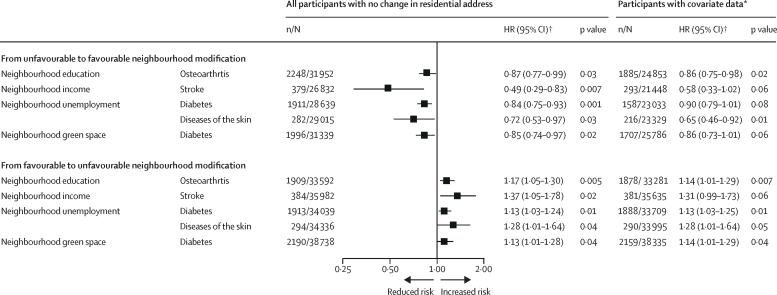
Association of change in neighbourhood characteristics with subsequent health outcomes among participants with no change in residential address during the 5-year exposure period HR=hazard ratio. *Adjusted for age, sex, education, and cohort. †Adjusted for age, sex, education, marital status, population density in the neighbourhood, place of residence (urban *vs* rural), and being in employment during the 5-year exposure period.

Comparison of disease risk between participants who moved residential address and those who did not move during the 5-year exposure period is shown in appendix 1 (pp 24–32). In the subgroup analyses of participants who did not move residential address, differences in age and sex between the groups were small; however, individuals exposed to favourable neighbourhood modifications or those who exclusively resided in advantaged neighbourhoods were more likely to have a higher level of education than those exposed to unfavourable neighbourhood modification or those who remained in disadvantaged neighbourhoods (appendix 1 p 33). After adjustment for age, sex, cohort, and education, four of the 14 health outcomes were associated with both an improvement and worsening in neighbourhood characteristics (figure 3): people residing in neighbourhoods where elevations in educational level were observed subsequently had a lower risk of osteoarthritis compared with those who did not reside in areas with such changes (HR 0·87, 95% CI 0·77–0·99; p=0·03). Individuals living in neighbourhoods with increasing income levels had a reduced risk of stroke (0·49, 0·29–0·83; p=0·007). Reductions in unemployment in residential area were associated with a reduced risk of type 2 diabetes (0·84, 0·75–0·93; p=0·001) and reduced risk of skin disease (0·72, 0·53–0·97; p=0·03), and increases in neighbourhood green space were associated with reduced risk of type 2 diabetes (0·85, 0·74–0·97; p=0·02). Adverse changes in these neighbourhood characteristics were associated with increased risk of these health outcomes. The estimates remained similar after additional adjustments for individual-level and residence-level characteristics (marital status, employment status during the exposure period, population density in the neighbourhood, and place of residence [urban *vs* rural]; figure 3) in a subpopulation of individuals who did not move address and were in stable employment throughout the exposure period, in separate analyses of men and women, and after adjustment for additional covariates available only in the FPS study (appendix 1 pp 34–37). With a broader index of neighbourhood characteristics based on 750 m × 750 m rather than 250 m × 250 m grids, the direction of associations were consistent, but with smaller effect sizes (appendix 1 pp 38–41).

The associations between neighbourhood characteristics and health outcomes seemed to coincide with changes in lifestyle factors (the fourth analysis step). Among participants who did not move residential address and had two or more lifestyle risk factors (smoking, heavy drinking, physical inactivity, or obesity) at baseline, a decline in neighbourhood unemployment rate and an increase in green space were associated with a greater likelihood of reducing the number of these lifestyle risk factors at follow up to 0 or 1 (table 2). Unfavourable changes in neighbourhood unemployment rate and green space and in the educational level of residents were associated with increasing likelihood of having multiple risk factors at follow-up among individuals who originally had none or only one risk factor (table 3). Sensitivity analyses of subgroups showed that changes in participants’ employment status or retirement (appendix p 42) and confounding by place of residence (appendix p 43) were unlikely explanations for these findings.

**Table 2:** Association between favourable changes in neighbourhood characteristics and lifestyle factors in a subgroup of participants who did not move residential address during the study period and had an unhealthy lifestyle and lived in a disadvantaged residential neighbourhood at baseline

	**Quitting smoking among individuals who were current smokers at baseline**		**Drinking reduced to none or moderate in individuals who were heavy drinkers at baseline**		**Increased physical activity in individuals who were physically inactive at baseline**		**≥5% bodyweight loss in individuals who were overweight or people with obesity at baseline**		**Change from having ≥2 lifestyle risk factors to having 0 or 1 lifestyle risk factor**	
	n/N (%)	OR (95% CI)*	n/N (%)	OR (95% CI)*	n/N (%)	OR (95% CI)*	n/N (%)	OR (95% CI)*	n/N (%)	OR (95% CI)*
**Neighbourhood education**										
Stable disadvantaged	1588/6924 (22·9%)	1 (ref)	2800/7931 (35·3%)	1 (ref)	4630/9741 (47·5%)	1 (ref)	3240/21 620 (15·0%)	1 (ref)	3065/8058 (38·0%)	1 (ref)
From disadvantaged to advantaged	227/891 (25·5%)	1·11 (0·94–1·31)	453/1349 (33·6%)	0·92 (0·81–1·04)	645/1289 (50·0%)	1·06 (0·94–1·19)	384/3047 (12·6%)	1·19 (1·06–1·33)	401/994 (40·3%)	1·09 (0·95–1·25)
**Neighbourhood income**										
Stable disadvantaged	1527/6780 (22·5%)	1 (ref)	2793/8333 (33·5%)	1 (ref)	4172/8993 (46·4%)	1 (ref)	2944/19 868 (14·8%)	1 (ref)	2876/7797 (36·9%)	1 (ref)
From disadvantaged to advantaged	164/553 (29·7%)	1·38 (1·14–1·67)	278/772 (36·0%)	1·06 (0·90–1·24)	402/770 (52·2%)	1·12 (0·97–1·31)	226/1642 (13·8%)	1·05 (0·91–1·22)	251/600 (41·8%)	1·15 (0·97–1·37)
**Neighbourhood unemployment**										
Stable disadvantaged	1281/5799 (22·1%)	1 (ref)	2327/6948 (33·5%)	1 (ref)	3608/7771 (46·4%)	1 (ref)	2487/17 215 (14·5%)	1 (ref)	2450/6604 (37·1%)	1 (ref)
From disadvantaged to advantaged	408/1519 (26·9%)	1·24 (1·09–1·42)	816/2385 (34·2%)	1·01 (0·92–1·12)	1272/2449 (51·9%)	1·18 (1·08–1·30)	809/5599 (14·5%)	0·99 (0·91–1·08)	756/1866 (40·5%)	1·11 (1·00–1·23)
**Neighbourhood green space**										
Stable disadvantaged	1528/6621 (23·1%)	1 (ref)	3024/9173 (33·0%)	1 (ref)	4524/9422 (48·0%)	1 (ref)	3058/20 751 (14·7%)	1 (ref)	2928/7794 (37·6%)	1 (ref)
From disadvantaged to advantaged	205/819 (25·0%)	1·12 (0·94–1·32)	371/1092 (34·0%)	1·04 (0·91–1·19)	604/1180 (51·2%)	1·11 (0·98–1·27)	409/2737 (14·9%)	0·97 (0·87–1·09)	394/914 (43·1%)	1·24 (1·08–1·43)

OR=odds ratio.

* Calculated from logistic regression analysis using generalised estimating equations, adjusted for age, sex, education, survey pair, and cohort.

**Table 3:** Association between unfavourable changes in neighbourhood characteristics and lifestyle factors in a subgroup of participants who did not move residential address during the study period and who had a healthy lifestyle and lived in an advantaged residential neighbourhood at baseline

	**Smoking relapse in individuals who were ex–smokers at baseline**		**Heavy drinking in individuals who were moderate or non-drinkers at baseline**		**Physical inactivity in individuals who were physically active at baseline**		**≥5% bodyweight gain among individuals who did not have obesity at baseline**		**Change from having 0 or 1 lifestyle risk factor to having ≥2 lifestyle risk factors**	
	n/N (%)	OR (95% CI)*	n/N (%)	OR (95% CI)*	n/N (%)	OR (95% CI)*	n/N (%)	OR (95% CI)*	n/N (%)	OR (95% CI)*
**Neighbourhood education**										
Stable advantaged	430/7559 (5·7%)	1 (ref)	2533/34 032 (7·4%)	1 (ref)	4592/34 722 (13·2%)	1 (ref)	8246/36 728 (22·5%)	1 (ref)	2508/37 446 (6·7%)	1 (ref)
From advantaged to disadvantaged	96/1515 (6·3%)	1·06 (0.84–1·34)	497/6389 (7·8%)	1·01 (0·91–1·11)	947/6323 (15·0%)	1·11 (1·03–1·20)	1618/6700 (24·2%)	1·04 (0·98–1·11)	586/6803 (8·6%)	1·23 (1·12–1·35)
**Neighbourhood income**										
Stable advantaged	466/8512 (5·5%)	1 (ref)	2728/38 005 (7·2%)	1 (ref)	5115/37 796 (13·5%)	1 (ref)	9065/39 916 (22·7%)	1 (ref)	2861/41 102 (7·0%)	1 (ref)
From advantaged to disadvantaged	74/1060 (7·0%)	1·26 (0·97–1·64)	339/4439 (7·6%)	1·02 (0·91–1·15)	647/4374 (14·8%)	1·06 (0·97–1·16)	1138/4629 (24·6%)	1·08 (1·00–1·16)	382/4700 (8·1%)	1·11 (0·99–1·24)
**Neighbourhood unemployment**										
Stable advantaged	362/6748 (5·4%)	1 (ref)	2251/30 029 (7·5%)	1 (ref)	4089/30 137 (13·6%)	1 (ref)	7356/31 821 (23·1%)	1 (ref)	2262/32 558 (7·0%)	1 (ref)
From advantaged to disadvantaged	172/2617 (6·6%)	1·21 (1·00–1·47)	813/10 991 (7·4%)	0·97 (0·89–1·05)	1567/10 783 (14·5%)	1·05 (0·98–1·12)	2723/11 460 (23·8%)	0·94 (0·88–1·01)	928/11 699 (7·9%)	1·10 (1·02–1·19)
**Neighbourhood green space**										
Stable advantaged	549/9750 (5·6%)	1 (ref)	2946/41 768 (7·1%)	1 (ref)	5975/40 464 (14·8%)	1 (ref)	10 170/43 013 (23·6%)	1 (ref)	3319/44 111 (7·5%)	1 (ref)
From advantaged to disadvantaged	92/1226 (7·5%)	1·28 (1·02–1·62)	375/5317 (7·1%)	0·97 (0·87–1·09)	761/5214 (14·6%)	1·00 (0·92–1·09)	1266/5531 (22·9%)	0·94 (0·88–1·01)	470/5674 (8·3%)	1·11 (1·00–1·23)

OR=odds ratio.

* Calculated using logistic regression analysis with generalised estimating equations, adjusted for age, sex, education, survey pair, and cohort.

Continuous measures of neighbourhood characteristic levels were associated with 34 health outcomes after correction for multiple testing, including 28 of the 30 outcomes identified in step 1. Of the 34 outcomes, 14 outcomes were also consistently associated with continuously measured change in neighbourhood characteristics, which included cardiometabolic, musculoskeletal, neural, mental, and digestive diseases, but not skin diseases. The number of favourable neighbourhood characteristics was associated with 36 health outcomes in a dose-response manner (p<0**·**05). A data atlas for all associations with 79 health outcomes is available in appendix 2.

## Discussion

A major advantage of this outcome-wide study was the use of longitudinal data to emulate a non-randomised trial of neighbourhood modification. Using this approach, we found that compared with people continually living in disadvantaged neighbourhoods, those who resided throughout the study in neighbourhoods that transitioned over time toward more favourable characteristics, in terms of educational levels, income, and employment rates of residents and improvements in green space, had a lower future risk of diabetes, stroke, skin disease, and osteoarthritis. Among individuals who lived in advantaged residential environments at baseline, unfavourable modifications to neighbourhoods were associated with increased risk of diabetes, stroke, skin disease, and osteoarthritis. Parallel changes in selected individual-level health risk factors, including the proportion of people who quit smoking, reduced their alcohol consumption, increased their physical activity, and who lost 5% or more of their bodyweight, support the plausibility of these associations. Since the associations between neighbourhood characteristics and health outcomes were observed among individuals who did not move during the entire observation period, our findings are likely to reflect effects of modifications to neighbourhoods.

Many cross-sectional and prospective studies have reported associations between neighbourhood characteristics and health outcomes although these studies typically did not include data on modifications to local residential areas.^4^, ^5^, ^6^, ^8^, ^9^, ^10^, ^22^, ^23^ In our epidemiological analysis adjusted for multiple testing, we found that neighbourhood characteristics were associated with more than a third (ie, death and 29 diseases) of the 79 health outcomes studied. Almost all of these health outcomes were also associated with favourable or unfavourable changes in neighbourhood characteristics. The most robust associations were observed in diabetes, stroke, skin diseases, and osteoarthritis outcomes that were associated with both favourable and unfavourable local modifications in neighbourhood characteristics.

Our findings on the potential health benefits of high levels of employment, educational attainment, income, and green space in residential areas are consistent with the hypothesis that the attraction of employed, well educated people to a residential area might have various positive effects that contribute to several aspects of health for individuals who already live in the area.^24^ We found that a decreased neighbourhood unemployment rate was associated with a lower risk of diabetes (HR 0·84; 95% CI 0·75–0·93), which is consistent with the findings of two intervention studies on specific subpopulations (families receiving a rent-subsidy voucher and refugees), which found around 20% reductions in diabetes risk when participants moved from a disadvantaged area to a less disadvantaged residential neighbourhood.^1^, ^2^ Our finding regarding diabetes risk is also consistent with a meta-analysis of six studies that reported a 10% lower diabetes prevalence in residential areas with more green space, however, the confidence interval for this estimate was wide.^25^ Our findings on stroke risk support previous results linking neighbourhood income and unemployment rates to this health outcome in western countries.^26^

A number of mechanisms might underlie our observations. In accordance with changes in neighbourhoods, we observed alterations in lifestyle-related behavioural risk factors that might have contributed to associations with health outcomes. For example, the observed reductions in smoking could affect stroke incidence. Plausible neighbourhood contributors that led to this lifestyle change might include social influences of groups of interconnected people who stop smoking in unison^27^ and lower density of tobacco and alcohol outlets in the residential area.^28^ The wider uptake of a physically active lifestyle following residential area changes, potentially facilitating reduced incidence of diabetes^29^ and osteoarthritis,^30^ could result from improvements in facilities for leisure activities and person-to-person encouragement of a more physically active lifestyle.^15^ Detailed analysis of specific diagnoses suggest that poor neighbourhood cleanliness and hygiene might contribute to the association between neighbourhoods with high unemployment rates and increased risk of skin diseases (appendix 1 p 44).

Our study has several strengths. The application of a data-driven approach to a large number of health outcomes enabled a more comprehensive assessment of morbidity than that obtained from traditional analyses of disadvantaged neighbourhoods. Further studies could expand this approach to cover multimorbidity considering the paucity of information available about the extent to which neighbourhood characteristics contribute to the coexistence of more than one health condition. Other strengths included a study design that emulated neighbourhood modification trials; separation of neighbourhood modifications from those resulting from participants’ moving to another residential address; a large sample size with data on a large number of covariates; and objective, high spatial resolution of neighbourhood characteristics. These features enabled the investigation and demonstration of biases, such as health-related selection, which, for example**,** might have artificially inflated associations between neighbourhood characteristics and disorders due to substance abuse.

This study also had limitations. No observational data can prove causality due to possible residual confounding and bias. Although the possibility of residual confounding and bias was reduced in this present study by analysing the data as non-randomised nested pseudo-trials,^21^ we could not fully exclude the possibility of health-related selection that might have arisen—eg, from educational differences in participants staying in advantaged or improving neighbourhoods. Our main analyses were based on dichotomised neighbourhood characteristics, an approach that minimises the likelihood of overestimating associations but might miss moderate and weak effects. Post-hoc analyses based on continuous variables identified eight additional neighbourhood-health outcome associations compared with the main analysis (38 *vs* 30) after similar adjustments for multiple comparisons (appendix 2). Public and private green space might impact health outcomes in different ways (eg, public green spaces better support recreational physical activity than private green spaces); however, we were not able to differentiate between public and private green spaces in the present analyses. Data on lifestyle factors were self-reported and thus subject to measurement error. Follow-up based on electronic health records in the present study in a country with a comprehensive health-care system had high coverage, but undiagnosed morbidity and illness diagnosed and managed in primary care would have been missed. Our study sample was ethnically homogeneous, more privileged than the general population, and from a single country with a lower population density and more green spaces than in most European countries, which might limit the generalisability of the findings.

In conclusion, the results of this data-driven study suggest that favourable modifications to residential neighbourhoods might contribute to healthy lifestyle choices and could lead to potentially important reductions in a range of specific morbidities over time. The main findings were based on analyses that emulated non-randomised neighbourhood modification trials and highlighted four health outcomes that were most robustly associated with changes in neighbourhood characteristics.Our data atlas including all prospective associations between four major neighbourhood characteristics and 79 common health outcomes among 100 000 community-dwelling individuals provides a more inclusive reference database for future studies on the potential health effects of residential neighbourhoods.

## Data sharing

Statistical code is provided in appendix 1 (pp 45–49). The pseudonymised questionnaire data used in the FPS and HeSSup studies can be shared by request to the investigators and after approval of the Finnish Institute of Occupational Health and HeSSup study scientific committees. Linked electronic health records require separate permission from the National Institute of Health and Welfare and Statistics Finland.

## Declaration of interests

We declare no competing interests.
