# Supplementary material for: Modifications to residential neighbourhood characteristics and risk of 79 common health conditions: a prospective cohort study
Source: Lancet Public Health. 2021 May 26;6(6):e396–407. doi: 10.1016/S2468-2667(21)00066-9 (PMC8172714; doi:10.1016/S2468-2667(21)00066-9)
Supplement: Supplementary appendix 1 [file mmc1.pdf]

THE LANCET
Public Health

Supplementary appendix 1

This appendix formed part of the original submission and has been peer reviewed. We post it as supplied by the authors.

Supplement to: Kivimäki M, Batty GD, Pentti J, et al. Modifications to residential neighbourhood characteristics and risk of 79 common health conditions: a prospective cohort study. *Lancet Public Health* 2021; **6:** 396–407.

CONTENTS OF APPENDIX 1

eFigure 1. Sample selection and analytic steps for study of health outcomes and for study of lifestyle change…… 3
Description of study population…… 4
eFigure 2. Timeline for data collections by cohort…… 4
eFigure 3. Geocoded map of the residential addresses of the participants…… 5
Further details on the assessment of green space…… 5
Further details about lifestyle risk factors…… 6
Further details about morbidity assessment…… 6
eTable 1. Included ICD-10 chapters and codes and exclusions for baseline cases…… 6
Further details about correction for multiple testing…… 7
Summary of supplementary results …… 8
eTable 2. Steps of analysis, number of tests conducted, and statistically significant findings…… 10
eTable 3. Test of proportional hazards assumption for associations between 4 neighbourhood characteristics and 79 health outcomes…… 11
eFigure 4. Log-log plots of non-proportional neighbourhood characteristic-health outcome associations…… 12
eTable 4. Characteristics of the study population by sample…… 13
eTable 5. Characteristics of the study population by cohort…… 14
eTable 6. Associations between neighbourhood characteristics [median split] and 79 health outcomes after adjustment for age, sex, education and cohort…… 15
eTable 7. Statistical significance of the 'neighbourhood characteristic x cohort' interaction terms on 79 health outcomes…… 19
eTable 8. Association of favourable and unfavourable change in neighbourhood characteristics with 30 subsequent health outcomes…… 20
eTable 9. Prevalent and new diseases in participants who moved (N = 42 916) versus those who did not move residential address (N = 71 705)…… 24
eTable 10. Association of favourable and unfavourable change in neighbourhood characteristics with 30 subsequent health outcomes in non-movers…… 25
eTable 11. Association of favourable and unfavourable change in neighbourhood characteristics with 30 subsequent health outcomes in movers…… 29
eTable 12. Baseline characteristics of the study population in 8 non-randomised modifications to neighbourhood characteristics in participants who did move residence…… 33
eFigure 5. Association of change in neighbourhood characteristics with health outcomes among participants with no change in residential address or employment…… 34
eTable 13. Association of favourable and unfavourable change in neighbourhood characteristics with subsequent health outcomes in non-movers by sex…… 35
eTable 14. Association of favourable and unfavourable change in neighbourhood characteristics with subsequent health outcomes among non-movers who had more than one lifestyle risk factors and lived in urban areas…… 36
eTable 15. Association of favourable and unfavourable change in neighbourhood characteristics with subsequent health outcomes in non-movers after multivariable adjustments…… 37

eTable 16. Association of favourable and unfavourable change in 750m x 750m grid neighbourhood characteristics with 30 subsequent health outcomes in non-movers…………………. 38
eTable 17. Association of favourable change in neighbourhood characteristics and lifestyle factors in participants with unhealthy lifestyle and disadvantaged neighbourhood and association of unfavourable change in neighbourhood characteristics and lifestyle factors in participants with healthy lifestyle and advantaged neighbourhoods in a population with stable employment……. 42
eTable 18. Association of favourable change in neighbourhood characteristics and lifestyle factors in participants with unhealthy lifestyle and disadvantaged neighbourhood and association of unfavourable change in neighbourhood characteristics and lifestyle factors in participants with healthy lifestyle and advantaged neighbourhoods in a population living in urban areas ………. 43
Supplementary analyses on skin diseases in relation to neighbourhood unemployment……………. 44
eTable 19. Cases of skin disease among non-movers by neighbourhood change category…………. 44
Statistical code……. 45
References……. 50

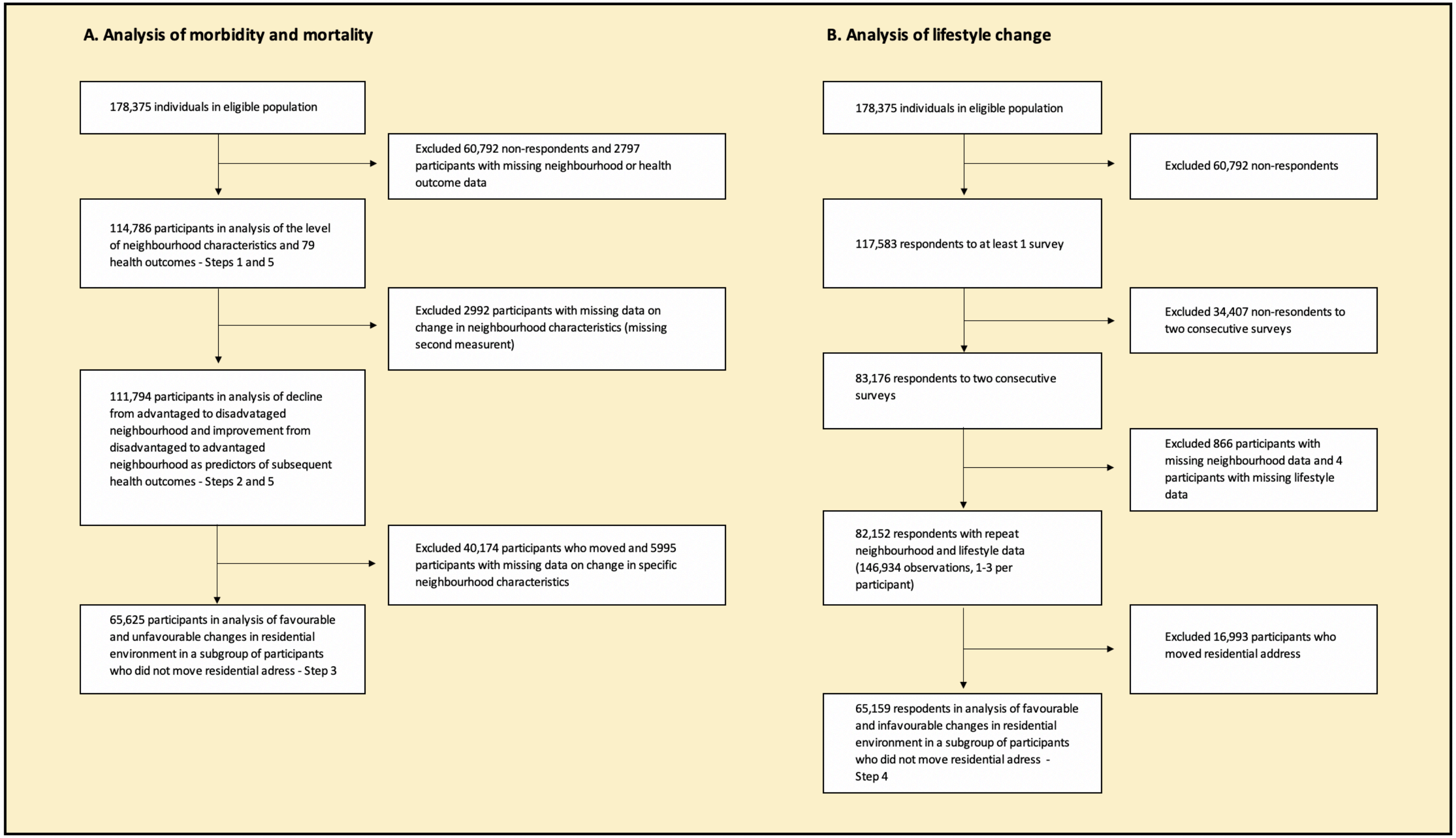

eFigure 1. Sample selection and analytic steps for study of health outcomes (A) and for study of lifestyle change (B)

DESCRIPTION OF STUDY POPULATION

Men and women participating in HeSSup were from a stratified random sample of the Finnish population based on four age groups (20-24, 30-34, 40-44, and 50-54). The eligible population (N=64,797) was identified from the Finnish population register and an invitation to participate was posted for them, along with a baseline questionnaire.[1] Between June 7, 1998 and May 23, 1999 and January 7 and August 12, 2003, a total of 23,655 responded had data on residential neighbourhoods and were successfully linked to electronic health records of national registers until December 31, 2012. The Turku University Central Hospital Ethics Committee approved the study.

The FPS sample comprised the entire public sector personnel of 10 cities and 21 hospitals in the same geographical areas.2 A total of 113,578 participants had a job contract of at least 6 months between 1990 and 2005 and were eligible for at least one of the four surveys conducted between March 1, 2000 and June 30, 2002; March 1, 2004 and June 30, 2005; March 1, 2008 and November 30, 2009, December 1, 2011 and November 30, 2013. The sample included in the present analysis comprised 91,131 men and women aged 17 to 77 who responded to the survey, had data on residential neighbourhoods, and were successfully linked to electronic health records from national registries up to December 31, 2018. Corresponding to the sex distribution of Finnish public sector employees, almost 80% of the participants were women.

eFigure 2 shows timeline for data collection for study of neighbourhood characteristics, morbidity and mortality and that of neighbourhood characteristics and lifestyle changes by type of data in both cohort studies. The 5-year time lag is commonly used in prognostic studies and it is also the frequency for health checks in the general population that aim to prevent disease and premature mortality.[2,3] **eFigure 3** shows geocoded locations of the participants, including a total of 16,208 locations for the participants of the HeSSup study and 23,122 locations for those of FPS. Mean (SD) number of participants per location was 1.4 (1.2) in HeSSup and 3.9 (6.4) in FPS.

eFigure 2. Timeline for data collections by cohort

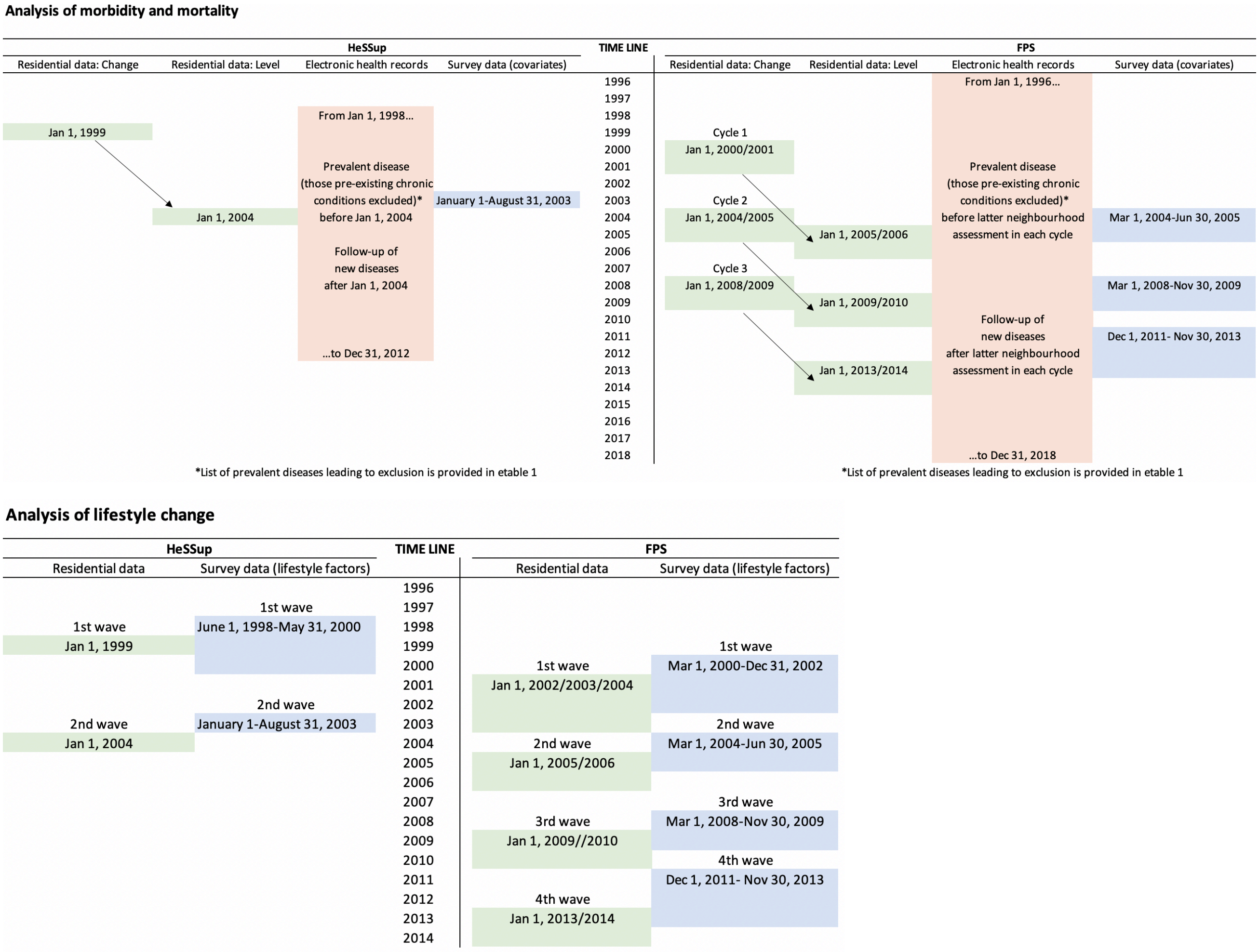

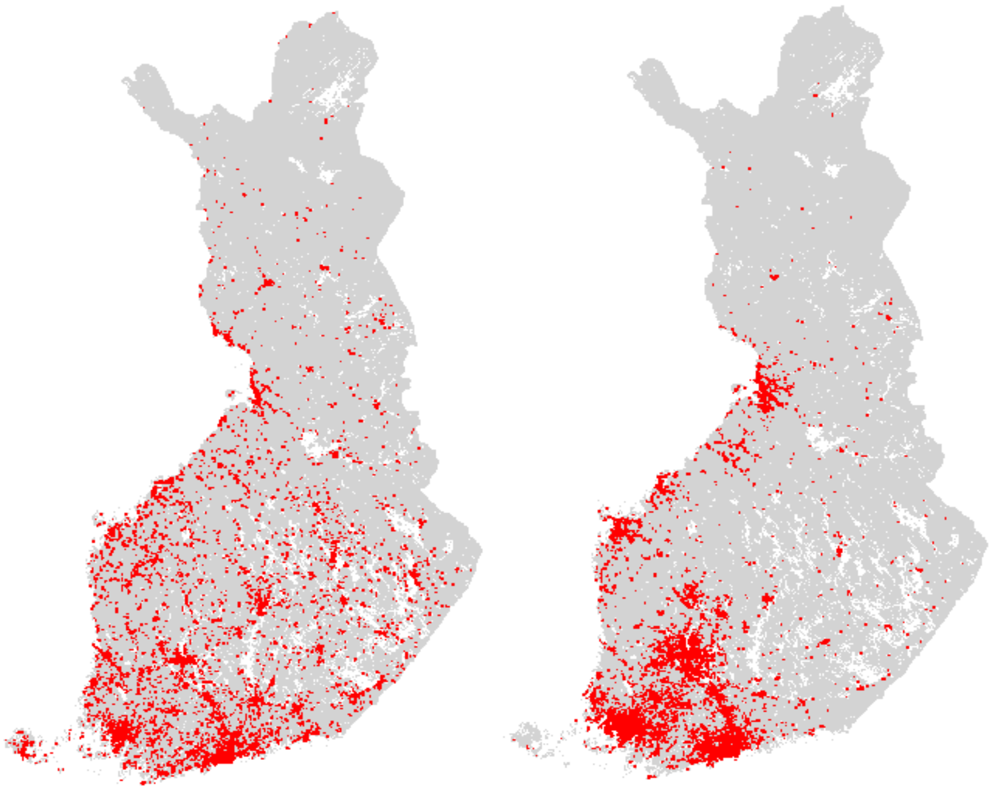

eFigure 3. Geocoded map of participants' residential unit locations (in red) in Finland by cohort at start of health follow-up

FURTHER DETAILS ON THE ASSESSMENT OF GREEN SPACE

Green space was defined as any open land surface that was partly or completely covered with grass, trees, shrubs, or other vegetation, and included, for instance, parks, forests and community gardens.[4] A national wide normalized difference vegetation index (NDVI) map was generated, as the residential places used in the analyses were located in different part of Finland. NDVI dataset was computed using 30mx30m resolution Landsat 5 Thematic Mapper images available in Google Earth Engine.[5] We selected images with maximum 30 percent of cloud cover between June and August (the maximum vegetation greenness period of the year in Finland). To minimise NDVI value variation due to atmospheric conditions, we used atmospherically corrected surface reflectance images. Water bodies and clouds were masked out in each images using the Quality Assessment band of Landsat 5. NDVI was estimate using the visible Red and Near Infrared (NIR) bands as NIR-Red/NIR+Red.[6] As the water bodies has been masked out, the NDVI values ranged from 0 to 1, where 0 denotes the lowest vegetation greenness or non-vegetation cover and 1 the highest cover.

As a single year collection of Landsat 5 TM images with a maximum of 30 percent of cloud cover during the maximum vegetation greenness period did not cover the whole study region, we used images of consequent three years period to generate a cloud-free composite maps of NDVI median values for the whole Finland in the study period. We assumed that urban green areas, mostly forest and grass, do not exhibit substantial inter-annual NDVI changes in short period, unless the vegetation cover has changed into other cover type, e.g. built-up area. The created NDVI maps were exported from Google Earth Engine as geotiff files. Statistical metrics of NDVI maps in 250x250m grid, used to assess the degree of residential greenspace, were estimated using R software in the Finnish CSC's high-performance supercomputer.

We validated NDVI data by comparisons with green space in high-resolution Aerial Photograph data in major Finnish cities. Indeed, NDVI is a widely applied technique for vegetation mapping[6-8] in epidemiological studies.[9,10]

FURTHER DETAILS ABOUT LIFESTYLE RISK FACTORS

We repeatedly assessed the following lifestyle factors at years 1 and 4 or 5 using identical standard survey instruments in both cohorts:[11] smoking status (current, ex- or never smoker); alcohol consumption (heavy [>14 units of ethanol for women and >21 units for men per week; 1unit=10g of ethanol], moderate [1-14

units for women and 1-21 units for men] or non-drinker);[12] physical activity (low [average energy expenditure, expressed as Metabolic Equivalent of Task MET-hours <14 per week], moderate [≥14 to<30 MET-h/week] or high [≥30 MET-h/week]);[13] and body mass index (obese [BMI≥30kg/m^2], overweight [BMI≥25 to <30 kg/m^2] or normal weight [BMI<25kg/m^2]) and a ≥5% weight change between years 1 and 4 or 5.

FURTHER DETAILS ABOUT MORBIDITY ASSESSMENT

Participants were linked by their unique identification number to national registries of hospital discharge information (recorded by the National Institute for Health and Welfare) and mortality (recorded by Statistics Finland). These electronic health records include cause and date of hospitalisation or mortality and their coverage - all hospital types, including private hospitals, and records cover emergencies - reflects the comprehensive nature of Finland's public health-care system. Additional information on site-specific cancers, diabetes, cardiovascular disease (including hypertension), psychotic disorders, dementia, Parkinson's disease, multiple sclerosis, epilepsy, asthma, chronic obstructive bronchitis, inflammatory bowel disease, liver disease, rheumatoid arthritis, gout, and renal failure was available via record linkage to the Drug Reimbursement Register of the Social Insurance Institution of Finland. The diagnosis for incident disease was coded according to the World Health Organization's International Classification of Diseases Tenth Revision (ICD-10).[14]

As previously,[15] we focused on fifteen ICD-10 disease chapters that concern infectious and parasitic diseases (A00-B99), neoplasms (C00-D48), diseases of the blood (D50-D89), endocrine, nutritional, and metabolic diseases (E00-E90), mental and behavioural disorders (F00-F99), diseases of the nervous system (G00-G99), the eye (H00-H59), the ear (H60-H95), the circulatory system (I00-I99), the respiratory system (J00-J99), the digestive system (K00-K93), the skin (L00-L99), the musculoskeletal system (M00-M99), and the genitourinary system (N00-N99), injuries and poisoning (S00-T98), and external causes (V01-Y98).[14]

Participants with a history of a chronic disease were excluded as shown in **eTable 1**.

eTable 1. Included ICD-10 chapters and codes and exclusions for baseline cases

ICD-10 Chapter		ICD-10 code	Baseline cases excluded*
I	**Infections**	**A01-B89**	*
	Bacterial infections	A01-A79	
	Viral infections	A80-B34	
II	**Cancer**	**C00–C97**	*
	Colorectal cancer	C18, C20	*
	Lung cancer	C34	*
	Melanoma	C43–C44	*
	Breast cancer	C50	*
	Prostate cancer	C61	*
	Kidney cancer	C64	*
	Brain cancer	C71	*
	Leukaemia, lymphoma	C81–C96	*
III	**Diseases of the blood**	**D50–D89**	*
	Anaemias	D50-D64	
IV	**Endocrine diseases**	**E00–E90**	*
	Diabetes	E10-E14	*
	Obesity	E66	
V	**Mental and behavioural disorders**	**F00–F99**	*
	Dementia	F00–F03, G30, G31	*
	Disorders due to substance abuse	F10–F19	
	Psychotic disorders	F20–F29	*
	Mood disorders	F30–F39	*
	Neurotic disorders	F40–F48	*
VI	**Diseases of the nervous system**	**G00–G99**	*
	Parkinson disease	G20	*
	Multiple sclerosis	G35	*
	Epilepsy	G40-G42	*
	Headaches	G43-G44	
	TIA	G45-G46	*
	Sleep disorders	G47	

**VII**	**Diseases of the eye**	**H00–H59**	*
**VIII**	**Diseases of the ear**	**H60–H99**	*
**IX**	**Diseases of the circulatory system**	**I00-I99**	*
	Hypertension	I10–I15	*
	Ischemic heart disease	I20-I25	*
	Angina pectoris	I20	*
	Myocardial infarction	I21	*
	Pulmonary embolism	I26	
	Arrhythmias	I46-I49	*
	Heart failure	I50	*
	Cerebrovascular diseases	I60–I69	*
	Intracerebral haemorrhage	I61	*
	Cerebral infarction	I63	*
	Arteriosclerosis	I70	*
	Deep vein thrombosis	I80-I82	
**X**	**Diseases of the respiratory system**	**J00–J99**	*
	Influenza and Pneumonia	J09–J18	
	Chronic obstructive bronchitis	J43-J44, J47	*
	Asthma	J45-J46	*
**XI**	**Diseases of the digestive system**	**K00–K93**	*
	Appendicitis	K35	*
	Inflammatory bowel disease	K50–K52	*
	Diseases of liver	K70–K77	
	Alcoholic liver disease	K70	
	Pancreatitis	K85	
**XII**	**Diseases of the skin**	**L00-L99**	*
	Infections and eczema	L00-L08, L20-L30	
**XIII**	**Diseases of the musculoskeletal system**	**M00-M99**	*
	Rheumatoid arthritis and related disorders	M05-M06, M08, M13, M30-35, M45	*
	Gout	M10	*
	Osteoarthritis	M15–M19	
	Sciatica	M50-M51	
	Back pain	M54	
	Soft tissue disorders	M60–M79	
**XIV**	**Diseases of the genitourinary system**	**N00-N99**	*
	Renal failure	N17–N19	*
**XV**	**Pregnancy complications**	**O00-O29**	*
	Spontaneous abortion	O03	
	Hypertension in pregnancy	O13–O16	
	Diabetes in pregnancy	O24	
**XVII**	**Circulatory and respiratory symptoms**	R00–R09	
**XVIII**	**Digestive and abdominal symptoms**	R10–R19	
**XIX**	**Injury and poisoning**	**S00-T65**	
	Injury	S00-T35	
	Poisoning	T36-T65	
**XX**	**External causes**	**V01-X84**	
	Road accidents	V01-V99	
	Falls	W00-W19	
	Self-harm	X60-X84	

*Participants with a history of the disease at baseline were excluded from the analysis of neighbourhood characteristics and new-onset disease.

FURTHER DETAILS ABOUT CORRECTION FOR MULTIPLE TESTING

Adjustment for multiple testing in outcome-wide studies is used to avoid false negative findings and several techniques are available for this, including adjustment for false discovery rate (FDR) of 5% and the more conservative Bonferroni correction.[16] However, a policy of not correcting for multiple testing has also been recommended because reducing the type I error (i.e. false positives or "chance findings") increases the type II error (false negatives) for associations that are not null.[17] A further suggestion is to abandon statistical significance (p-value) and instead focus on the point estimate and its confidence intervals considering all the values between the interval's limits reasonably compatible with the data.[18]

In this study, we corrected all step 1 analyses using Bonferroni approach for 79 tests - the number of health outcomes tested in relation to each neighbourhood characteristic. The threshold for statistical significance was 0.0006 yielding significant associations with 12 health outcomes for neighbourhood

education, 16 for neighbourhood income, 20 for neighbourhood unemployment, and 9 for neighbourhood green space. The corresponding numbers were 30, 36, 40 and 29 without Bonferroni correction ($p < 0.05$) and 12, 15, 17 and 6 with a more stringent Bonferroni correction for 4 x 79 tests (threshold for statistical significance 0.00016). For detailed results, see **eTable 6**. All main analyses in steps 2-4 were based on a conventional alpha level ($p < 0.05$) although, in sensitivity analyses of smaller sample sizes, deviation from main results were interpreted by focusing on point estimates and 95% confidence intervals.

In an effort to be as transparent as possible, by providing point estimates, their 95% confidence intervals and precise p-values (4 decimal places) in appendices, we leave opportunity for interested and statistically-minded readers to apply a method of multiple test adjustment of their choice. In appendix 2, we also report all associations of the four neighbourhood characteristics and their changes with 79 health outcomes based on continuous exposure measures to avoid any arbitrary thresholds for categorisation.

SUMMARY OF SUPPLEMENTARY RESULTS

Steps of analysis, number of tests conducted, and number of statistically significant findings are summarised in **eTable 2.**

Proportional hazards assumption: **eTable 3** shows results from tests of the proportionality assumption. Of the 79 associations between neighbourhood education and health outcomes, 74 met the assumption whereas a statistically significant ($p<0.05$) interaction between exposure and length of follow-up was observed for 5 health outcomes. The corresponding figures were 73 and 6 for neighbourhood income, 72 and 7 for neighbourhood unemployment, and 74 and 5 for neighbourhood green space. As the log-log plots in **eFigure 4** indicate, deviation from the proportional hazards assumption was most cases caused by the weakening of the association over time

Characteristics of the study population: Baseline characteristics of the participants for analyses of health outcomes (Sample A) and lifestyle change (Sample B) are shown in **eTable 4**. In **eTable 5** we show comparison of the two participating cohort studies, the population-based HeSSup and the FPS occupational cohort. Supplementary results by step of analysis (table 1) are as follows:

The first analysis step: **eTable 6** shows hazard ratios for the associations between each of the four dichotomised (advantaged vs disadvantaged) neighbourhood characteristics and each of the 79 health outcomes. Hazard ratios are adjusted for age, sex, education and cohort. Tests of interactions between neighbourhood characteristics and cohort on health outcomes suggest that only two associations differed between cohorts after adjusting for multiple testing: The associations of neighbourhood income with mental disorders (p interaction 0.0002) and substance abuse (p interaction 0.0001) were stronger in the population-based HeSSup study than in the FPS occupational cohort (**eTable 7**).

The second analysis step: In **eTable 8**, we report hazard ratios for the associations of change in each neighbourhood characteristic and those 30 health outcomes that were associated with neighbourhood characteristics after Bonferroni correction. These analyses were stratified by baseline level of the neighbourhood characteristic and hazard ratios are adjusted for age, sex, education and cohort. **eTable 9** shows a comparison of prevalent and new-onset disease rates between participants who moved and did not move residential address during the observation period. Of all 79 health outcomes before follow-up, the prevalence was higher among movers than non-movers for 23 and lower in four of these endpoints. The corresponding numbers for the rate of new-onset disease during the follow-up were 29 and three. Results from stratified analyses for non-movers and movers are provided in **eTables 10** and **11**. The following 14 health outcomes were associated with both favourable and unfavourable changes in neighbourhood characteristics either in the total cohort or those with stable residential address or both: death, endocrine diseases, diabetes, mental and behavioural disorders, disorders due to substance abuse, psychotic disorders, diseases of the nervous system, stroke, chronic obstructive bronchitis, diseases of the digestive system, digestive and abdominal symptoms, diseases of the skin, osteoarthritis and soft tissue disorders. Of these, robust associations with substance abuse disorders were seen in movers only.

The third step of analysis: **eTable 12** shows baseline characteristics of the study population in analysis of 8 non-randomised neighbourhood modifications in participants who did not move. While differences in age and sex between the groups were small, those exposed to favourable neighbourhood modifications or being exclusively based in advantaged neighbourhoods tend to have higher education than those exposed to unfavourable neighbourhood modification or remaining in disadvantaged neighbourhoods. **eFigure 5** shows that the main results on four health outcomes (diabetes, stroke, diseases of the skin and osteoarthritis), adjusted for age, sex, education and cohort, remained after excluding those who did not

have stable employment during the observation period. Results for men and women, participants with more than one lifestyle risk factors and those living in urban areas are provided in **eTables 13** and **14**. The results show that the associations were directionally consistent with the main findings although statistical significance was not always reached perhaps due to smaller sample size (for analyses of subgroups with no risk factors or rural residential address, statistical power was diminished). The results were also robust to multivariable adjustments (**eTable 15**).

eTable 16 shows results from a sensitivity analysis using 750m x 750m instead of 250m x 250m spatial units to define neighbourhood characteristics. Averaging over larger areas means lower data resolution; that is, for areas that have heterogenous characteristics, an average over a wider area will result in a failure to capture with precision the local conditions that might influence health. Associations with health are therefore expected to be weaker for the larger areas. Confirming this, we found an attenuation in the magnitude of the effect estimates when using a cruder 750m x 750m spatial unit**.**

The fourth step of analysis: **eTable 17** shows that the main findings on parallel changes in neighbourhood characteristics and lifestyle were little changed in analyses of participants with stable employment. This was also the case when analyses were restricted to participants who lived in urban areas (**eTable 18**).

The fifth step of analysis: These results are available as an excel file in appendix 2.

Supplementary analyses on skin diseases: In detailed analysis of diagnoses, **eTable 19** shows that the association between change in neighbourhood unemployment and risk of skin diseases may be driven by health conditions related to hygiene.

eTable 2. Steps of analysis, number of tests conducted, and statistically significant findings

**STEP 1: LEVEL OF NEIGHBOURHOOD CHARACTERISTICS** Analysis of associations between level of neighbourhood characteristics and 79 health outcomes	**Number of tests**	**Number of significant associations**	**Results reported**
Threshold for statistical significance after Bonferroni correction for multiple testing 0.0006 [p < 0.05]			
Education	79	12 [30]	Table 1,
Income	79	16 [36]	Appendix 1,
Unemployment	79	20 [40]	pp. 13-17
Green space	79	9 [29]	
Based on step 1 analyses, select health outcomes significantly (Bonferroni) associated with one of more neighbourhood characteristics for further analysis (in total 30 health outcomes).			
**STEP 2: CHANGE IN NEIGHBOURHOOD CHARACTERISTICS**			
**Analysis of associations of favourable & unfavourable change in neighbourhood characteristic and 30 health outcomes**			
Threshold for statistical significance p < 0.05)			
Education			
Favourable change	30	8	Figures 3 and
Unfavourable change	30	12	4, Appendix 1,
Income			pp. 18-30
Favourable change	30	11	
Unfavourable change	30	17	
Unemployment			
Favourable change	30	16	
Unfavourable change	30	16	
Green space			
Favourable change	30	10	
Unfavourable change	30	6	
Based on main and sensitivity analyses in step 2, neighbourhood-health outcome pairs in which the associations for both favourable and unfavourable change are statistically significant (p < 0.05) were selected for further analyses, a total of 14 health outcomes.			
**STEP 3: 'NATURAL EXPERIMENT' ON NEIGHBOURHOOD MODIFICATION**			
**Analysis of associations between modification (favourable and unfavourable) of neighbourhood characteristics and 14 health outcomes in a subgroup of participants who were residentially stable**			
Threshold for statistical significance p < 0.05			
Education	5	1	Figure 5,
Income	7	1	Appendix 1,
Unemployment	9	2	pp. 31-39
Green space	2	1	
**STEP 4: LIFESTYLE CHANGES AS OUTCOME**			
**Analysis of associations of upward and downward change in each neighbourhood characteristics and change in 4 lifestyle factors people who were residentially stable, including sensitivity tests**			
Threshold for statistical significance p < 0.05			
Education			
Favourable change	5	1	Table 2,
Unfavourable change	5	2	Appendix 1,
Income			pp. 40-41
Favourable change	5	1	
Unfavourable change	5	0	
Unemployment			
Favourable change	5	3	
Unfavourable change	5	1	
Green space			
Favourable change	5	2	
Unfavourable change	5	2	
**STEP 5: POST-HOC ANALYSES**			
**Analysis using alternative definitions of neighbourhood characteristics**			
Standardised z-score for the ***level*** *of neighbourbood characteristics. Threshold for statistical significance after Bonferroni correction for multiple testing 0.0006 [p < 0.05 in brackets]*			
Education	79	19 [44]	Appendix 2
Income	79	25 [48]	
Unemployment	79	28 [51]	
Green space	79	12 [35]	
Standardised z-score for ***change*** *in neighbourbood characteristics. Threshold for statistical significance after Bonferroni correction for multiple testing 0.0006 [p < 0.05 in brackets]*			
Education	79	0 [5]	
Income	79	5 [13]	
Unemployment	79	0 [8]	
Green space	79	0 [9]	
Number *of favourable/adverse neighbourhood characteristics (0-4). Threshold for statistical significance after Bonferroni correction for multiple testing 0.0006 [p < 0.05 in brackets]*	79	15 [37]	

eTable 3. Test of proportional hazards assumption for associations between 4 neighbourhood characteristics and 79 health outcomes (χ^2 and p-value for the 'exposure x log(follow-up period)' term)

	Education		Income		Unemployment		Green space	
Health outcome	ChiSq (DF=1)	ProbChiSq	ChiSq (DF=1)	ProbChiSq	ChiSq (DF=1)	ProbChiSq	ChiSq (DF=1)	ProbChiSq
Death	0.10	0.7516	1.91	0.1671	0.11	0.7367	0.00	0.9713
Infections	1.13	0.2885	0.46	0.4970	0.65	0.4193	0.03	0.8696
Bacterial infections	0.46	0.4989	0.14	0.7092	0.59	0.4415	0.47	0.4941
Viral infections	0.02	0.8960	0.16	0.6887	0.06	0.8073	5.03	0.0249
Cancer	0.05	0.8148	1.36	0.2432	0.06	0.8027	0.13	0.7236
Colorectal cancer	0.95	0.3302	1.68	0.1944	2.49	0.1146	0.58	0.4465
Lung cancer	0.06	0.8049	0.24	0.6232	0.55	0.4570	3.45	0.0632
Melanoma	0.07	0.7968	0.35	0.5537	0.08	0.7728	0.02	0.8773
Breast cancer	2.19	0.1392	2.23	0.1354	0.77	0.3788	0.00	0.9532
Prostate cancer	1.19	0.2743	0.10	0.7519	1.06	0.3041	1.12	0.2893
Kidney cancer	0.41	0.5195	0.01	0.9353	0.07	0.7867	2.53	0.1116
Brain cancer	0.05	0.8284	2.69	0.1007	0.84	0.3582	4.75	0.0293
Leukaemia, lymphoma	1.14	0.2847	0.04	0.8452	0.38	0.5350	2.89	0.0892
Diseases of the blood	2.53	0.1114	2.20	0.1378	3.54	0.0599	0.19	0.6613
Anemia	2.18	0.1397	3.81	0.0508	1.32	0.2504	3.15	0.0760
Endocrine diseases	3.31	0.0687	0.01	0.9229	0.61	0.4366	0.26	0.6074
Diabetes	3.36	0.0668	0.21	0.6482	0.79	0.3749	0.00	0.9828
Obesity	0.02	0.8983	0.01	0.9300	0.76	0.3832	1.04	0.3075
Mental and behavioural disorders	0.49	0.4822	0.63	0.4270	0.76	0.3833	0.99	0.3188
Dementia	2.19	0.1388	1.85	0.1742	0.16	0.6847	0.00	0.9958
Disorders due to substance abuse	11.22	0.0008	8.05	0.0045	7.35	0.0067	0.55	0.4568
Psychotic disorders	0.64	0.4241	0.08	0.7735	0.00	0.9500	1.35	0.2456
Mood disorders	0.66	0.4182	3.46	0.0629	0.72	0.3948	0.50	0.4803
Neurotic disorders	0.02	0.8912	1.15	0.2828	1.69	0.1938	0.34	0.5624
Diseases of the nervous system	2.86	0.0911	0.98	0.3213	2.27	0.1320	0.08	0.7837
Parkinson disease	1.54	0.2140	0.05	0.8214	1.40	0.2373	0.14	0.7119
Multiple sclerosis	0.90	0.3434	0.55	0.4574	0.84	0.3591	3.06	0.0800
Epilepsy	0.00	0.9632	0.77	0.3797	0.12	0.7272	2.82	0.0933
Headaches	0.39	0.5341	0.06	0.8052	5.85	0.0156	0.16	0.6877
TIA	0.02	0.8811	0.01	0.9278	0.12	0.7243	0.46	0.4971
Sleep disorders	1.36	0.2440	3.82	0.0508	1.27	0.2607	2.26	0.1324
Diseases of the eye	1.33	0.2485	0.01	0.9092	0.03	0.8537	0.92	0.3382
Diseases of the ear	1.34	0.2464	1.39	0.2381	0.52	0.4726	0.03	0.8718
Diseases of the circulatory system	0.32	0.5691	0.30	0.5851	0.43	0.5126	0.08	0.7799
Hypertension	1.78	0.1828	0.00	0.9704	4.03	0.0448	3.99	0.0458
Ischemic heart diseases	2.07	0.1505	1.06	0.3031	0.13	0.7165	0.99	0.3206
Angina pectoris	0.00	0.9523	0.12	0.7337	0.18	0.6714	0.47	0.4911
Myocardial infarction	0.25	0.6204	0.00	0.9793	0.22	0.6372	0.92	0.3362
Pulmonary embolism	0.68	0.4113	0.28	0.5967	0.17	0.6813	0.07	0.7884
Arrhythmias	0.06	0.8114	0.28	0.5989	0.00	0.9470	1.06	0.3037
Heart failure	0.61	0.4341	0.09	0.7674	0.12	0.7290	1.22	0.2699
Cerebrovascular diseases	0.06	0.8068	1.11	0.2929	0.21	0.6460	0.03	0.8565
Stroke	0.36	0.5510	0.63	0.4259	0.33	0.5656	0.32	0.5706
Intracerebral haemorrhage	0.00	0.9777	0.00	0.9657	0.65	0.4200	0.03	0.8553
Cerebral infarction	2.48	0.1154	0.54	0.4625	0.04	0.8324	0.31	0.5773
Arteriosclerosis	0.51	0.4759	1.02	0.3134	0.08	0.7766	2.55	0.1102
Deep vein thrombosis	5.87	0.0154	1.37	0.2419	2.34	0.1260	0.33	0.5673
Diseases of the respiratory system	0.00	0.9980	0.03	0.8568	1.32	0.2501	0.09	0.7635
Influenza and pneumonia	2.49	0.1144	1.20	0.2739	0.09	0.7580	0.09	0.7623
Chronic obstructive bronchitis	0.04	0.8479	0.98	0.3233	3.24	0.0718	0.02	0.8793
Asthma	1.29	0.2562	1.42	0.2331	13.33	0.0003	0.11	0.7393
Diseases of the digestive system	0.60	0.4386	0.01	0.9253	0.05	0.8215	1.95	0.1623
Appendicitis	2.77	0.0962	8.31	0.0039	2.65	0.1036	0.02	0.8799
Inflammatory bowel disease	0.55	0.4580	1.47	0.2248	0.03	0.8659	0.00	0.9898
Diseases of liver	3.94	0.0472	3.44	0.0637	0.48	0.4871	0.47	0.4941
Alcoholic liver disease	1.67	0.1962	2.83	0.0925	1.67	0.1958	1.12	0.2895
Pancreatitis	1.69	0.1941	0.07	0.7982	1.25	0.2630	1.19	0.2746
Diseases of the skin	0.90	0.3431	0.00	0.9930	0.63	0.4268	1.92	0.1660
Infections and excema	0.00	0.9509	0.51	0.4755	0.20	0.6565	0.43	0.5119
Diseases of the musculoskeletal system	2.04	0.1532	2.34	0.1257	0.00	0.9616	0.11	0.7434
Rheumatoid arthritis and related disord	0.10	0.7475	3.53	0.0603	0.46	0.4970	0.97	0.3256
Gout	2.18	0.1394	2.05	0.1519	0.89	0.3445	1.33	0.2479
Osteoarthritis	0.49	0.4818	0.13	0.7225	0.38	0.5375	0.01	0.9275
Sciatica	0.00	0.9904	0.08	0.7827	0.08	0.7797	1.46	0.2266
Back pain	0.53	0.4648	0.09	0.7686	0.03	0.8640	0.05	0.8319
Soft tissue disorders	9.38	0.0022	5.53	0.0186	2.65	0.1034	0.77	0.3791
Diseases of the genitourinary system	0.07	0.7934	0.80	0.3720	0.83	0.3618	2.39	0.1225
Renal failure	0.73	0.3920	1.97	0.1601	0.04	0.8480	1.23	0.2680
Pregnancy complications	0.02	0.8984	27.33	<.0001	4.62	0.0315	23.60	<.0001
Spontaneous abortion	1.01	0.3146	3.55	0.0597	3.42	0.0646	14.13	0.0002
Hypertension in pregnancy	1.08	0.2984	9.15	0.0025	0.01	0.9294	4.45	0.0349
Diabetes in pregnancy	0.02	0.8942	8.75	0.0031	5.35	0.0207	1.85	0.1733
Circulatory and respiratory symptoms	0.00	0.9620	0.31	0.5768	1.77	0.1833	1.43	0.2325
Digestive and abdominal symptoms	0.00	0.9825	0.05	0.8310	0.87	0.3511	0.79	0.3753
Injury	0.96	0.3283	0.04	0.8332	2.16	0.1415	0.16	0.6905
Poisoning	1.23	0.2669	5.53	0.0187	2.96	0.0856	0.65	0.4206
Road accidents	4.74	0.0294	0.57	0.4500	1.85	0.1743	0.43	0.5132
Falls	0.16	0.6848	0.19	0.6619	2.14	0.1437	0.13	0.7205
Self-harm	0.85	0.3560	5.87	0.0154	0.00	0.9503	2.91	0.0881

*For clarity, statistically significant (p<0.05) interactions are highlighted

eFigure 4. Log-log plots of non-proportional neighbourhood characteristic -health outcome associations

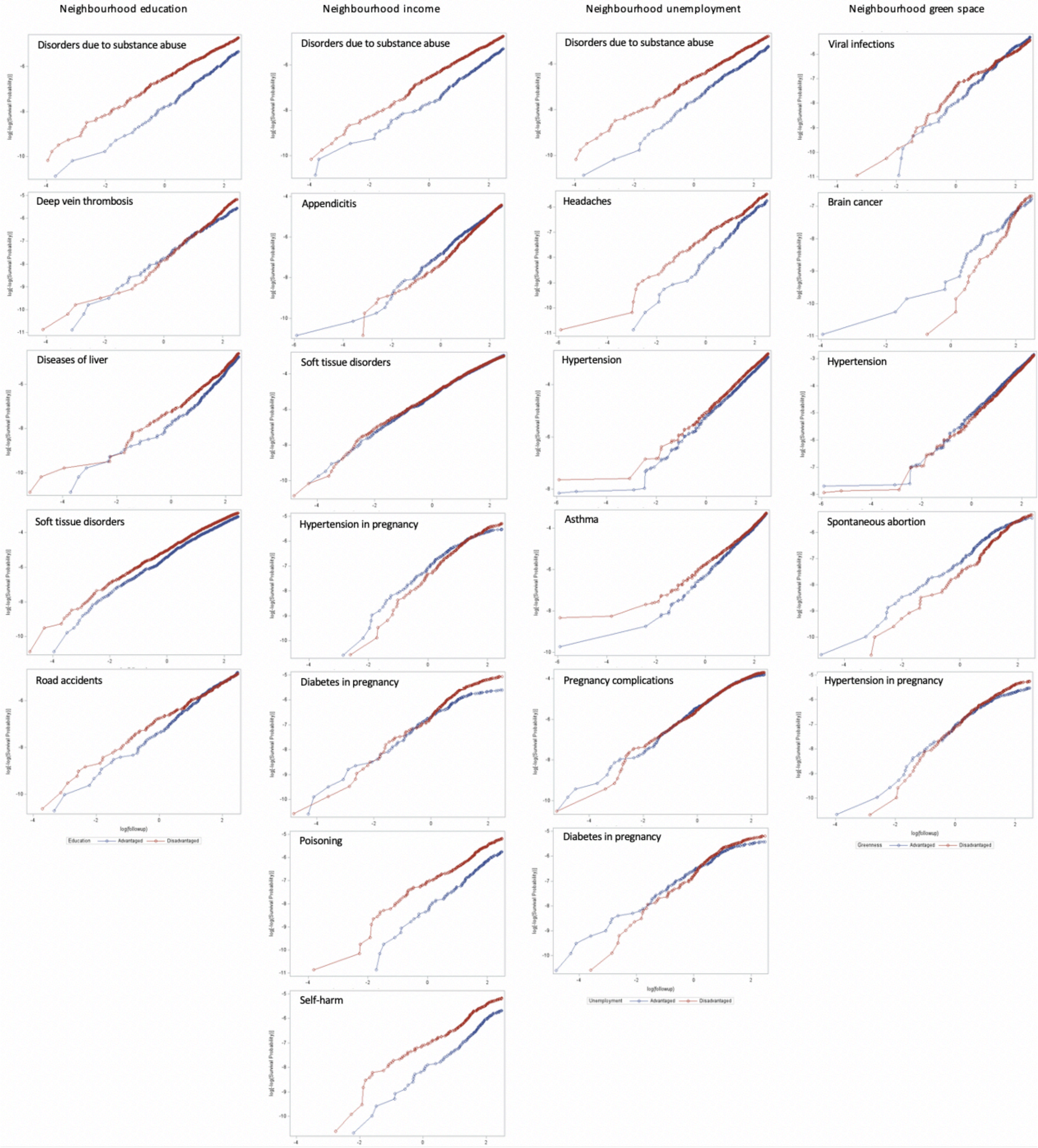

eTable 4. Characteristics of the study population by sample

	Sample 1*	Sample 2*	
**Characteristics**		**Baseline**	**Follow-up**
Number of participants	114786	82152	82152
Cohort, N (%)			
Finnish Public Sector study	91131 (79.4)	63705 (77.6)	63705 (77.6)
HeSSup	23655 (20.6)	18447 (22.4)	18447 (22.4)
Number of observations		147370	147370
Years for baseline and follow-up survey			
1998 and 2003 or 2000 and 2004	--	53993 (65.7)	53993 (65.7)
2004 and 2008	--	13905 (17.0)	13905 (17.0)
2008 and 2012	--	14254 (17.4)	14254 (17.4)
Mean age (SD)	44.4 (11.0)	43.1 (10.8)	47.2 (10.7)
Median age (IQR)	45.0 (36.0 to 54.0)	44.0 (35.0 to 52.0)	48.0 (39.0 to 56.0)
Sex , N (%)			
Men	27774 (24.2)	18927 (23.0)	18927 (23.0)
Women	87012 (75.8)	63225 (77.0)	63225 (77.0)
Education, N (%)			
Primary	13696 (12.0)	11257 (13.7)	11257 (13.7)
Secondary	42724 (37.3)	30822 (37.6)	30822 (37.6)
Tertiary	58201 (50.8)	39881 (48.6)	39881 (48.6)
Neighborhood chartacteristics, Median (IQR)			
Education, yrs†	0.26 (-0.36 to 0.89)	0.27 (-0.33 to 0.88)	0.25 (-0.36 to 0.88)
Household Income†	0.28 (-0.42 to 1.04)	0.26 (-0.48 to 1.11)	0.33 (-0.36 to 1.06)
Unemployment rate†	0.33 (-0.25 to 0.76)	0.38 (-0.15 to 0.80)	0.34 (-0.23 to 0.77)
Greenness index	0.57 (0.47 to 0.65)	0.57 (0.47 to 0.64)	0.58 (0.48 to 0.65)
Population density†	145 (65 to 319)	152 (66 to 331)	138 (61 to 302)
Lifestyle factors			
Smoking, N (%)			
Never-smoker	--	48763 (60.6)	50426 (62.4)
Ex-smoker	--	17278 (21.5)	17678 (21.9)
Current smoker	--	14475 (18.0)	12655 (15.7)
Risky drinking, N (%)			
No	--	64061 (78.5)	64529 (79.0)
Yes	--	17585 (21.5)	17123 (21.0)
Physical inactivity, N (%)			
No	--	65199 (80.0)	64079 (78.5)
Yes	--	16288 (20.0)	17581 (21.5)
Body mass index, N (%)			
<25 (normal weight)	--	45417 (56.3)	40844 (50.8)
25 - 29 (overweight)	--	25663 (31.8)	27753 (34.5)
≥30 (obesity)	--	9616 (11.9)	11810 (14.7)
Number of risks, N (%)			
0-1	--	68341 (83.2)	67763 (82.5)
2-4	--	13811 (16.8)	14389 (17.5)
Additional covariates			
Married or cohabiting, N (%)			
Yes	84747 (74.6)	--	--
No	28933 (25.4)	--	--
In employment during 5-y exposure, N (%)			
All the time	90000 (78.4)	--	67517 (82.2)
Part of the time or not at all	24786 (21.6)	--	14635 (17.8)
Moving of residence, N (%)			
Non-mover	71805 (62.6)	--	57178 (69.5)
Mover	42981 (37.4)	--	25131 (30.5)
Place of residence, N (%)			
Urban	96556 (84.6)	69474 (84.4)	68749 (83.5)
Rural	17573 (15.4)	12830 (15.6)	13556 (16.5)
Type of residence ₮ , N (%)			
One-family house	31791 (38.8)	--	--
Terraced	21967 (26.8)	--	--
Apartment	28104 (34.3)	--	--
Rooms in the residence ₮ , N (%)			
1-3	41210 (50.3)	--	--
>3	40724 (49.7)	--	--
Floor area of residence ₮ , N (%)			
<87 m2	40416 (49.3)	--	--
≥87 m2	41548 (50.7)	--	--

* Sample 1 is for analysis of morbidity and mortality and Sample 2 is for analysis of lifestyle risk factors.
Figures are for participants in Sample 1 and for observations from repeated measurements for Sample 2
†Z-score standardized to the national mean. Higher values indicate increasing advantage.
₮ Data available for FPS only

eTable 5. Characteristics of the study population by cohort

	Cohort	
**Characteristics**	**FPS**	**HeSSup**
Number of participants	91131	23655
Mean age (SD)	45.4 (10.5)	40.7 (11.9)
Median age (IQR)	46.0 (37.0 to 54.0)	39.0 (29.0 to 50.0)
Sex , N (%)		
Men	18140 (19.9)	9634 (40.7)
Women	72991 (80.1)	14021 (59.3)
Education, N (%)		
Primary	8246 (9.1)	5450 (23.2)
Secondary	30492 (33.5)	12232 (52.1)
Tertiary	52392 (57.5)	5809 (24.7)
Neighborhood chartacteristics, median (IQR)		
Education, yrs†	0.31 (-0.28 to 0.94)	0.02 (-0.60 to 0.68)
Household Income†	0.34 (-0.38 to 1.08)	0.06 (-0.58 to 0.84)
Unemployment rate†	0.34 (-0.23 to 0.77)	0.26 (-0.38 to 0.72)
Greenness index	0.57 (0.47 to 0.64)	0.59 (-0.48 to 0.67)
Population density	152 (73 to 324)	116 (34 to 297)
Married or cohabiting, N (%)		
Yes	67885 (75.3)	16862 (71.6)
No	22240 (24.7)	6693 (28.4)
In employment during 5-y exposure, N (%)		
All the time	75800 (83.2)	14200 (60.0)
Part of the time or not at all	15331 (16.8)	9455 (40.0)
Moving of residence, N (%)		
Non-mover	58448 (64.1)	13357 (56.5)
Mover	32683 (35.9)	10298 (43.5)
Place of residence, N (%)		
Urban	79821 (87.9)	16735 (71.9)
Rural	11028 (12.1)	6545 (28.1)

†Z-score standardized to the national mean. Higher values indicate higher advantage.

eTable 6. Associations between level of neighbourhood characteristics (median split) and 79 health outcomes after adjustment for age, sex, education and cohort

			High vs low neighbourhood education			
**Health outcome**	**Total**	**Cases**	**HazardRatio**	**LowerCI**	**UpperCI**	**ProbChiSq**
Death	106422	3189	0.86	0.80	0.92	<.0001
Infections	104446	2796	0.97	0.90	1.05	0.4265
Bacterial infections	106422	2605	0.98	0.91	1.06	0.666
Viral infections	106422	404	0.83	0.68	1.02	0.0757
Cancer	104421	5125	1.02	0.96	1.08	0.5772
Colorectal cancer	106338	485	1.03	0.86	1.24	0.739
Lung cancer	106400	268	0.76	0.59	0.98	0.0333
Melanoma	106246	1225	1.03	0.92	1.15	0.6319
Breast cancer	80019	2155	1.11	1.02	1.21	0.0162
Prostate cancer	25283	486	1.11	0.92	1.33	0.2755
Kidney cancer	106390	125	1.01	0.70	1.44	0.9773
Brain cancer	106395	108	1.05	0.71	1.54	0.8253
Leukaemia, lymphoma	106197	563	1.07	0.90	1.27	0.4373
Diseases of the blood	106036	591	0.95	0.80	1.12	0.5222
Anemia	106422	351	0.72	0.58	0.89	0.003
Endocrine diseases	103714	6449	0.82	0.78	0.86	<.0001
Diabetes	104583	5237	0.80	0.76	0.85	<.0001
Obesity	106422	390	0.61	0.50	0.76	<.0001
Mental and behavioural disorders	104735	1813	0.85	0.77	0.93	0.0008
Dementia	106382	498	0.82	0.69	0.99	0.0392
Disorders due to substance abuse	106422	636	0.64	0.54	0.75	<.0001
Psychotic disorders	105521	665	0.88	0.75	1.03	0.1109
Mood disorders	105602	891	0.94	0.82	1.08	0.3797
Neurotic disorders	106063	294	0.88	0.70	1.12	0.3002
Diseases of the nervous system	102887	4980	0.93	0.87	0.98	0.0071
Parkinson disease	106343	238	0.96	0.74	1.24	0.7419
Multiple sclerosis	106269	222	1.09	0.83	1.42	0.5426
Epilepsy	105333	511	0.90	0.75	1.07	0.2352
Headaches	106422	342	0.98	0.79	1.22	0.8792
TIA	106254	725	0.99	0.85	1.15	0.8622
Sleep disorders	106422	2632	0.82	0.76	0.89	<.0001
Diseases of the eye	104888	5150	0.99	0.94	1.05	0.7945
Diseases of the ear	105532	858	0.93	0.81	1.07	0.3235
Diseases of the circulatory system	99540	7828	0.94	0.90	0.98	0.0057
Hypertension	98487	4633	0.95	0.90	1.01	0.1224
Ischemic heart diseases	105317	2378	0.95	0.88	1.03	0.2273
Angina pectoris	105689	954	0.97	0.85	1.10	0.6158
Myocardial infarction	106149	811	0.82	0.71	0.95	0.0071
Pulmonary embolism	106422	402	0.89	0.73	1.09	0.2602
Arrhythmias	105502	3808	0.95	0.89	1.01	0.094
Heart failure	106255	504	0.84	0.70	1.00	0.0529
Cerebrovascular diseases	106021	1291	1.00	0.89	1.12	0.9783
Stroke	106115	1086	0.98	0.87	1.11	0.726
Intracerebral haemorrhage	106380	183	0.78	0.58	1.06	0.1091
Cerebral infarction	106248	761	1.00	0.86	1.16	0.978
Arteriosclerosis	106334	209	0.85	0.64	1.14	0.2757
Deep vein thrombosis	106422	432	0.74	0.61	0.90	0.003
Diseases of the respiratory system	99227	5442	0.89	0.85	0.94	<.0001
Influenza and Pneumonia	106422	2205	0.90	0.83	0.98	0.0173
Chronic obstructive bronchitis	106327	454	0.80	0.66	0.97	0.0199
Asthma	101314	3015	0.91	0.85	0.98	0.0127
Diseases of the digestive system	98362	9158	0.89	0.85	0.93	<.0001
Appendicitis	106422	1069	0.91	0.80	1.03	0.1263
Inflammatory bowel disease	105435	767	0.90	0.78	1.04	0.1563
Diseases of liver	106422	804	0.93	0.81	1.08	0.3412
Alcoholic liver disease	106422	190	0.83	0.62	1.12	0.2146
Pancreatitis	106422	280	0.78	0.61	0.99	0.0424
Diseases of the skin	105339	934	0.87	0.76	0.99	0.0325
Infections and excema	106422	487	0.84	0.70	1.01	0.0699
Diseases of the musculoskeletal system	93454	12406	0.90	0.86	0.93	<.0001
Rheumatoid arthritis and related disorders	104515	1887	0.90	0.82	0.98	0.0182
Gout	106288	219	0.81	0.61	1.06	0.1228
Osteoarthritis	106422	5513	0.81	0.77	0.85	<.0001
Sciatica	106422	1380	0.96	0.86	1.07	0.4266
Back pain	106422	563	1.08	0.91	1.27	0.3976
Soft tissue disorders	106422	4699	0.87	0.82	0.92	<.0001
Diseases of the genitourinary system	96125	8793	0.95	0.91	0.99	0.0122
Renal failure	106350	306	0.81	0.64	1.02	0.0786
Pregnancy complications	76151	1677	1.06	0.96	1.17	0.2408
Spontaneous abortion	81056	339	1.00	0.80	1.24	0.9676
Hypertension in pregnancy	81056	343	1.19	0.96	1.48	0.1084
Diabetes in pregnancy	81056	386	1.01	0.83	1.24	0.9141
Circulatory and respiratory symptoms	106422	1581	0.87	0.78	0.96	0.006
Digestive and abdominal symptoms	106422	1969	0.84	0.76	0.92	0.0001
Injury	106422	9114	1.00	0.96	1.05	0.9382
Poisoning	106422	421	0.73	0.59	0.89	0.0017
Road accidents	86196	619	1.06	0.91	1.25	0.4531
Falls	86196	3819	0.95	0.89	1.01	0.0929
Self-harm	86196	352	0.78	0.63	0.97	0.0222

Health outcome	Total	Cases	High vs low neighbourhood income HazardRatio	LowerCI	UpperCI	ProbChiSq
Death	102959	3104	0.72	0.67	0.77	<.0001
Infections	101054	2710	0.92	0.85	0.99	0.0214
Bacterial infections	102959	2534	0.95	0.88	1.03	0.223
Viral infections	102959	385	0.76	0.62	0.94	0.0092
Cancer	101007	4947	1.03	0.98	1.09	0.2647
Colorectal cancer	102880	470	1.02	0.85	1.22	0.8488
Lung cancer	102938	259	0.76	0.59	0.98	0.0315
Melanoma	102784	1191	1.06	0.94	1.19	0.3341
Breast cancer	77533	2079	1.08	0.99	1.18	0.0814
Prostate cancer	24330	464	1.12	0.93	1.35	0.2189
Kidney cancer	102927	122	1.01	0.70	1.44	0.9753
Brain cancer	102932	104	1.05	0.71	1.54	0.8179
Leukaemia, lymphoma	102743	545	1.06	0.90	1.26	0.4754
Diseases of the blood	102580	571	0.76	0.64	0.90	0.0012
Anemia	102959	341	0.69	0.55	0.85	0.0007
Endocrine diseases	100336	6272	0.83	0.79	0.88	<.0001
Diabetes	101181	5098	0.81	0.77	0.86	<.0001
Obesity	102959	375	0.73	0.59	0.89	0.0025
Mental and behavioural disorders	101325	1762	0.73	0.66	0.80	<.0001
Dementia	102920	482	0.84	0.70	1.01	0.0597
Disorders due to substance abuse	102959	613	0.61	0.52	0.72	<.0001
Psychotic disorders	102089	654	0.63	0.54	0.74	<.0001
Mood disorders	102162	863	0.75	0.66	0.86	<.0001
Neurotic disorders	102611	283	0.74	0.58	0.95	0.0153
Diseases of the nervous system	99554	4783	0.96	0.90	1.01	0.1276
Parkinson disease	102883	231	1.02	0.79	1.32	0.8779
Multiple sclerosis	102813	208	1.10	0.83	1.45	0.5032
Epilepsy	101898	492	0.73	0.61	0.88	0.0007
Headaches	102959	336	1.03	0.83	1.27	0.8243
TIA	102799	690	1.05	0.90	1.22	0.5447
Sleep disorders	102959	2553	0.84	0.78	0.91	<.0001
Diseases of the eye	101463	5005	0.94	0.89	1.00	0.0435
Diseases of the ear	102100	824	0.92	0.81	1.06	0.2637
Diseases of the circulatory system	96307	7562	0.95	0.90	0.99	0.0146
Hypertension	95283	4488	0.98	0.92	1.03	0.4031
Ischemic heart diseases	101889	2294	0.95	0.87	1.03	0.2067
Angina pectoris	102251	915	1.08	0.94	1.23	0.2723
Myocardial infarction	102695	783	0.96	0.83	1.10	0.5351
Pulmonary embolism	102959	396	0.86	0.71	1.06	0.1512
Arrhythmias	102075	3688	0.94	0.88	1.00	0.0519
Heart failure	102797	490	0.69	0.57	0.82	<.0001
Cerebrovascular diseases	102572	1255	0.77	0.69	0.86	<.0001
Stroke	102664	1059	0.75	0.66	0.84	<.0001
Intracerebral haemorrhage	102918	180	0.54	0.40	0.73	<.0001
Cerebral infarction	102790	741	0.80	0.69	0.92	0.0025
Arteriosclerosis	102874	204	0.61	0.46	0.82	0.0009
Deep vein thrombosis	102959	420	0.86	0.71	1.04	0.1154
Diseases of the respiratory system	95985	5278	0.85	0.81	0.90	<.0001
Influenza and Pneumonia	102959	2140	0.88	0.81	0.96	0.0044
Chronic obstructive bronchitis	102866	445	0.55	0.45	0.67	<.0001
Asthma	98006	2921	0.89	0.83	0.96	0.0024
Diseases of the digestive system	95189	8835	0.97	0.93	1.01	0.1111
Appendicitis	102959	1032	1.01	0.89	1.14	0.9225
Inflammatory bowel disease	101999	744	0.96	0.83	1.11	0.6039
Diseases of liver	102959	783	0.85	0.74	0.98	0.0215
Alcoholic liver disease	102959	185	0.71	0.53	0.95	0.0215
Pancreatitis	102959	267	0.87	0.68	1.11	0.2641
Diseases of the skin	101917	901	0.81	0.71	0.92	0.0014
Infections and excema	102959	476	0.80	0.67	0.96	0.0162
Diseases of the musculoskeletal system	90429	11953	1.02	0.98	1.06	0.3501
Rheumatoid arthritis and related disorders	101126	1815	0.98	0.89	1.07	0.5912
Gout	102827	213	0.90	0.69	1.18	0.4397
Osteoarthritis	102959	5325	0.97	0.92	1.02	0.2477
Sciatica	102959	1337	1.12	1.00	1.25	0.0437
Back pain	102959	534	1.10	0.92	1.30	0.301
Soft tissue disorders	102959	4530	1.05	0.99	1.12	0.0865
Diseases of the genitourinary system	93042	8486	1.04	1.00	1.09	0.0517
Renal failure	102889	300	0.88	0.70	1.10	0.2637
Pregnancy complications	73860	1627	1.07	0.96	1.18	0.2189
Spontaneous abortion	78548	324	0.93	0.74	1.17	0.53
Hypertension in pregnancy	78548	331	1.22	0.98	1.53	0.0764
Diabetes in pregnancy	78548	375	0.85	0.68	1.05	0.124
Circulatory and respiratory symptoms	102959	1526	0.91	0.83	1.01	0.0785
Digestive and abdominal symptoms	102959	1886	0.89	0.81	0.97	0.0096
Injury	102959	8798	1.02	0.98	1.06	0.3708
Poisoning	102959	401	0.61	0.50	0.75	<.0001
Road accidents	83735	604	0.92	0.78	1.08	0.297
Falls	83735	3704	0.96	0.90	1.02	0.2063
Self-harm	83735	340	0.65	0.52	0.80	<.0001

Health outcome	Total	Cases	HazardRatio	LowerCI	UpperCI	ProbChiSq
			Low versus high neighbourhood unemployment			
Death	104391	3136	0.84	0.78	0.90	<.0001
Infections	102458	2742	0.96	0.89	1.03	0.2751
Bacterial infections	104391	2563	0.98	0.91	1.06	0.5798
Viral infections	104391	388	0.90	0.74	1.10	0.2939
Cancer	102415	5016	1.01	0.96	1.07	0.6443
Colorectal cancer	104309	474	1.07	0.89	1.28	0.4754
Lung cancer	104369	261	0.99	0.78	1.27	0.9394
Melanoma	104216	1205	0.99	0.88	1.11	0.8457
Breast cancer	78536	2107	1.08	0.99	1.17	0.0995
Prostate cancer	24745	473	1.09	0.91	1.31	0.3399
Kidney cancer	104359	125	0.77	0.54	1.10	0.1451
Brain cancer	104364	107	0.87	0.59	1.28	0.4807
Leukaemia, lymphoma	104173	555	1.12	0.95	1.33	0.1861
Diseases of the blood	104009	577	0.96	0.81	1.13	0.6182
Anemia	104391	345	0.87	0.70	1.07	0.1871
Endocrine diseases	101737	6340	0.85	0.81	0.90	<.0001
Diabetes	102590	5156	0.84	0.80	0.89	<.0001
Obesity	104391	379	0.69	0.56	0.85	0.0004
Mental and behavioural disorders	102742	1782	0.82	0.75	0.90	<.0001
Dementia	104352	485	0.87	0.73	1.04	0.1285
Disorders due to substance abuse	104391	615	0.70	0.59	0.82	<.0001
Psychotic disorders	103505	659	0.83	0.71	0.97	0.02
Mood disorders	103587	875	0.85	0.74	0.97	0.0169
Neurotic disorders	104041	288	0.87	0.69	1.10	0.2423
Diseases of the nervous system	100935	4862	0.86	0.81	0.91	<.0001
Parkinson disease	104314	235	1.16	0.90	1.51	0.2516
Multiple sclerosis	104240	215	0.96	0.73	1.25	0.7429
Epilepsy	103322	499	0.73	0.61	0.88	0.0007
Headaches	104391	339	0.76	0.61	0.94	0.0115
TIA	104228	700	0.82	0.70	0.95	0.0076
Sleep disorders	104391	2588	0.81	0.75	0.87	<.0001
Diseases of the eye	102875	5056	0.98	0.92	1.03	0.3919
Diseases of the ear	103521	836	0.83	0.72	0.95	0.0074
Diseases of the circulatory system	97644	7670	0.89	0.85	0.93	<.0001
Hypertension	96628	4543	0.89	0.84	0.95	0.0001
Ischemic heart diseases	103307	2331	0.81	0.74	0.88	<.0001
Angina pectoris	103673	930	0.88	0.77	1.00	0.0541
Myocardial infarction	104123	800	0.88	0.76	1.01	0.062
Pulmonary embolism	104391	397	0.90	0.74	1.10	0.3086
Arrhythmias	103498	3745	0.96	0.90	1.03	0.2477
Heart failure	104228	493	0.72	0.60	0.87	0.0005
Cerebrovascular diseases	104000	1265	0.85	0.76	0.96	0.0055
Stroke	104093	1067	0.82	0.73	0.93	0.0019
Intracerebral haemorrhage	104350	181	0.72	0.53	0.96	0.0277
Cerebral infarction	104220	745	0.88	0.76	1.02	0.0867
Arteriosclerosis	104304	205	0.75	0.56	0.99	0.0433
Deep vein thrombosis	104391	425	0.81	0.67	0.99	0.0357
Diseases of the respiratory system	97346	5346	0.90	0.85	0.95	0.0001
Influenza and Pneumonia	104391	2170	0.97	0.89	1.06	0.4945
Chronic obstructive bronchitis	104296	450	0.67	0.56	0.82	<.0001
Asthma	99380	2960	0.95	0.89	1.03	0.1938
Diseases of the digestive system	96489	8958	0.90	0.86	0.94	<.0001
Appendicitis	104391	1046	1.02	0.90	1.15	0.7381
Inflammatory bowel disease	103420	750	0.99	0.86	1.14	0.8676
Diseases of liver	104391	793	0.85	0.73	0.97	0.0193
Alcoholic liver disease	104391	186	0.71	0.53	0.95	0.0226
Pancreatitis	104391	270	0.78	0.61	1.00	0.0473
Diseases of the skin	103338	912	0.73	0.64	0.83	<.0001
Infections and excema	104391	477	0.77	0.64	0.92	0.0041
Diseases of the musculoskeletal system	91687	12144	0.94	0.91	0.98	0.0009
Rheumatoid arthritis and related disorders	102535	1839	0.97	0.89	1.07	0.5807
Gout	104259	216	0.71	0.54	0.93	0.0125
Osteoarthritis	104391	5400	0.87	0.82	0.91	<.0001
Sciatica	104391	1359	0.97	0.87	1.08	0.5803
Back pain	104391	547	0.91	0.77	1.08	0.2907
Soft tissue disorders	104391	4601	0.92	0.87	0.97	0.0045
Diseases of the genitourinary system	94333	8606	0.96	0.92	1.01	0.0934
Renal failure	104319	303	0.77	0.61	0.97	0.0272
Pregnancy complications	74777	1646	1.03	0.93	1.13	0.6023
Spontaneous abortion	79563	330	1.09	0.87	1.35	0.4574
Hypertension in pregnancy	79563	337	1.47	1.19	1.83	0.0005
Diabetes in pregnancy	79563	380	0.93	0.75	1.13	0.4513
Circulatory and respiratory symptoms	104391	1550	0.85	0.77	0.94	0.0015
Digestive and abdominal symptoms	104391	1914	0.82	0.75	0.90	<.0001
Injury	104391	8933	1.00	0.96	1.04	0.9218
Poisoning	104391	407	0.63	0.52	0.78	<.0001
Road accidents	84808	610	1.00	0.85	1.17	0.9484
Falls	84808	3767	0.95	0.90	1.02	0.1523
Self-harm	84808	345	0.71	0.58	0.89	0.0023

High versus low neighbourhood green space

Health outcome	Total	Cases	HazardRatio	LowerCI	UpperCI	ProbChiSq
Death	113834	3396	0.83	0.77	0.89	<.0001
Infections	111705	3011	0.94	0.87	1.01	0.0928
Bacterial infections	113834	2788	0.92	0.85	0.99	0.0281
Viral infections	113834	452	1.11	0.92	1.34	0.2758
Cancer	111726	5469	1.02	0.97	1.08	0.4611
Colorectal cancer	113747	519	1.02	0.85	1.21	0.8457
Lung cancer	113810	289	0.99	0.78	1.25	0.9301
Melanoma	113648	1275	1.04	0.93	1.17	0.4528
Breast cancer	85280	2275	0.99	0.91	1.08	0.8862
Prostate cancer	27369	525	1.09	0.91	1.29	0.3639
Kidney cancer	113800	141	0.90	0.64	1.27	0.5561
Brain cancer	113807	117	1.03	0.71	1.49	0.8951
Leukaemia, lymphoma	113593	604	1.02	0.87	1.21	0.7789
Diseases of the blood	113433	616	0.94	0.80	1.11	0.4624
Anemia	113834	368	0.94	0.76	1.16	0.5649
Endocrine diseases	110933	6885	0.94	0.90	0.99	0.0172
Diabetes	111849	5578	0.93	0.88	0.99	0.0118
Obesity	113834	412	1.00	0.82	1.22	0.9785
Mental and behavioural disorders	112031	1923	0.84	0.77	0.92	0.0002
Dementia	113791	523	0.91	0.76	1.09	0.2955
Disorders due to substance abuse	113834	684	0.91	0.78	1.06	0.2113
Psychotic disorders	112870	710	0.70	0.60	0.81	<.0001
Mood disorders	112956	944	0.74	0.65	0.85	<.0001
Neurotic disorders	113449	318	0.99	0.79	1.24	0.953
Diseases of the nervous system	110022	5385	1.04	0.98	1.10	0.1669
Parkinson disease	113750	260	1.12	0.87	1.43	0.3784
Multiple sclerosis	113665	237	1.21	0.94	1.57	0.1476
Epilepsy	112676	543	0.96	0.81	1.14	0.6182
Headaches	113834	374	0.95	0.78	1.17	0.6576
TIA	113653	788	1.10	0.95	1.27	0.2053
Sleep disorders	113834	2823	1.13	1.05	1.22	0.0013
Diseases of the eye	112206	5446	0.97	0.92	1.03	0.2822
Diseases of the ear	112881	928	0.97	0.85	1.11	0.6729
Diseases of the circulatory system	106403	8362	1.01	0.97	1.06	0.5617
Hypertension	105322	4980	1.02	0.97	1.08	0.4605
Ischemic heart diseases	112639	2540	1.03	0.95	1.11	0.4936
Angina pectoris	113041	1029	1.26	1.11	1.42	0.0004
Myocardial infarction	113539	868	0.97	0.84	1.11	0.644
Pulmonary embolism	113834	437	0.94	0.78	1.14	0.5354
Arrhythmias	112829	4045	1.09	1.03	1.16	0.0057
Heart failure	113655	538	0.99	0.83	1.17	0.8747
Cerebrovascular diseases	113396	1374	0.92	0.82	1.02	0.122
Stroke	113498	1148	0.90	0.80	1.02	0.092
Intracerebral haemorrhage	113786	191	0.79	0.59	1.06	0.1209
Cerebral infarction	113644	807	0.89	0.77	1.03	0.1063
Arteriosclerosis	113740	223	0.72	0.54	0.94	0.0178
Deep vein thrombosis	113834	451	0.76	0.63	0.92	0.0052
Diseases of the respiratory system	106116	5842	0.99	0.94	1.05	0.7451
Influenza and Pneumonia	113834	2362	0.92	0.85	1.00	0.0538
Chronic obstructive bronchitis	113730	485	0.86	0.72	1.04	0.1154
Asthma	108393	3205	1.04	0.97	1.11	0.3334
Diseases of the digestive system	105180	9845	1.04	1.00	1.08	0.0621
Appendicitis	113834	1139	1.04	0.92	1.17	0.5579
Inflammatory bowel disease	112776	821	1.21	1.05	1.39	0.008
Diseases of liver	113834	846	0.88	0.77	1.01	0.073
Alcoholic liver disease	113834	203	0.69	0.52	0.92	0.0111
Pancreatitis	113834	305	1.00	0.80	1.26	0.9906
Diseases of the skin	112685	1004	0.97	0.86	1.10	0.6696
Infections and excema	113834	517	0.84	0.70	1.00	0.0554
Diseases of the musculoskeletal system	99877	13362	1.08	1.05	1.12	<.0001
Rheumatoid arthritis and related disorders	111790	2043	1.15	1.05	1.25	0.0023
Gout	113692	233	0.95	0.73	1.24	0.7002
Osteoarthritis	113834	5935	1.11	1.05	1.16	0.0002
Sciatica	113834	1480	1.09	0.98	1.21	0.1182
Back pain	113834	625	1.28	1.09	1.50	0.0029
Soft tissue disorders	113834	5104	1.14	1.08	1.21	<.0001
Diseases of the genitourinary system	102753	9461	1.10	1.06	1.15	<.0001
Renal failure	113753	333	1.12	0.90	1.39	0.3155
Pregnancy complications	81058	1779	1.04	0.95	1.15	0.3803
Spontaneous abortion	86374	366	1.09	0.89	1.34	0.4174
Hypertension in pregnancy	86374	373	0.86	0.70	1.06	0.1504
Diabetes in pregnancy	86374	413	0.92	0.75	1.12	0.3776
Circulatory and respiratory symptoms	113834	1727	1.06	0.97	1.17	0.2074
Digestive and abdominal symptoms	113834	2144	1.14	1.05	1.25	0.0026
Injury	113834	9800	0.97	0.93	1.01	0.1465
Poisoning	113834	445	0.76	0.63	0.92	0.0055
Road accidents	90764	669	0.88	0.76	1.03	0.1056
Falls	90764	4029	0.95	0.89	1.01	0.0849
Self-harm	90764	365	0.68	0.54	0.84	0.0004

eTable 7. Statistical significance of the 'neighbourhood characteristic x cohort' interaction terms on 79 health outcomes

	P-value for interaction			
**Health outcome**	**Education**	**Income**	**Unemployment**	**Greenness**
Death	0.0177	0.0000	0.0073	0.1308
Infections	0.6393	0.5537	0.0846	0.4745
Bacterial infections	0.9715	0.2577	0.2737	0.4276
Viral infections	0.9888	0.2489	0.4575	0.5666
Cancer	0.2178	0.5068	0.1299	0.9595
Colorectal cancer	0.3106	0.8930	0.8414	0.3319
Lung cancer	0.2184	0.4620	0.9151	0.6997
Melanoma	0.7604	0.7507	0.3224	0.4280
Breast cancer	0.9280	0.3624	0.1357	0.9932
Prostate cancer	0.2260	0.8037	0.6778	0.4711
Kidney cancer	0.0257	0.1541	0.1612	0.2923
Brain cancer	0.8911	0.6202	0.3989	0.2904
Leukaemia, lymphoma	0.2044	0.3074	0.6770	0.0626
Diseases of the blood	0.5455	0.2473	0.4729	0.6327
Anemia	0.1301	0.1796	0.4227	0.5188
Endocrine diseases	0.4946	0.0578	0.3757	0.5820
Diabetes	0.2101	0.0529	0.2674	0.2427
Obesity	0.6103	0.2708	0.9386	0.8056
Mental and behavioural disorders	0.0127	**0.0002**	0.0730	0.2549
Dementia	0.0975	0.1090	0.1830	0.1083
Disorders due to substance abuse	0.1089	**0.0001**	0.0804	0.5875
Psychotic disorders	0.2230	0.1470	0.7912	0.8960
Mood disorders	0.6378	0.0366	0.4275	0.9884
Neurotic disorders	0.8948	0.1568	0.0518	0.6791
Diseases of the nervous system	0.2376	0.0480	0.5700	0.0369
Parkinson disease	0.0736	0.4684	0.0992	0.4518
Multiple sclerosis	0.3196	0.4517	0.5140	0.9888
Epilepsy	0.5149	0.2606	0.5729	0.8993
Headaches	0.2647	0.3407	0.7708	0.0785
TIA	0.6498	0.7423	0.9877	0.8332
Sleep disorders	0.0087	0.6422	0.3228	0.0903
Diseases of the eye	0.6297	0.0115	0.4887	0.0780
Diseases of the ear	0.1546	0.6617	0.8029	0.9360
Diseases of the circulatory system	0.0025	0.0333	0.0109	0.6037
Hypertension	0.0135	0.7577	0.6746	0.4484
Ischemic heart diseases	0.0608	0.1846	0.4068	0.8309
Angina pectoris	0.3018	0.3721	0.2796	0.0133
Myocardial infarction	0.0514	0.2883	0.8983	0.6173
Pulmonary embolism	0.2121	0.0872	0.8494	0.2825
Arrhythmias	0.2386	0.9699	0.4014	0.1321
Heart failure	0.0789	0.9946	0.2275	0.1736
Cerebrovascular diseases	0.0114	0.0102	0.0321	0.2091
Stroke	0.0427	0.0068	0.1071	0.4023
Intracerebral haemorrhage	0.8353	0.5526	0.1433	0.3363
Cerebral infarction	0.0033	0.0043	0.1609	0.8290
Arteriosclerosis	0.6780	0.6315	0.5669	0.1774
Deep vein thrombosis	0.3533	0.6925	0.6066	0.0852
Diseases of the respiratory system	0.2127	0.1595	0.4498	0.6668
Influenza and Pneumonia	0.1491	0.0089	0.0304	0.8425
Chronic obstructive bronchitis	0.1973	0.7079	0.2243	0.7774
Asthma	0.0955	0.3363	0.0094	0.5810
Diseases of the digestive system	0.0234	0.5021	0.0593	0.4585
Appendicitis	0.9758	0.4355	0.1576	0.1557
Inflammatory bowel disease	0.5434	0.9045	0.7691	0.9065
Diseases of liver	0.4117	0.1554	0.0572	0.0092
Alcoholic liver disease	0.7966	0.1452	0.3217	0.0039
Pancreatitis	0.9311	0.4106	0.2241	0.6075
Diseases of the skin	0.1681	0.0451	0.4223	0.4407
Infections and excema	0.3101	0.4803	0.5866	0.4244
Diseases of the musculoskeletal system	0.6935	0.8660	0.1607	0.1666
Rheumatoid arthritis and related disorde	0.0978	0.0126	0.0559	0.8026
Gout	0.2801	0.7824	0.4749	0.8683
Osteoarthritis	0.5259	0.3973	0.2869	0.7744
Sciatica	0.6851	0.5419	0.8579	0.3880
Back pain	0.0583	0.5121	0.0558	0.3998
Soft tissue disorders	0.6829	0.2281	0.2802	0.2812
Diseases of the genitourinary system	0.1916	0.9544	0.6360	0.7673
Renal failure	0.5750	0.3804	0.2461	0.4985
Pregnancy complications	0.5901	0.2732	0.8652	0.0391
Spontaneous abortion	0.5442	0.5784	0.5126	0.5080
Hypertension in pregnancy	0.8687	0.4323	0.6191	0.2981
Diabetes in pregnancy	0.8318	0.5602	0.5245	0.0985
Circulatory and respiratory symptoms	0.3904	0.2400	0.0042	0.4298
Digestive and abdominal symptoms	0.1317	0.5513	0.1136	0.3018
Injury	0.8634	0.4456	0.0678	0.6780
Poisoning	0.7177	0.1033	0.0777	0.1752
Road accidents	.	.	.	.
Falls	.	.	.	.
Self-harm	.	.	.	.

For clarity, statistically significant interaction after Bonferronin correction for multipletesting are in bold ($p < 0.0006$).

eTable 8. Association of favourable (upper panel) and unfavourable (lower panel) change in neighbourhood characteristics with 30 subsequent health outcomes

		**Exposure: Change in neighbourhood characteristic**					
		**Increase in education**					
**ICD-chapter**		Total	Cases	HazardRatio	Lower CI	Upper CI	ProbChiSq
	**Death**	50533	1716	**0.87**	**0.77**	**0.99**	**0.0371**
**IV**	**Endocrine diseases**	49066	3455	**0.84**	**0.77**	**0.92**	**0.0001**
	Diabetes	49508	2850	**0.83**	**0.75**	**0.92**	**0.0002**
	Obesity	50533	230	**0.66**	**0.47**	**0.92**	**0.015**
**V**	**Mental and behavioural di**	49619	960	0.85	0.73	1.00	0.0523
	Disorders due to substance	50533	382	**0.67**	**0.50**	**0.88**	**0.0048**
	Psychotic disorders	50025	344	0.90	0.69	1.17	0.4168
	Mood disorders	50105	467	0.94	0.76	1.17	0.5762
**VI**	**Diseases of the nervous sys**	48718	2548	0.95	0.86	1.05	0.3003
	Sleep disorders	50533	1345	0.89	0.78	1.02	0.0978
**IX**	**Diseases of the circulatory**	47079	3922	0.96	0.89	1.04	0.3077
	Hypertension	46240	2287	0.92	0.83	1.02	0.1324
	Ischemic heart diseases	49934	1209	1.02	0.88	1.18	0.8176
	Angina pectoris	50141	482	1.04	0.82	1.30	0.7689
	Heart failure	50434	277	0.95	0.70	1.31	0.7708
	Cerebrovascular diseases	50317	651	1.12	0.93	1.36	0.2412
	Stroke	50361	561	1.12	0.91	1.38	0.3061
	Intracerebral haemorrhage	50507	97	0.83	0.48	1.42	0.4877
**X**	**Diseases of the respiratory**	47117	2728	0.98	0.89	1.07	0.6337
	Chronic obstructive bronch	50473	259	0.85	0.61	1.18	0.324
**XI**	**Diseases of the digestive sy**	46551	4500	**0.89**	**0.83**	**0.96**	**0.0019**
**XII**	**Diseases of the skin**	50016	479	0.92	0.74	1.15	0.484
**XIII**	**Diseases of the musculoske**	43845	6216	0.95	0.89	1.01	0.0758
	Osteoarthritis	50533	2953	**0.85**	**0.78**	**0.94**	**0.0012**
	Soft tissue disorders	50533	2473	0.95	0.86	1.05	0.3267
**XIV**	**Diseases of the genitourina**	45479	4297	1.01	0.94	1.09	0.7337
**XV**	**Pregnancy complications: I**	38357	156	0.92	0.66	1.28	0.6135
**XVIII**	Digestive and abdominal sy	50533	1048	0.87	0.74	1.01	0.0585
**XIX**	**Poisoning**	50533	238	**0.63**	**0.44**	**0.89**	**0.008**
**XX**	**Self-harm**	39145	192	0.70	0.48	1.00	0.0513
		**Decrease in education**					
	**Death**	51116	1408	1.06	0.94	1.20	0.3232
**IV**	**Endocrine diseases**	49946	2822	**1.15**	**1.06**	**1.25**	**0.0011**
	Diabetes	50342	2261	**1.15**	**1.04**	**1.26**	**0.0042**
	Obesity	51116	142	1.37	0.96	1.96	0.0808
**V**	**Mental and behavioural di**	50430	777	1.08	0.92	1.26	0.3534
	Disorders due to substance	51116	230	**1.33**	**1.01**	**1.76**	**0.0458**
	Psychotic disorders	50756	292	1.22	0.95	1.57	0.1179
	Mood disorders	50765	393	0.94	0.75	1.19	0.622
**VI**	**Diseases of the nervous sys**	49509	2273	1.06	0.97	1.17	0.2198
	Sleep disorders	51116	1210	**1.21**	**1.07**	**1.38**	**0.0032**
**IX**	**Diseases of the circulatory**	47882	3654	1.03	0.95	1.11	0.4858
	Hypertension	47630	2242	1.01	0.92	1.11	0.8577
	Ischemic heart diseases	50635	1114	1.07	0.94	1.23	0.3159
	Angina pectoris	50789	451	1.09	0.88	1.35	0.4127
	Heart failure	51051	216	1.10	0.81	1.50	0.5304
	Cerebrovascular diseases	50946	594	1.03	0.85	1.24	0.7965
	Stroke	50994	488	0.98	0.80	1.21	0.8692
	Intracerebral haemorrhage	51105	80	1.25	0.76	2.05	0.3789
**X**	**Diseases of the respiratory**	47791	2465	**1.18**	**1.08**	**1.29**	**0.0003**
	Chronic obstructive bronch	51083	188	1.18	0.86	1.63	0.3045
**XI**	**Diseases of the digestive sy**	47374	4289	**1.09**	**1.02**	**1.17**	**0.0106**
**XII**	**Diseases of the skin**	50614	419	1.20	0.96	1.48	0.1034
**XIII**	**Diseases of the musculoske**	45270	5782	**1.14**	**1.08**	**1.21**	**<.0001**
	Osteoarthritis	51116	2427	**1.18**	**1.07**	**1.29**	**0.0005**
	Soft tissue disorders	51116	2083	**1.20**	**1.09**	**1.32**	**0.0003**
**XIV**	**Diseases of the genitourina**	46254	4180	**1.08**	**1.00**	**1.16**	**0.0382**
**XV**	**Pregnancy complications: I**	39040	147	**0.60**	**0.41**	**0.88**	**0.0089**
**XVIII**	Digestive and abdominal sy	51116	847	**1.16**	**1.00**	**1.35**	**0.0496**
**XIX**	**Poisoning**	51116	168	1.12	0.80	1.57	0.5067
**XX**	**Self-harm**	43149	154	1.12	0.79	1.59	0.5305

ICD-chapter		Exposure: Change in neighbourhood characteristic					
		**Increase in income**					
**ICD-chapter**		Total	Cases	HazardRatio	Lower CI	Upper CI	ProbChiSq
	**Death**	48472	1606	**0.66**	**0.56**	**0.78**	**<.0001**
**IV**	**Endocrine diseases**	47162	3070	**0.79**	**0.71**	**0.87**	**<.0001**
	Diabetes	47556	2511	**0.75**	**0.67**	**0.85**	**<.0001**
	Obesity	48472	194	**0.61**	**0.41**	**0.91**	**0.0146**
**V**	**Mental and behavioural disorders**	47568	956	**0.76**	**0.64**	**0.90**	**0.0019**
	Disorders due to substance abuse	48472	328	**0.57**	**0.40**	**0.79**	**0.001**
	Psychotic disorders	47949	363	**0.57**	**0.42**	**0.77**	**0.0003**
	Mood disorders	48058	483	0.83	0.66	1.04	0.1106
**VI**	**Diseases of the nervous system**	46901	2255	0.92	0.83	1.03	0.164
	Sleep disorders	48472	1206	**0.79**	**0.68**	**0.93**	**0.0043**
**IX**	**Diseases of the circulatory system**	45534	3552	1.09	1.00	1.19	0.0558
	Hypertension	44864	2009	**0.79**	**0.70**	**0.90**	**0.0003**
	Ischemic heart diseases	47987	1047	0.99	0.83	1.18	0.8822
	Angina pectoris	48156	381	1.06	0.80	1.42	0.6752
	Heart failure	48397	259	0.84	0.57	1.23	0.3694
	Cerebrovascular diseases	48307	628	0.93	0.73	1.17	0.5104
	Stroke	48352	527	0.85	0.65	1.11	0.2309
	Intracerebral haemorrhage	48459	100	0.85	0.46	1.57	0.5977
**X**	**Diseases of the respiratory system**	45093	2639	1.00	0.91	1.10	0.9983
	Chronic obstructive bronchitis	48414	271	0.78	0.53	1.15	0.2044
**XI**	**Diseases of the digestive system**	44780	4130	1.00	0.93	1.09	0.9096
**XII**	**Diseases of the skin**	47964	448	**0.76**	**0.59**	**0.98**	**0.0348**
**XIII**	**Diseases of the musculoskeletal system**	42691	5348	0.99	0.93	1.06	0.822
	Osteoarthritis	48472	2431	0.90	0.81	1.02	0.0903
	Soft tissue disorders	48472	2026	0.97	0.87	1.09	0.651
**XIV**	**Diseases of the genitourinary system**	43979	3899	1.07	0.99	1.16	0.0966
**XV**	**Pregnancy complications: hypertension**	36748	201	**1.37**	**1.03**	**1.83**	**0.0284**
**XVIII**	Digestive and abdominal symptoms	48472	933	0.92	0.78	1.08	0.3196
**XIX**	**Poisoning**	48472	229	0.76	0.54	1.08	0.123
**XX**	**Self-harm**	38140	191	0.75	0.51	1.09	0.1333
		**Decrease in income**					
	**Death**	49284	1420	**1.21**	**1.07**	**1.37**	**0.0029**
**IV**	**Endocrine diseases**	48065	3016	**1.13**	**1.03**	**1.23**	**0.0069**
	Diabetes	48477	2453	**1.17**	**1.07**	**1.29**	**0.0011**
	Obesity	49284	162	1.16	0.81	1.67	0.4062
**V**	**Mental and behavioural disorders**	48662	723	**1.26**	**1.07**	**1.49**	**0.0069**
	Disorders due to substance abuse	49284	254	**1.65**	**1.26**	**2.16**	**0.0003**
	Psychotic disorders	48979	256	**1.33**	**1.01**	**1.75**	**0.0448**
	Mood disorders	48955	344	1.24	0.97	1.57	0.0876
**VI**	**Diseases of the nervous system**	47588	2359	1.02	0.93	1.13	0.6694
	Sleep disorders	49284	1260	1.12	0.98	1.28	0.1049
**IX**	**Diseases of the circulatory system**	45781	3744	**1.10**	**1.02**	**1.19**	**0.0163**
	Hypertension	45401	2358	1.01	0.91	1.12	0.8633
	Ischemic heart diseases	48730	1190	1.12	0.98	1.29	0.1064
	Angina pectoris	48912	512	1.11	0.90	1.37	0.3488
	Heart failure	49201	219	**1.42**	**1.05**	**1.92**	**0.0216**
	Cerebrovascular diseases	49080	577	**1.34**	**1.11**	**1.62**	**0.0024**
	Stroke	49124	490	**1.37**	**1.12**	**1.68**	**0.0022**
	Intracerebral haemorrhage	49260	71	**2.16**	**1.32**	**3.52**	**0.0021**
**X**	**Diseases of the respiratory system**	46166	2361	**1.21**	**1.10**	**1.33**	**<.0001**
	Chronic obstructive bronchitis	49251	162	**1.45**	**1.02**	**2.05**	**0.0371**
**XI**	**Diseases of the digestive system**	45579	4307	1.05	0.97	1.13	0.2116
**XII**	**Diseases of the skin**	48821	416	**1.27**	**1.02**	**1.59**	**0.0328**
**XIII**	**Diseases of the musculoskeletal system**	43026	6157	**1.08**	**1.02**	**1.15**	**0.0101**
	Osteoarthritis	49284	2746	1.02	0.93	1.12	0.6202
	Soft tissue disorders	49284	2336	1.00	0.90	1.10	0.9437
**XIV**	**Diseases of the genitourinary system**	44290	4237	1.00	0.93	1.08	0.9817
**XV**	**Pregnancy complications: hypertension**	37806	85	0.98	0.62	1.55	0.9367
**XVIII**	Digestive and abdominal symptoms	49284	868	**1.18**	**1.01**	**1.38**	**0.0346**
**XIX**	**Poisoning**	49284	154	**1.62**	**1.15**	**2.28**	**0.0054**
**XX**	**Self-harm**	41404	141	**1.50**	**1.05**	**2.16**	**0.028**

ICD-chapter		Total	Cases	HazardRatio	Lower CI	Upper CI	ProbChiSq
		**Exposure: Change in neighbourhood characteristic**					
		**Decrease in unemployment**					
	**Death**	50533	1613	**0.88**	**0.79**	**0.99**	**0.0304**
**IV**	**Endocrine diseases**	49774	3180	**0.878**	**0.811**	**0.952**	**0.0015**
	Diabetes	49508	2617	**0.86**	**0.79**	**0.94**	**0.0009**
	Obesity	50533	200	0.85	0.62	1.16	0.3086
**V**	**Mental and behavioural disorders**	49619	930	**0.81**	**0.70**	**0.94**	**0.0052**
	Disorders due to substance abuse	50533	313	**0.69**	**0.52**	**0.90**	**0.0071**
	Psychotic disorders	50025	353	**0.72**	**0.56**	**0.92**	**0.0091**
	Mood disorders	50105	476	0.83	0.68	1.02	0.0764
**VI**	**Diseases of the nervous system**	48718	2398	**0.84**	**0.77**	**0.92**	**0.0003**
	Sleep disorders	50533	1276	**0.75**	**0.66**	**0.85**	**<.0001**
**IX**	**Diseases of the circulatory system**	47079	3829	0.97	0.90	1.04	0.3877
	Hypertension	46240	2262	0.98	0.89	1.07	0.642
	Ischemic heart diseases	49934	1209	0.95	0.84	1.08	0.463
	Angina pectoris	50141	470	1.02	0.83	1.25	0.8541
	Heart failure	50434	255	0.74	0.55	1.00	0.0523
	Cerebrovascular diseases	50317	633	0.86	0.72	1.04	0.1152
	Stroke	50361	536	**0.80**	**0.66**	**0.98**	**0.0323**
	Intracerebral haemorrhage	50507	101	0.77	0.48	1.23	0.2715
**X**	**Diseases of the respiratory system**	47117	2658	0.92	0.85	1.00	0.0545
	Chronic obstructive bronchitis	50473	246	**0.70**	**0.51**	**0.96**	**0.0259**
**XI**	**Diseases of the digestive system**	46551	4333	**0.92**	**0.87**	**0.99**	**0.0192**
**XII**	**Diseases of the skin**	50016	486	**0.80**	**0.65**	**0.98**	**0.0328**
**XIII**	**Diseases of the musculoskeletal system**	43845	5842	0.97	0.92	1.03	0.3682
	Osteoarthritis	50533	2667	**0.91**	**0.84**	**0.99**	**0.0354**
	Soft tissue disorders	50533	2256	1.00	0.91	1.09	0.933
**XIV**	**Diseases of the genitourinary system**	45479	4096	0.98	0.92	1.05	0.5943
**XV**	**Pregnancy complications: hypertension**	38357	170	**1.58**	**1.17**	**2.13**	**0.0031**
**XVIII**	Digestive and abdominal symptoms	50533	982	**0.81**	**0.70**	**0.93**	**0.0029**
**XIX**	**Poisoning**	50533	230	**0.59**	**0.43**	**0.81**	**0.0013**
**XX**	**Self-harm**	39145	197	0.80	0.58	1.11	0.1806
		**Increase in unemployment**					
	**Death**	51116	1444	1.12	1.00	1.24	0.0521
**IV**	**Endocrine diseases**	50524	2979	**1.13**	**1.04**	**1.22**	**0.0022**
	Diabetes	50342	2406	**1.13**	**1.04**	**1.23**	**0.004**
	Obesity	51116	162	**1.54**	**1.12**	**2.10**	**0.0072**
**V**	**Mental and behavioural disorders**	50430	767	1.09	0.94	1.27	0.2427
	Disorders due to substance abuse	51116	274	**1.38**	**1.08**	**1.76**	**0.009**
	Psychotic disorders	50756	275	0.88	0.68	1.14	0.3262
	Mood disorders	50765	366	1.01	0.81	1.26	0.902
**VI**	**Diseases of the nervous system**	49509	2287	**1.12**	**1.03**	**1.23**	**0.0087**
	Sleep disorders	51116	1219	1.12	0.99	1.26	0.071
**IX**	**Diseases of the circulatory system**	47882	3572	**1.12**	**1.04**	**1.20**	**0.0022**
	Hypertension	47630	2169	**1.15**	**1.05**	**1.25**	**0.0026**
	Ischemic heart diseases	50635	1061	**1.29**	**1.14**	**1.46**	**<.0001**
	Angina pectoris	50789	437	1.16	0.95	1.41	0.1432
	Heart failure	51051	227	**1.32**	**1.01**	**1.73**	**0.0442**
	Cerebrovascular diseases	50946	582	1.09	0.92	1.30	0.3264
	Stroke	50994	490	1.08	0.89	1.30	0.445
	Intracerebral haemorrhage	51105	73	1.07	0.65	1.74	0.7996
**X**	**Diseases of the respiratory system**	47791	2423	1.08	1.00	1.18	0.0613
	Chronic obstructive bronchitis	51083	195	**1.39**	**1.04**	**1.85**	**0.0248**
**XI**	**Diseases of the digestive system**	47374	4241	**1.11**	**1.04**	**1.19**	**0.0011**
**XII**	**Diseases of the skin**	50614	389	**1.38**	**1.12**	**1.69**	**0.0021**
**XIII**	**Diseases of the musculoskeletal system**	45270	5854	**1.06**	**1.01**	**1.12**	**0.0284**
	Osteoarthritis	51116	2586	**1.14**	**1.05**	**1.24**	**0.0012**
	Soft tissue disorders	51116	2182	**1.14**	**1.04**	**1.25**	**0.0035**
**XIV**	**Diseases of the genitourinary system**	46254	4166	1.04	0.97	1.11	0.2436
**XV**	**Pregnancy complications: hypertension**	39040	124	0.75	0.51	1.09	0.1272
**XVIII**	Digestive and abdominal symptoms	51116	845	**1.16**	**1.00**	**1.33**	**0.0464**
**XIX**	**Poisoning**	51116	161	1.32	0.96	1.81	0.0912
**XX**	**Self-harm**	43149	141	1.36	0.97	1.91	0.0746

ICD-chapter		Total	Cases	HazardRatio	Lower CI	Upper CI	ProbChiSq
		**Exposure: Change in neighbourhood characteristic**					
		**Increase in green space**					
	**Death**	55386	1708	**0.79**	**0.69**	**0.90**	**0.0004**
**IV**	**Endocrine diseases**	53938	3356	**0.89**	**0.81**	**0.97**	**0.0075**
	Diabetes	54391	2721	**0.88**	**0.80**	**0.98**	**0.0146**
	Obesity	55386	193	0.72	0.50	1.03	0.0747
**V**	**Mental and behavioural disorders**	54432	1020	0.90	0.77	1.05	0.1718
	Disorders due to substance abuse	55386	337	0.92	0.71	1.19	0.5146
	Psychotic disorders	54847	395	**0.69**	**0.53**	**0.89**	**0.0045**
	Mood disorders	54930	513	**0.80**	**0.64**	**0.99**	**0.0378**
**VI**	**Diseases of the nervous system**	53641	2564	1.01	0.92	1.11	0.8021
	Sleep disorders	55386	1389	1.11	0.98	1.26	0.1074
**IX**	**Diseases of the circulatory system**	51971	3962	**1.12**	**1.04**	**1.21**	**0.0041**
	Hypertension	51482	2322	0.97	0.88	1.07	0.5484
	Ischemic heart diseases	54842	1160	0.97	0.83	1.12	0.6701
	Angina pectoris	55025	431	1.21	0.96	1.53	0.102
	Heart failure	55310	257	0.73	0.51	1.04	0.0806
	Cerebrovascular diseases	55214	681	0.98	0.81	1.19	0.8568
	Stroke	55254	564	0.89	0.71	1.11	0.2914
	Intracerebral haemorrhage	55369	102	**0.52**	**0.28**	**0.97**	**0.0394**
**X**	**Diseases of the respiratory system**	51627	2929	1.09	1.00	1.18	0.058
	Chronic obstructive bronchitis	55330	277	0.91	0.66	1.25	0.5609
**XI**	**Diseases of the digestive system**	51237	4697	1.06	0.99	1.13	0.117
**XII**	**Diseases of the skin**	54828	468	0.94	0.76	1.17	0.5972
**XIII**	**Diseases of the musculoskeletal syst**	48856	6172	1.06	1.00	1.13	0.0528
	Osteoarthritis	55386	2740	1.00	0.91	1.10	0.9938
	Soft tissue disorders	55386	2314	**1.12**	**1.02**	**1.23**	**0.0225**
**XIV**	**Diseases of the genitourinary system**	50147	4505	**1.13**	**1.05**	**1.21**	**0.0005**
**XV**	**Pregnancy complications: hypertensi**	42375	225	1.26	0.96	1.65	0.0998
**XVIII**	Digestive and abdominal symptoms	55386	1062	**1.33**	**1.16**	**1.52**	**<.0001**
**XIX**	**Poisoning**	55386	248	0.86	0.63	1.17	0.327
**XX**	**Self-harm**	44832	216	0.75	0.53	1.06	0.1033
		**Decrease in green space**					
	**Death**	55465	1651	0.99	0.88	1.12	0.8805
**IV**	**Endocrine diseases**	54047	3444	**1.09**	**1.01**	**1.18**	**0.0335**
	Diabetes	54494	2796	1.09	1.00	1.19	0.0613
	Obesity	55465	209	1.01	0.74	1.39	0.9525
**V**	**Mental and behavioural disorders**	54675	862	**1.18**	**1.01**	**1.38**	**0.0329**
	Disorders due to substance abuse	55465	333	1.28	0.99	1.63	0.0555
	Psychotic disorders	55060	301	1.16	0.89	1.50	0.2688
	Mood disorders	55073	410	1.15	0.92	1.44	0.2136
**VI**	**Diseases of the nervous system**	53456	2740	1.01	0.92	1.11	0.8507
	Sleep disorders	55465	1397	**0.83**	**0.72**	**0.95**	**0.0053**
**IX**	**Diseases of the circulatory system**	51545	4280	1.04	0.97	1.12	0.298
	Hypertension	50923	2617	1.06	0.96	1.16	0.2504
	Ischemic heart diseases	54824	1359	1.04	0.91	1.18	0.5986
	Angina pectoris	55040	593	0.88	0.72	1.09	0.2401
	Heart failure	55365	276	0.73	0.53	1.02	0.0614
	Cerebrovascular diseases	55208	671	0.99	0.82	1.19	0.8814
	Stroke	55267	564	0.99	0.81	1.22	0.9308
	Intracerebral haemorrhage	55436	85	1.20	0.73	1.98	0.4796
**X**	**Diseases of the respiratory system**	51824	2758	1.07	0.98	1.16	0.1542
	Chronic obstructive bronchitis	55418	206	1.01	0.72	1.42	0.9407
**XI**	**Diseases of the digestive system**	51164	4947	1.02	0.96	1.09	0.5462
**XII**	**Diseases of the skin**	54919	515	1.09	0.89	1.34	0.4039
**XIII**	**Diseases of the musculoskeletal syst**	48294	7000	**0.94**	**0.89**	**1.00**	**0.0353**
	Osteoarthritis	55465	3146	**0.91**	**0.83**	**0.99**	**0.0275**
	Soft tissue disorders	55465	2723	**0.88**	**0.80**	**0.97**	**0.0083**
**XIV**	**Diseases of the genitourinary system**	49847	4785	0.94	0.88	1.00	0.0652
**XV**	**Pregnancy complications: hypertensi**	41635	127	1.03	0.72	1.48	0.8783
**XVIII**	Digestive and abdominal symptoms	55465	1041	0.96	0.83	1.11	0.5726
**XIX**	**Poisoning**	55465	186	1.29	0.93	1.77	0.126
**XX**	**Self-harm**	43184	144	1.37	0.96	1.96	0.084

eTable 9. Prevalent and new diseases in participants who moved (N = 42 916) compared to those who did not move residential address (N = 71 705) during the observation period

	Odds ratio of prevalent disease for movers compared to non-movers						Odds ratio of new disease for movers compared to non-movers					
Health outcome	N(total)	N(cases)	OddsRatio	LowerCL	UpperCL	ProbChiSq	N(total)	N(cases)	HazardRatio	LowerCL	UpperCL	ProbChiSq
Death	114621	0	--	--	--	--	114621	3426	1.13	1.04	1.23	0.0030
Infections	114621	2141	1.22	1.11	1.34	<.0001	112480	3029	1.14	1.05	1.24	0.0018
Bacterial infections	114621	1653	1.18	1.06	1.32	0.0030	114621	2804	1.13	1.03	1.23	0.0067
Viral infections	114621	494	1.30	1.07	1.59	0.0099	114621	454	1.21	0.98	1.49	0.0778
Cancer	114621	2117	0.98	0.88	1.09	0.6626	112504	5498	0.91	0.86	0.98	0.0077
Colorectal cancer	114621	88	0.92	0.53	1.57	0.7493	114533	522	1.14	0.93	1.40	0.2110
Lung cancer	114621	24	0.79	0.26	2.35	0.6649	114597	291	1.22	0.93	1.61	0.1530
Melanoma	114621	186	0.87	0.61	1.24	0.4382	114435	1278	0.96	0.84	1.10	0.5785
Breast cancer	86898	1099	0.95	0.82	1.10	0.4924	85799	2283	0.83	0.75	0.92	0.0005
Prostate cancer	27723	92	0.91	0.52	1.60	0.7523	27631	529	0.94	0.75	1.16	0.5460
Kidney cancer	114621	34	0.99	0.42	2.31	0.9781	114587	142	1.07	0.72	1.60	0.7412
Brain cancer	114621	27	1.03	0.43	2.46	0.9476	114594	118	1.46	0.97	2.20	0.0673
Leukaemia, lymphoma	114621	241	1.08	0.80	1.45	0.6312	114380	606	0.96	0.79	1.17	0.6684
Diseases of the blood	114621	403	1.20	0.96	1.50	0.1113	114218	617	1.08	0.90	1.31	0.3999
Anemia	114621	184	1.16	0.83	1.62	0.3812	114621	369	1.02	0.80	1.31	0.8608
Endocrine diseases	114621	2918	1.08	0.99	1.18	0.0692	111703	6925	0.97	0.92	1.03	0.3198
Diabetes	114621	1998	1.10	0.99	1.22	0.0690	112623	5607	0.96	0.90	1.03	0.2557
Obesity	114621	63	1.21	0.69	2.13	0.5061	114621	415	1.11	0.89	1.38	0.3529
Mental and behavioural disorders	114621	1837	1.71	1.54	1.90	<.0001	112784	1938	1.20	1.08	1.33	0.0005
Dementia	114621	44	1.90	0.99	3.66	0.0554	114577	525	1.18	0.96	1.47	0.1231
Disorders due to substance abuse	114621	409	2.53	2.05	3.14	<.0001	114621	694	1.32	1.11	1.56	0.0013
Psychotic disorders	114621	981	1.23	1.07	1.42	0.0048	113640	717	1.41	1.19	1.66	<.0001
Mood disorders	114621	893	1.81	1.56	2.09	<.0001	113728	951	1.25	1.08	1.45	0.0023
Neurotic disorders	114621	389	1.64	1.31	2.05	<.0001	114232	321	1.46	1.14	1.87	0.0027
Diseases of the nervous system	114621	3835	1.07	0.99	1.16	0.0738	110786	5417	1.00	0.94	1.07	0.9756
Parkinson disease	114621	84	1.22	0.73	2.06	0.4476	114537	261	0.85	0.62	1.18	0.3280
Multiple sclerosis	114621	172	1.11	0.79	1.56	0.5619	114449	237	0.93	0.70	1.25	0.6349
Epilepsy	114621	1167	1.09	0.96	1.24	0.2044	113454	546	1.13	0.93	1.38	0.2208
Headaches	114621	350	1.43	1.13	1.81	0.0029	114621	374	0.99	0.79	1.26	0.9596
TIA	114621	182	1.12	0.78	1.60	0.5394	114439	791	1.03	0.87	1.23	0.6969
Sleep disorders	114621	821	1.09	0.92	1.28	0.3281	114621	2845	1.03	0.95	1.13	0.4561
Diseases of the eye	114621	1635	1.11	0.99	1.25	0.0677	112986	5466	1.09	1.02	1.16	0.0148
Diseases of the ear	114621	956	1.01	0.87	1.18	0.8647	113665	930	0.99	0.86	1.16	0.9428
Diseases of the circulatory system	114621	7464	1.03	0.97	1.09	0.3821	107157	8403	1.05	0.99	1.10	0.0867
Hypertension	114621	8549	0.94	0.89	0.99	0.0257	106072	5003	0.90	0.84	0.96	0.0012
Ischemic heart diseases	114621	1199	1.05	0.90	1.21	0.5456	113422	2554	1.04	0.94	1.14	0.4843
Angina pectoris	114621	796	0.99	0.82	1.19	0.9165	113825	1034	1.00	0.86	1.16	0.9730
Myocardial infarction	114621	296	1.07	0.80	1.42	0.6651	114325	873	0.96	0.81	1.13	0.6018
Pulmonary embolism	114621	127	1.15	0.77	1.72	0.4924	114621	442	1.23	0.99	1.53	0.0615
Arrhythmias	114621	1014	1.18	1.02	1.37	0.0249	113607	4060	1.10	1.02	1.18	0.0124
Heart failure	114621	181	1.32	0.94	1.85	0.1052	114440	539	0.95	0.77	1.18	0.6574
Cerebrovascular diseases	114621	440	1.16	0.93	1.44	0.1992	114181	1380	1.20	1.06	1.36	0.0041
Stroke	114621	337	1.11	0.86	1.43	0.4353	114284	1153	1.20	1.04	1.37	0.0106
Intracerebral haemorrhage	114621	48	1.54	0.82	2.91	0.1796	114573	191	1.35	0.98	1.88	0.0699
Cerebral infarction	114621	191	1.26	0.90	1.76	0.1738	114430	811	1.18	1.00	1.40	0.0444
Arteriosclerosis	114621	94	1.29	0.79	2.09	0.3122	114527	223	1.28	0.93	1.75	0.1280
Deep vein thrombosis	114621	349	1.17	0.92	1.50	0.2033	114621	451	1.36	1.10	1.67	0.0042
Diseases of the respiratory system	114621	7781	1.11	1.05	1.17	0.0001	106840	5873	1.15	1.09	1.22	<.0001
Influenza and Pneumonia	114621	1172	1.00	0.88	1.14	0.9846	114621	2376	1.16	1.06	1.28	0.0014
Chronic obstructive bronchitis	114621	104	1.33	0.85	2.08	0.2167	114517	491	1.31	1.06	1.61	0.0108
Asthma	114621	5486	1.10	1.03	1.17	0.0028	109135	3212	1.08	1.00	1.17	0.0491
Diseases of the digestive system	114621	8695	1.03	0.98	1.09	0.2337	105926	9891	1.04	0.99	1.09	0.0985
Appendicitis	114621	1453	1.07	0.95	1.21	0.2380	114621	1146	1.04	0.91	1.19	0.5364
Inflammatory bowel disease	114621	1063	1.09	0.95	1.26	0.2021	113558	825	1.10	0.94	1.28	0.2396
Diseases of liver	114621	249	1.09	0.82	1.46	0.5592	114621	849	1.25	1.07	1.46	0.0052
Alcoholic liver disease	114621	44	1.53	0.79	2.98	0.2117	114621	205	1.09	0.79	1.51	0.6060
Pancreatitis	114621	178	1.10	0.78	1.55	0.5874	114621	309	1.13	0.87	1.46	0.3605
Diseases of the skin	114621	1156	1.26	1.10	1.43	0.0006	113465	1008	1.07	0.93	1.24	0.3274
Infections and excema	114621	606	1.23	1.02	1.47	0.0275	114621	519	1.09	0.90	1.33	0.3767
Diseases of the musculoskeletal system	114621	14020	1.09	1.04	1.13	<.0001	100601	13422	1.01	0.97	1.05	0.5626
Rheumatoid arthritis and related disorders	114621	2060	1.04	0.94	1.15	0.4619	112561	2052	0.98	0.88	1.08	0.6623
Gout	114621	142	0.73	0.47	1.15	0.1752	114479	235	1.00	0.73	1.36	0.9817
Osteoarthritis	114621	2340	0.94	0.85	1.05	0.2777	114621	5956	0.96	0.90	1.02	0.2086
Sciatica	114621	1477	1.04	0.92	1.17	0.5202	114621	1489	1.00	0.89	1.12	0.9653
Back pain	114621	627	1.04	0.86	1.25	0.7109	114621	631	1.11	0.93	1.33	0.2452
Soft tissue disorders	114621	3753	1.08	1.00	1.16	0.0550	114621	5124	0.95	0.89	1.01	0.1242
Diseases of the genitourinary system	114621	11122	1.08	1.03	1.13	0.0012	103499	9502	1.06	1.01	1.11	0.0218
Renal failure	114621	81	1.27	0.77	2.08	0.3478	114540	335	1.22	0.95	1.57	0.1169
Pregnancy complications	86898	5342	0.84	0.79	0.90	<.0001	81556	1797	1.40	1.24	1.59	<.0001
Spontaneous abortion	86898	967	0.78	0.67	0.90	0.0006	86898	372	1.40	1.07	1.82	0.0139
Hypertension in pregnancy	86898	740	0.86	0.72	1.01	0.0654	86898	375	1.54	1.16	2.04	0.0026
Diabetes in pregnancy	86898	1087	0.81	0.70	0.93	0.0024	86898	416	1.22	0.95	1.56	0.1133
Circulatory and respiratory symptoms	114621	1673	1.25	1.12	1.40	<.0001	114621	1738	1.27	1.14	1.42	<.0001
Digestive and abdominal symptoms	114621	2515	1.21	1.11	1.32	<.0001	114621	2153	1.15	1.04	1.27	0.0047
Injury	114621	7423	1.15	1.09	1.22	<.0001	114621	9855	1.07	1.02	1.12	0.0030
Poisoning	114621	386	2.56	2.05	3.21	<.0001	114621	453	1.49	1.22	1.84	0.0001
Road accidents	91130	583	1.25	1.04	1.50	0.0163	91130	672	1.17	0.99	1.39	0.0736
Falls	91130	3242	1.12	1.03	1.21	0.0082	91130	4044	1.16	1.08	1.24	0.0001
Self-harm	91130	236	2.79	2.09	3.71	<.0001	91130	369	1.41	1.12	1.76	0.0035

eTable 10. Association of favourable (upper panel) and unfavourable change in neighbourhood characteristics with 30 subsequent health outcomes in non-movers.

		Exposure: Change in neighbourhood characteristic					
		Increase in education					
ICD-chapter		Total	Cases	HazardRatio	Lower CI	Upper CI	ProbChiSq
	**Death**	31952	1318	0.98	0.83	1.15	0.7859
**IV**	**Endocrine diseases**	30892	2553	**0.89**	**0.79**	**0.99**	**0.0387**
	Diabetes	31213	2147	0.89	0.79	1.01	0.0823
	Obesity	31952	137	**0.54**	**0.31**	**0.96**	**0.0359**
**V**	**Mental and behavioural disorders**	31475	604	0.87	0.68	1.10	0.2396
	Disorders due to substance abuse	31952	246	0.69	0.46	1.05	0.0813
	Psychotic disorders	31630	193	0.76	0.49	1.19	0.2318
	Mood disorders	31719	261	1.14	0.82	1.58	0.4428
**VI**	**Diseases of the nervous system**	30638	1778	0.94	0.82	1.08	0.3588
	Sleep disorders	31952	963	0.93	0.78	1.11	0.4186
**IX**	**Diseases of the circulatory system**	29410	2813	0.97	0.87	1.08	0.5327
	Hypertension	28547	1646	0.98	0.85	1.12	0.7618
	Ischemic heart diseases	31468	916	1.02	0.84	1.22	0.8654
	Angina pectoris	31626	368	1.17	0.89	1.55	0.2661
	Heart failure	31884	213	0.85	0.56	1.30	0.4595
	Cerebrovascular diseases	31789	487	1.18	0.92	1.51	0.1961
	Stroke	31822	423	1.08	0.82	1.42	0.574
	Intracerebral haemorrhage	31935	73	0.75	0.36	1.58	0.4526
**X**	**Diseases of the respiratory system**	30033	1692	0.93	0.81	1.07	0.3213
	Chronic obstructive bronchitis	31904	191	0.62	0.38	1.03	0.0625
**XI**	**Diseases of the digestive system**	29306	2962	0.91	0.82	1.01	0.084
**XII**	**Diseases of the skin**	31645	309	1.06	0.78	1.45	0.7067
**XIII**	**Diseases of the musculoskeletal system**	27290	4229	0.93	0.86	1.02	0.1142
	Osteoarthritis	31952	2248	**0.87**	**0.77**	**0.99**	**0.0285**
	Soft tissue disorders	31952	1673	1.00	0.87	1.14	0.957
**XIV**	**Diseases of the genitourinary system**	28550	2763	1.00	0.90	1.11	0.9831
**XV**	**Pregnancy complications: hypertension in pre**	24426	29	1.63	0.69	3.84	0.2637
**XVIII**	Digestive and abdominal symptoms	31952	642	**0.63**	**0.48**	**0.81**	**0.0004**
**XIX**	**Poisoning**	31952	128	**0.53**	**0.28**	**0.98**	**0.0432**
**XX**	**Self-harm**	25466	107	0.80	0.46	1.41	0.4469
		Decrease in education					
	**Death**	33592	1098	1.03	0.89	1.19	0.7286
**IV**	**Endocrine diseases**	32755	2156	1.07	0.97	1.19	0.1913
	Diabetes	33028	1745	1.09	0.97	1.22	0.1652
	Obesity	33592	91	1.16	0.70	1.90	0.5721
**V**	**Mental and behavioural disorders**	33194	507	1.06	0.86	1.32	0.5764
	Disorders due to substance abuse	33592	151	1.08	0.74	1.59	0.6944
	Psychotic disorders	33351	167	1.37	0.96	1.94	0.0808
	Mood disorders	33400	244	1.01	0.73	1.38	0.9685
**VI**	**Diseases of the nervous system**	32439	1635	1.07	0.94	1.20	0.3036
	Sleep disorders	33592	870	1.13	0.96	1.33	0.147
**IX**	**Diseases of the circulatory system**	31156	2712	1.01	0.92	1.11	0.88
	Hypertension	30783	1707	0.99	0.87	1.12	0.8304
	Ischemic heart diseases	33197	894	1.01	0.85	1.19	0.9366
	Angina pectoris	33322	367	1.13	0.88	1.45	0.3424
	Heart failure	33544	180	1.04	0.72	1.49	0.8512
	Cerebrovascular diseases	33468	442	0.86	0.67	1.10	0.221
	Stroke	33502	367	0.83	0.63	1.09	0.176
	Intracerebral haemorrhage	33584	58	1.27	0.68	2.37	0.447
**X**	**Diseases of the respiratory system**	31660	1611	**1.14**	**1.01**	**1.29**	**0.033**
	Chronic obstructive bronchitis	33571	144	0.94	0.62	1.43	0.7727
**XI**	**Diseases of the digestive system**	31013	2960	1.09	1.00	1.20	0.0536
**XII**	**Diseases of the skin**	33293	283	0.96	0.71	1.30	0.7955
**XIII**	**Diseases of the musculoskeletal system**	29343	4130	1.07	0.99	1.16	0.0722
	Osteoarthritis	33592	1909	**1.17**	**1.05**	**1.30**	**0.0053**
	Soft tissue disorders	33592	1502	**1.14**	**1.01**	**1.29**	**0.0378**
**XIV**	**Diseases of the genitourinary system**	30178	2869	1.05	0.96	1.16	0.2769
**XV**	**Pregnancy complications: hypertension in pre**	25885	32	0.61	0.23	1.60	0.3132
**XVIII**	Digestive and abdominal symptoms	33592	537	0.98	0.79	1.21	0.8334
**XIX**	**Poisoning**	33592	98	0.74	0.43	1.27	0.2786
**XX**	**Self-harm**	29152	93	0.81	0.47	1.39	0.4425

ICD-chapter		Total	Cases	HazardRatio	Lower CI	Upper CI	ProbChiSq
		**Exposure: Change in neighbourhood characteristic**					
		**Increase in income**					
	**Death**	26916	1181	**0.64**	**0.49**	**0.83**	**0.0008**
**IV**	**Endocrine diseases**	26060	2140	**0.82**	**0.70**	**0.97**	**0.0189**
	Diabetes	26309	1788	**0.80**	**0.66**	**0.96**	**0.0152**
	Obesity	26916	105	0.69	0.34	1.43	0.3203
**V**	**Mental and behavioural disorders**	26456	546	0.93	0.69	1.26	0.651
	Disorders due to substance abuse	26916	198	0.54	0.29	1.02	0.0575
	Psychotic disorders	26603	185	0.71	0.40	1.26	0.2418
	Mood disorders	26715	247	1.30	0.89	1.89	0.1828
**VI**	**Diseases of the nervous system**	25890	1449	1.04	0.87	1.24	0.7043
	Sleep disorders	26916	794	1.01	0.80	1.29	0.9143
**IX**	**Diseases of the circulatory system**	24882	2350	1.05	0.91	1.22	0.4799
	Hypertension	24175	1367	0.87	0.71	1.06	0.1551
	Ischemic heart diseases	26543	765	1.00	0.77	1.30	0.9957
	Angina pectoris	26671	284	1.26	0.84	1.87	0.2595
	Heart failure	26872	189	1.10	0.65	1.86	0.7334
	Cerebrovascular diseases	26803	440	**0.56**	**0.36**	**0.88**	**0.0111**
	Stroke	26832	379	**0.49**	**0.29**	**0.83**	**0.0074**
	Intracerebral haemorrhage	26906	77	1.00	0.43	2.31	0.9982
**X**	**Diseases of the respiratory system**	25307	1490	1.01	0.85	1.21	0.8817
	Chronic obstructive bronchitis	26874	199	0.66	0.35	1.25	0.2015
**XI**	**Diseases of the digestive system**	24714	2447	1.01	0.88	1.16	0.889
**XII**	**Diseases of the skin**	26658	251	**0.53**	**0.30**	**0.93**	**0.0266**
**XIII**	**Diseases of the musculoskeletal system**	23226	3305	1.06	0.94	1.19	0.3462
	Osteoarthritis	26916	1748	0.95	0.80	1.14	0.5887
	Soft tissue disorders	26916	1284	1.19	0.99	1.42	0.0582
**XIV**	**Diseases of the genitourinary system**	24201	2240	1.06	0.92	1.21	0.448
**XV**	**Pregnancy complications: hypertension in pre**	20628	32	**2.85**	**1.35**	**6.04**	**0.0062**
**XVIII**	Digestive and abdominal symptoms	26916	490	0.85	0.62	1.18	0.3404
**XIX**	**Poisoning**	26916	112	0.42	0.17	1.04	0.06
**XX**	**Self-harm**	21997	100	0.47	0.19	1.17	0.1051
		**Decrease in income**					
	**Death**	36110	1167	1.12	0.96	1.31	0.1634
**IV**	**Endocrine diseases**	35147	2425	1.10	0.98	1.23	0.11
	Diabetes	35470	1993	1.11	0.98	1.26	0.093
	Obesity	36110	114	1.14	0.69	1.89	0.6107
**V**	**Mental and behavioural disorders**	35738	535	1.20	0.95	1.51	0.1275
	Disorders due to substance abuse	36110	183	1.29	0.88	1.89	0.1971
	Psychotic disorders	35886	165	0.97	0.62	1.50	0.8761
	Mood disorders	35908	240	1.15	0.81	1.63	0.4297
**VI**	**Diseases of the nervous system**	34789	1815	1.02	0.89	1.17	0.7635
	Sleep disorders	36110	974	1.10	0.92	1.32	0.2833
**IX**	**Diseases of the circulatory system**	33344	2963	1.07	0.97	1.19	0.1701
	Hypertension	32868	1871	0.95	0.83	1.09	0.4886
	Ischemic heart diseases	35636	978	1.01	0.84	1.21	0.9043
	Angina pectoris	35783	418	1.01	0.77	1.34	0.9201
	Heart failure	36043	192	**1.72**	**1.22**	**2.42**	**0.0021**
	Cerebrovascular diseases	35947	455	**1.31**	**1.02**	**1.67**	**0.0319**
	Stroke	35982	384	**1.37**	**1.05**	**1.78**	**0.0186**
	Intracerebral haemorrhage	36095	49	1.85	0.94	3.62	0.0746
**X**	**Diseases of the respiratory system**	34024	1684	1.11	0.97	1.27	0.1327
	Chronic obstructive bronchitis	36085	128	1.40	0.90	2.18	0.1406
**XI**	**Diseases of the digestive system**	33311	3244	1.03	0.93	1.14	0.5854
**XII**	**Diseases of the skin**	35787	315	1.09	0.80	1.49	0.5938
**XIII**	**Diseases of the musculoskeletal system**	31228	4709	1.03	0.94	1.11	0.5482
	Osteoarthritis	36110	2250	1.00	0.88	1.12	0.9414
	Soft tissue disorders	36110	1762	0.92	0.80	1.05	0.224
**XIV**	**Diseases of the genitourinary system**	32310	3167	0.96	0.86	1.06	0.4034
**XV**	**Pregnancy complications: hypertension in pre**	27844	24	1.46	0.59	3.62	0.415
**XVIII**	Digestive and abdominal symptoms	36110	630	1.07	0.86	1.34	0.5286
**XIX**	**Poisoning**	36110	103	1.29	0.78	2.14	0.313
**XX**	**Self-harm**	30818	93	0.87	0.47	1.59	0.6413

ICD-chapter		Total	Cases	HazardRatio	Lower CI	Upper CI	ProbChiSq
		**Exposure: Change in neighbourhood characteristic**					
		**Decrease in unemployment**					
	**Death**	29302	1204	0.94	0.82	1.08	0.3813
**IV**	**Endocrine diseases**	28370	2281	**0.87**	**0.78**	**0.95**	**0.0039**
	Diabetes	28639	1911	**0.84**	**0.75**	**0.93**	**0.0012**
	Obesity	29302	112	0.98	0.64	1.50	0.9255
**V**	**Mental and behavioural disorders**	28846	551	0.82	0.67	1.01	0.0617
	Disorders due to substance abuse	29302	189	0.75	0.52	1.08	0.1164
	Psychotic disorders	28999	183	0.79	0.55	1.12	0.1858
	Mood disorders	29095	252	0.96	0.72	1.28	0.764
**VI**	**Diseases of the nervous system**	28104	1613	**0.84**	**0.75**	**0.95**	**0.0045**
	Sleep disorders	29302	850	**0.78**	**0.66**	**0.91**	**0.0025**
**IX**	**Diseases of the circulatory system**	27058	2670	1.00	0.91	1.09	0.9305
	Hypertension	26244	1574	**1.12**	**1.00**	**1.26**	**0.0415**
	Ischemic heart diseases	28872	922	0.98	0.84	1.14	0.7764
	Angina pectoris	29016	363	1.15	0.91	1.45	0.244
	Heart failure	29247	198	0.77	0.54	1.09	0.1385
	Cerebrovascular diseases	29168	460	0.93	0.75	1.15	0.5025
	Stroke	29200	402	0.87	0.68	1.10	0.2381
	Intracerebral haemorrhage	29290	81	0.86	0.51	1.46	0.58
**X**	**Diseases of the respiratory system**	27577	1533	**0.87**	**0.78**	**0.99**	**0.0274**
	Chronic obstructive bronchitis	29258	178	**0.62**	**0.41**	**0.93**	**0.0196**
**XI**	**Diseases of the digestive system**	26882	2726	0.94	0.86	1.03	0.1769
**XII**	**Diseases of the skin**	29015	282	**0.72**	**0.53**	**0.97**	**0.0286**
**XIII**	**Diseases of the musculoskeletal system**	25154	3803	0.99	0.92	1.06	0.7377
	Osteoarthritis	29302	1965	0.93	0.83	1.03	0.1482
	Soft tissue disorders	29302	1481	1.11	0.99	1.25	0.0707
**XIV**	**Diseases of the genitourinary system**	26292	2484	0.97	0.89	1.07	0.5726
**XV**	**Pregnancy complications: hypertension in pre**	22307	28	1.76	0.82	3.77	0.1448
**XVIII**	Digestive and abdominal symptoms	29302	547	0.85	0.69	1.04	0.1137
**XIX**	**Poisoning**	29302	115	**0.52**	**0.31**	**0.87**	**0.0134**
**XX**	**Self-harm**	24082	103	0.93	0.59	1.48	0.7708
		**Increase in unemployment**					
	**Death**	34638	1161	1.11	0.98	1.26	0.0953
**IV**	**Endocrine diseases**	33729	2337	**1.11**	**1.02**	**1.21**	**0.0195**
	Diabetes	34039	1913	**1.13**	**1.03**	**1.24**	**0.0143**
	Obesity	34638	110	**1.61**	**1.09**	**2.36**	**0.0155**
**V**	**Mental and behavioural disorders**	34248	537	1.00	0.83	1.20	0.9781
	Disorders due to substance abuse	34638	196	1.09	0.81	1.48	0.5751
	Psychotic disorders	34394	172	0.78	0.55	1.11	0.169
	Mood disorders	34436	243	1.03	0.78	1.36	0.815
**VI**	**Diseases of the nervous system**	33438	1699	1.08	0.98	1.20	0.1299
	Sleep disorders	34638	939	1.10	0.96	1.27	0.1777
**IX**	**Diseases of the circulatory system**	32015	2715	1.07	0.99	1.17	0.0935
	Hypertension	31644	1706	**1.14**	**1.03**	**1.26**	**0.0148**
	Ischemic heart diseases	34206	847	**1.19**	**1.03**	**1.37**	**0.0189**
	Angina pectoris	34341	350	1.16	0.92	1.45	0.2026
	Heart failure	34582	186	1.26	0.93	1.71	0.1372
	Cerebrovascular diseases	34492	442	1.02	0.83	1.25	0.8739
	Stroke	34526	368	0.99	0.79	1.24	0.9347
	Intracerebral haemorrhage	34625	48	0.59	0.29	1.22	0.1534
**X**	**Diseases of the respiratory system**	32618	1693	1.06	0.95	1.17	0.3118
	Chronic obstructive bronchitis	34614	153	1.38	0.99	1.92	0.0557
**XI**	**Diseases of the digestive system**	31971	3051	**1.14**	**1.05**	**1.23**	**0.0012**
**XII**	**Diseases of the skin**	34336	294	**1.28**	**1.01**	**1.64**	**0.0432**
**XIII**	**Diseases of the musculoskeletal system**	30095	4341	1.04	0.98	1.11	0.2275
	Osteoarthritis	34638	2092	**1.15**	**1.05**	**1.27**	**0.0025**
	Soft tissue disorders	34638	1613	1.10	0.99	1.22	0.0796
**XIV**	**Diseases of the genitourinary system**	31041	2999	1.04	0.96	1.12	0.3784
**XV**	**Pregnancy complications: hypertension in pre**	26820	28	0.78	0.34	1.78	0.556
**XVIII**	Digestive and abdominal symptoms	34638	586	1.10	0.92	1.31	0.2887
**XIX**	**Poisoning**	34638	103	1.09	0.72	1.66	0.6849
**XX**	**Self-harm**	29453	93	1.25	0.81	1.92	0.309

ICD-chapter		Total	Cases	HazardRatio	Lower CI	Upper CI	ProbChiSq
		**Exposure: Change in neighbourhood characteristic**					
		**Increase in green space**					
	**Death**	32361	1317	**0.71**	**0.59**	**0.85**	**0.0003**
**IV**	**Endocrine diseases**	31413	2485	**0.80**	**0.70**	**0.91**	**0.0005**
	Diabetes	31692	2068	**0.81**	**0.70**	**0.93**	**0.0034**
	Obesity	32361	98	0.89	0.47	1.66	0.7106
**V**	**Mental and behavioural disorders**	31897	612	**0.75**	**0.58**	**0.95**	**0.019**
	Disorders due to substance abuse	32361	209	0.74	0.50	1.10	0.133
	Psychotic disorders	32033	209	**0.56**	**0.35**	**0.90**	**0.0157**
	Mood disorders	32141	278	**0.64**	**0.44**	**0.92**	**0.0174**
**VI**	**Diseases of the nervous system**	31199	1750	1.07	0.93	1.23	0.3365
	Sleep disorders	32361	935	1.10	0.91	1.34	0.328
**IX**	**Diseases of the circulatory system**	29951	2851	1.03	0.92	1.15	0.6607
	Hypertension	29265	1712	1.02	0.88	1.17	0.8399
	Ischemic heart diseases	31930	902	1.03	0.84	1.26	0.8
	Angina pectoris	32072	338	**1.58**	**1.18**	**2.13**	**0.0024**
	Heart failure	32313	208	0.95	0.61	1.49	0.8286
	Cerebrovascular diseases	32239	502	0.79	0.59	1.04	0.0921
	Stroke	32266	425	0.80	0.59	1.09	0.1555
	Intracerebral haemorrhage	32348	80	0.99	0.50	1.92	0.9651
**X**	**Diseases of the respiratory system**	30546	1744	1.08	0.95	1.24	0.2422
	Chronic obstructive bronchitis	32325	205	0.80	0.49	1.30	0.3656
**XI**	**Diseases of the digestive system**	29826	3002	1.11	1.00	1.23	0.0546
**XII**	**Diseases of the skin**	32064	305	**0.65**	**0.45**	**0.93**	**0.019**
**XIII**	**Diseases of the musculoskeletal system**	28090	4154	1.06	0.97	1.16	0.1842
	Osteoarthritis	32361	2091	1.08	0.95	1.23	0.2586
	Soft tissue disorders	32361	1552	**1.16**	**1.01**	**1.34**	**0.0361**
**XIV**	**Diseases of the genitourinary system**	29141	2817	**1.21**	**1.09**	**1.34**	**0.0004**
**XV**	**Pregnancy complications: hypertension in pre**	24924	44	0.88	0.41	1.86	0.7288
**XVIII**	Digestive and abdominal symptoms	32361	589	1.16	0.92	1.45	0.2109
**XIX**	**Poisoning**	32361	128	0.66	0.37	1.17	0.1519
**XX**	**Self-harm**	26660	116	**0.38**	**0.15**	**0.92**	**0.0326**
		**Decrease in green space**					
	**Death**	39137	1269	1.02	0.88	1.18	0.7973
**IV**	**Endocrine diseases**	38028	2596	1.09	0.98	1.21	0.099
	Diabetes	38382	2117	1.09	0.98	1.22	0.1223
	Obesity	39137	149	1.14	0.76	1.72	0.5302
**V**	**Mental and behavioural disorders**	38649	577	1.01	0.81	1.27	0.9249
	Disorders due to substance abuse	39137	219	0.95	0.65	1.41	0.808
	Psychotic disorders	38855	188	0.73	0.47	1.13	0.1597
	Mood disorders	38893	258	1.04	0.75	1.44	0.807
**VI**	**Diseases of the nervous system**	37605	1985	0.99	0.87	1.11	0.8213
	Sleep disorders	39137	1057	**0.80**	**0.68**	**0.95**	**0.0092**
**IX**	**Diseases of the circulatory system**	36075	3139	0.97	0.88	1.06	0.4755
	Hypertension	35523	1941	1.06	0.94	1.19	0.3413
	Ischemic heart diseases	38603	1046	1.16	1.00	1.36	0.0578
	Angina pectoris	38776	461	1.02	0.80	1.31	0.8624
	Heart failure	39058	219	0.83	0.57	1.21	0.3259
	Cerebrovascular diseases	38940	504	1.07	0.84	1.35	0.5997
	Stroke	38984	424	1.05	0.81	1.36	0.7315
	Intracerebral haemorrhage	39118	57	0.76	0.36	1.61	0.472
**X**	**Diseases of the respiratory system**	36702	1877	0.99	0.87	1.12	0.8477
	Chronic obstructive bronchitis	39097	151	0.71	0.44	1.15	0.1637
**XI**	**Diseases of the digestive system**	35952	3473	1.05	0.96	1.14	0.3243
**XII**	**Diseases of the skin**	38784	338	0.95	0.71	1.29	0.7504
**XIII**	**Diseases of the musculoskeletal system**	33692	4991	**0.92**	**0.85**	**0.99**	**0.0255**
	Osteoarthritis	39137	2435	**0.88**	**0.79**	**0.98**	**0.0208**
	Soft tissue disorders	39137	1953	0.91	0.80	1.03	0.1213
**XIV**	**Diseases of the genitourinary system**	34904	3358	1.01	0.93	1.11	0.7929
**XV**	**Pregnancy complications: hypertension in pre**	29646	27	1.42	0.58	3.46	0.4387
**XVIII**	Digestive and abdominal symptoms	39137	719	1.02	0.84	1.25	0.8227
**XIX**	**Poisoning**	39137	113	1.27	0.79	2.05	0.324
**XX**	**Self-harm**	31606	90	1.09	0.66	1.81	0.7425

eTable 11. Association of favourable (upper panel) and unfavourable change in neighbourhood characteristics with 30 subsequent health outcomes in movers.

		Exposure: Change in neighbourhood characteristic					
		Increase in education					
ICD-chapter		Total	Cases	HazardRatio	Lower CI	Upper CI	ProbChiSq
	**Death**	18581	398	**0.67**	**0.53**	**0.83**	**0.0004**
**IV**	**Endocrine diseases**	18174	902	**0.80**	**0.70**	**0.93**	**0.0024**
	Diabetes	18295	703	**0.76**	**0.65**	**0.90**	**0.0011**
	Obesity	18581	93	0.72	0.46	1.11	0.1389
**V**	**Mental and behavioural disorders**	18144	356	**0.79**	**0.63**	**0.98**	**0.035**
	Disorders due to substance abuse	18581	136	**0.60**	**0.41**	**0.89**	**0.0113**
	Psychotic disorders	18395	151	0.87	0.62	1.22	0.4078
	Mood disorders	18386	206	0.75	0.55	1.00	0.0524
**VI**	**Diseases of the nervous system**	18080	770	0.98	0.84	1.13	0.738
	Sleep disorders	18581	382	0.88	0.71	1.09	0.2353
**IX**	**Diseases of the circulatory system**	17669	1109	0.91	0.80	1.03	0.1251
	Hypertension	17693	641	0.86	0.73	1.01	0.0728
	Ischemic heart diseases	18466	293	0.97	0.76	1.24	0.7991
	Angina pectoris	18515	114	0.81	0.54	1.21	0.3029
	Heart failure	18550	64	1.04	0.62	1.75	0.8874
	Cerebrovascular diseases	18528	164	0.98	0.71	1.36	0.9163
	Stroke	18539	138	1.10	0.78	1.56	0.5909
	Intracerebral haemorrhage	18572	24	0.86	0.36	2.04	0.7301
**X**	**Diseases of the respiratory system**	17084	1036	0.94	0.83	1.06	0.3176
	Chronic obstructive bronchitis	18569	68	1.02	0.62	1.69	0.9272
**XI**	**Diseases of the digestive system**	17245	1538	**0.82**	**0.74**	**0.91**	**0.0003**
**XII**	**Diseases of the skin**	18371	170	0.78	0.57	1.08	0.1358
**XIII**	**Diseases of the musculoskeletal system**	16555	1987	0.95	0.87	1.04	0.2599
	Osteoarthritis	18581	705	**0.84**	**0.71**	**0.98**	**0.0264**
	Soft tissue disorders	18581	800	0.90	0.78	1.04	0.1526
**XIV**	**Diseases of the genitourinary system**	16929	1534	0.98	0.88	1.09	0.6881
**XV**	**Pregnancy complications: hypertension in pre**	13931	127	0.75	0.52	1.08	0.1253
**XVIII**	Digestive and abdominal symptoms	18581	406	0.99	0.81	1.22	0.9511
**XIX**	**Poisoning**	18581	110	**0.61**	**0.39**	**0.93**	**0.022**
**XX**	**Self-harm**	13679	85	**0.54**	**0.34**	**0.88**	**0.0139**
		**Decrease in education**					
	**Death**	17524	310	1.11	0.88	1.39	0.3953
**IV**	**Endocrine diseases**	17191	666	**1.39**	**1.19**	**1.63**	**<.0001**
	Diabetes	17314	516	**1.34**	**1.13**	**1.60**	**0.0011**
	Obesity	17524	51	1.66	0.95	2.90	0.0732
**V**	**Mental and behavioural disorders**	17236	270	1.04	0.82	1.33	0.7407
	Disorders due to substance abuse	17524	79	**1.59**	**1.02**	**2.50**	**0.0422**
	Psychotic disorders	17405	125	0.92	0.64	1.33	0.6561
	Mood disorders	17365	149	0.84	0.60	1.18	0.3102
**VI**	**Diseases of the nervous system**	17070	638	1.06	0.90	1.25	0.4656
	Sleep disorders	17524	340	**1.29**	**1.04**	**1.60**	**0.0227**
**IX**	**Diseases of the circulatory system**	16726	942	1.06	0.93	1.21	0.41
	Hypertension	16847	535	1.12	0.94	1.34	0.1897
	Ischemic heart diseases	17438	220	1.31	1.00	1.72	0.0503
	Angina pectoris	17467	84	1.06	0.68	1.64	0.8068
	Heart failure	17507	36	1.76	0.90	3.44	0.0994
	Cerebrovascular diseases	17478	152	1.26	0.91	1.75	0.1623
	Stroke	17492	121	1.22	0.85	1.76	0.2889
	Intracerebral haemorrhage	17521	22	0.97	0.41	2.31	0.945
**X**	**Diseases of the respiratory system**	16131	854	**1.18**	**1.02**	**1.35**	**0.0216**
	Chronic obstructive bronchitis	17512	44	1.82	0.99	3.35	0.0536
**XI**	**Diseases of the digestive system**	16361	1329	1.10	0.98	1.23	0.0922
**XII**	**Diseases of the skin**	17321	136	**1.62**	**1.15**	**2.28**	**0.0054**
**XIII**	**Diseases of the musculoskeletal system**	15927	1652	**1.26**	**1.14**	**1.39**	**<.0001**
	Osteoarthritis	17524	518	**1.26**	**1.06**	**1.50**	**0.0103**
	Soft tissue disorders	17524	581	**1.37**	**1.16**	**1.62**	**0.0002**
**XIV**	**Diseases of the genitourinary system**	16076	1311	1.10	0.99	1.23	0.0879
**XV**	**Pregnancy complications: hypertension in pre**	13155	115	**0.57**	**0.37**	**0.87**	**0.0093**
**XVIII**	Digestive and abdominal symptoms	17524	310	**1.37**	**1.09**	**1.72**	**0.0068**
**XIX**	**Poisoning**	17524	70	1.41	0.88	2.26	0.1581
**XX**	**Self-harm**	13997	61	1.35	0.81	2.25	0.2557

		Exposure: Change in neighbourhood characteristic					
		**Increase in income**					
**ICD-chapter**		Total	Cases	HazardRatio	Lower CI	Upper CI	ProbChiSq
	**Death**	21556	425	**0.64**	**0.51**	**0.81**	**0.0001**
**IV**	**Endocrine diseases**	21102	930	**0.79**	**0.69**	**0.91**	**0.0014**
	Diabetes	21247	723	**0.75**	**0.64**	**0.89**	**0.0008**
	Obesity	21556	89	**0.56**	**0.34**	**0.91**	**0.0184**
**V**	**Mental and behavioural disorders**	21112	410	**0.66**	**0.53**	**0.82**	**0.0002**
	Disorders due to substance abuse	21556	130	**0.56**	**0.37**	**0.85**	**0.006**
	Psychotic disorders	21346	178	**0.47**	**0.32**	**0.67**	**<.0001**
	Mood disorders	21343	236	**0.62**	**0.46**	**0.83**	**0.0014**
**VI**	**Diseases of the nervous system**	21011	806	0.88	0.76	1.02	0.0794
	Sleep disorders	21556	412	**0.66**	**0.53**	**0.82**	**0.0002**
**IX**	**Diseases of the circulatory system**	20652	1202	1.09	0.97	1.23	0.1615
	Hypertension	20689	642	**0.78**	**0.66**	**0.93**	**0.0043**
	Ischemic heart diseases	21444	282	0.99	0.77	1.27	0.9164
	Angina pectoris	21485	97	0.96	0.62	1.50	0.8587
	Heart failure	21525	70	0.59	0.33	1.05	0.071
	Cerebrovascular diseases	21504	188	1.15	0.85	1.56	0.362
	Stroke	21520	148	1.08	0.77	1.53	0.6551
	Intracerebral haemorrhage	21553	23	0.78	0.30	2.01	0.6097
**X**	**Diseases of the respiratory system**	19786	1149	0.95	0.84	1.07	0.3708
	Chronic obstructive bronchitis	21540	72	0.84	0.49	1.41	0.4992
**XI**	**Diseases of the digestive system**	20066	1683	0.98	0.88	1.08	0.6426
**XII**	**Diseases of the skin**	21306	197	0.80	0.59	1.08	0.143
**XIII**	**Diseases of the musculoskeletal system**	19465	2043	0.95	0.87	1.04	0.2609
	Osteoarthritis	21556	683	0.90	0.76	1.06	0.1912
	Soft tissue disorders	21556	742	0.90	0.77	1.05	0.1599
**XIV**	**Diseases of the genitourinary system**	19778	1659	1.04	0.94	1.15	0.4921
**XV**	**Pregnancy complications: hypertension in pre**	16120	169	1.12	0.82	1.53	0.4707
**XVIII**	Digestive and abdominal symptoms	21556	443	0.87	0.72	1.06	0.1759
**XIX**	**Poisoning**	21556	117	0.79	0.53	1.17	0.2405
**XX**	**Self-harm**	16143	91	0.75	0.48	1.18	0.2135
		**Decrease in income**					
	**Death**	13174	253	**1.46**	**1.13**	**1.88**	**0.004**
**IV**	**Endocrine diseases**	12918	591	**1.26**	**1.07**	**1.48**	**0.0062**
	Diabetes	13007	460	**1.44**	**1.19**	**1.74**	**0.0002**
	Obesity	13174	48	1.16	0.66	2.06	0.6079
**V**	**Mental and behavioural disorders**	12924	188	**1.36**	**1.01**	**1.81**	**0.0408**
	Disorders due to substance abuse	13174	71	**2.10**	**1.28**	**3.45**	**0.0035**
	Psychotic disorders	13093	91	1.34	0.88	2.03	0.1748
	Mood disorders	13047	104	1.35	0.91	1.99	0.1367
**VI**	**Diseases of the nervous system**	12799	544	1.00	0.84	1.19	0.9836
	Sleep disorders	13174	286	1.08	0.85	1.37	0.5198
**IX**	**Diseases of the circulatory system**	12437	781	**1.17**	**1.01**	**1.34**	**0.0359**
	Hypertension	12533	487	1.15	0.96	1.38	0.1228
	Ischemic heart diseases	13094	212	**1.41**	**1.07**	**1.87**	**0.0148**
	Angina pectoris	13129	94	1.26	0.83	1.91	0.2743
	Heart failure	13158	27	1.42	0.65	3.09	0.3807
	Cerebrovascular diseases	13133	122	1.26	0.87	1.80	0.2179
	Stroke	13142	106	1.18	0.80	1.74	0.4063
	Intracerebral haemorrhage	13165	22	1.88	0.78	4.54	0.1581
**X**	**Diseases of the respiratory system**	12142	677	**1.21**	**1.04**	**1.41**	**0.0139**
	Chronic obstructive bronchitis	13166	34	1.39	0.69	2.78	0.3528
**XI**	**Diseases of the digestive system**	12268	1063	1.05	0.93	1.19	0.4414
**XII**	**Diseases of the skin**	13034	101	**1.86**	**1.24**	**2.78**	**0.0028**
**XIII**	**Diseases of the musculoskeletal system**	11798	1448	**1.14**	**1.02**	**1.26**	**0.0165**
	Osteoarthritis	13174	496	1.09	0.91	1.31	0.3352
	Soft tissue disorders	13174	574	1.05	0.89	1.24	0.5759
**XIV**	**Diseases of the genitourinary system**	11980	1070	0.99	0.88	1.12	0.9277
**XV**	**Pregnancy complications: hypertension in pre**	9962	61	0.83	0.49	1.40	0.483
**XVIII**	Digestive and abdominal symptoms	13174	238	1.29	1.00	1.67	0.0529
**XIX**	**Poisoning**	13174	51	**1.96**	**1.09**	**3.51**	**0.0236**
**XX**	**Self-harm**	10586	48	**2.40**	**1.31**	**4.40**	**0.0048**

ICD-chapter		Total	Cases	HazardRatio	Lower CI	Upper CI	ProbChiSq
		**Exposure: Change in neighbourhood characteristic**					
		**Decrease in unemployment**					
	**Death**	20090	409	**0.75**	**0.60**	**0.93**	**0.0075**
**IV**	**Endocrine diseases**	19664	899	0.93	0.81	1.06	0.2723
	Diabetes	19786	706	0.94	0.80	1.10	0.4117
	Obesity	20090	88	0.72	0.46	1.13	0.1527
**V**	**Mental and behavioural disorders**	19658	379	**0.77**	**0.62**	**0.96**	**0.0206**
	Disorders due to substance abuse	20090	124	**0.60**	**0.40**	**0.90**	**0.0137**
	Psychotic disorders	19898	170	**0.63**	**0.45**	**0.88**	**0.0071**
	Mood disorders	19880	224	**0.70**	**0.52**	**0.93**	**0.0148**
**VI**	**Diseases of the nervous system**	19557	785	**0.86**	**0.74**	**0.99**	**0.0397**
	Sleep disorders	20090	426	**0.70**	**0.57**	**0.87**	**0.0009**
**IX**	**Diseases of the circulatory system**	19145	1159	0.92	0.81	1.04	0.1651
	Hypertension	19202	688	**0.77**	**0.65**	**0.90**	**0.0014**
	Ischemic heart diseases	19974	287	0.92	0.72	1.18	0.5059
	Angina pectoris	20016	107	0.76	0.50	1.15	0.1925
	Heart failure	20058	57	0.69	0.38	1.25	0.2186
	Cerebrovascular diseases	20032	173	**0.72**	**0.52**	**1.00**	**0.0496**
	Stroke	20046	134	**0.65**	**0.44**	**0.96**	**0.0293**
	Intracerebral haemorrhage	20083	20	0.56	0.20	1.55	0.2646
**X**	**Diseases of the respiratory system**	18444	1125	0.93	0.82	1.05	0.2466
	Chronic obstructive bronchitis	20071	68	0.83	0.50	1.39	0.4828
**XI**	**Diseases of the digestive system**	18657	1607	**0.90**	**0.81**	**0.99**	**0.0332**
**XII**	**Diseases of the skin**	19832	204	0.85	0.64	1.13	0.2672
**XIII**	**Diseases of the musculoskeletal system**	18030	2039	0.96	0.88	1.05	0.364
	Osteoarthritis	20090	702	0.90	0.77	1.05	0.1664
	Soft tissue disorders	20090	775	**0.86**	**0.74**	**0.99**	**0.0412**
**XIV**	**Diseases of the genitourinary system**	18380	1612	0.97	0.87	1.07	0.503
**XV**	**Pregnancy complications: hypertension in pre**	15028	142	**1.47**	**1.06**	**2.05**	**0.0223**
**XVIII**	Digestive and abdominal symptoms	20090	435	**0.73**	**0.60**	**0.90**	**0.0027**
**XIX**	**Poisoning**	20090	115	**0.60**	**0.39**	**0.91**	**0.0152**
**XX**	**Self-harm**	15232	94	0.65	0.42	1.02	0.0601
		**Increase in unemployment**					
	**Death**	15213	283	1.08	0.85	1.37	0.5198
**IV**	**Endocrine diseases**	14919	642	**1.20**	**1.03**	**1.40**	**0.0233**
	Diabetes	15031	493	1.17	0.98	1.39	0.0931
	Obesity	15213	52	1.40	0.81	2.41	0.2328
**V**	**Mental and behavioural disorders**	14941	230	1.27	0.98	1.65	0.0706
	Disorders due to substance abuse	15213	78	**2.28**	**1.44**	**3.62**	**0.0005**
	Psychotic disorders	15108	103	0.92	0.62	1.37	0.6909
	Mood disorders	15078	123	0.97	0.67	1.39	0.8621
**VI**	**Diseases of the nervous system**	14816	588	**1.21**	**1.03**	**1.43**	**0.0211**
	Sleep disorders	15213	280	1.16	0.92	1.47	0.2212
**IX**	**Diseases of the circulatory system**	14490	857	**1.20**	**1.05**	**1.37**	**0.0085**
	Hypertension	14568	463	**1.25**	**1.04**	**1.50**	**0.0192**
	Ischemic heart diseases	15133	214	**1.63**	**1.24**	**2.14**	**0.0005**
	Angina pectoris	15168	87	1.10	0.72	1.68	0.6744
	Heart failure	15198	41	1.63	0.88	3.05	0.1221
	Cerebrovascular diseases	15176	140	1.17	0.83	1.63	0.369
	Stroke	15187	122	1.16	0.81	1.66	0.4217
	Intracerebral haemorrhage	15208	25	1.86	0.83	4.17	0.1324
**X**	**Diseases of the respiratory system**	14025	730	1.11	0.95	1.28	0.1825
	Chronic obstructive bronchitis	15208	42	1.35	0.73	2.49	0.3366
**XI**	**Diseases of the digestive system**	14200	1190	1.04	0.92	1.16	0.5421
**XII**	**Diseases of the skin**	15073	95	**1.88**	**1.25**	**2.82**	**0.0025**
**XIII**	**Diseases of the musculoskeletal system**	13748	1513	1.10	0.99	1.21	0.0831
	Osteoarthritis	15213	494	1.13	0.94	1.35	0.1937
	Soft tissue disorders	15213	569	**1.23**	**1.04**	**1.45**	**0.0137**
**XIV**	**Diseases of the genitourinary system**	13907	1167	1.03	0.92	1.16	0.6106
**XV**	**Pregnancy complications: hypertension in pre**	11451	96	0.72	0.47	1.10	0.1327
**XVIII**	Digestive and abdominal symptoms	15213	259	1.24	0.97	1.59	0.0861
**XIX**	**Poisoning**	15213	58	1.61	0.96	2.71	0.0726
**XX**	**Self-harm**	11903	48	1.45	0.82	2.57	0.2029

ICD-chapter		Total	Cases	HazardRatio	Lower CI	Upper CI	ProbChiSq
		**Exposure: Change in neighbourhood characteristic**					
		**Increase in green space**					
	**Death**	23006	472	**0.71**	**0.58**	**0.87**	**0.0009**
**IV**	**Endocrine diseases**	22535	969	1.07	0.94	1.22	0.3307
	Diabetes	22692	746	1.03	0.88	1.20	0.7034
	Obesity	23006	86	0.81	0.51	1.28	0.3604
**V**	**Mental and behavioural disorders**	22535	429	0.88	0.72	1.08	0.2116
	Disorders due to substance abuse	23006	136	1.06	0.75	1.50	0.7385
	Psychotic disorders	22796	188	**0.50**	**0.36**	**0.70**	**<.0001**
	Mood disorders	22777	247	**0.68**	**0.52**	**0.89**	**0.0058**
**VI**	**Diseases of the nervous system**	22432	887	1.01	0.88	1.16	0.8971
	Sleep disorders	23006	455	1.06	0.87	1.29	0.5444
**IX**	**Diseases of the circulatory system**	21984	1311	1.10	0.98	1.23	0.1083
	Hypertension	22061	727	1.05	0.90	1.22	0.5324
	Ischemic heart diseases	22883	332	1.11	0.88	1.39	0.3771
	Angina pectoris	22929	130	1.25	0.87	1.79	0.2233
	Heart failure	22980	65	0.72	0.42	1.25	0.2465
	Cerebrovascular diseases	22954	207	1.04	0.78	1.38	0.8168
	Stroke	22969	163	0.90	0.65	1.26	0.5326
	Intracerebral haemorrhage	23003	25	0.60	0.25	1.48	0.2674
**X**	**Diseases of the respiratory system**	21219	1289	1.03	0.92	1.16	0.5756
	Chronic obstructive bronchitis	22987	73	1.01	0.62	1.65	0.976
**XI**	**Diseases of the digestive system**	21499	1864	1.03	0.93	1.13	0.5957
**XII**	**Diseases of the skin**	22763	193	1.15	0.86	1.54	0.3473
**XIII**	**Diseases of the musculoskeletal system**	20783	2273	1.06	0.97	1.16	0.1894
	Osteoarthritis	23006	751	0.95	0.82	1.11	0.5293
	Soft tissue disorders	23006	891	1.12	0.98	1.29	0.1034
**XIV**	**Diseases of the genitourinary system**	21091	1837	**1.15**	**1.05**	**1.27**	**0.0037**
**XV**	**Pregnancy complications: hypertension in pre**	17272	185	0.90	0.67	1.22	0.5093
**XVIII**	Digestive and abdominal symptoms	23006	475	**1.25**	**1.04**	**1.51**	**0.0164**
**XIX**	**Poisoning**	23006	127	0.92	0.64	1.33	0.6627
**XX**	**Self-harm**	17249	97	0.78	0.50	1.22	0.2741
		**Decrease in green space**					
	**Death**	16340	301	1.12	0.88	1.41	0.3564
**IV**	**Endocrine diseases**	16002	749	0.95	0.82	1.10	0.5203
	Diabetes	16112	585	0.95	0.81	1.12	0.5391
	Obesity	16340	69	1.26	0.77	2.04	0.3569
**V**	**Mental and behavioural disorders**	16019	264	1.03	0.80	1.31	0.8468
	Disorders due to substance abuse	16340	106	1.00	0.68	1.49	0.9865
	Psychotic disorders	16216	111	1.18	0.80	1.73	0.3988
	Mood disorders	16185	140	1.19	0.84	1.67	0.3263
**VI**	**Diseases of the nervous system**	15855	681	1.02	0.88	1.19	0.7915
	Sleep disorders	16340	339	**0.78**	**0.63**	**0.97**	**0.0234**
**IX**	**Diseases of the circulatory system**	15499	941	1.05	0.92	1.19	0.4838
	Hypertension	15549	559	0.93	0.79	1.10	0.4136
	Ischemic heart diseases	16243	239	0.87	0.67	1.13	0.2937
	Angina pectoris	16281	95	0.80	0.53	1.21	0.2845
	Heart failure	16317	41	1.07	0.57	2.01	0.8434
	Cerebrovascular diseases	16282	139	0.93	0.67	1.31	0.6903
	Stroke	16295	116	0.96	0.66	1.39	0.8078
	Intracerebral haemorrhage	16329	25	1.18	0.53	2.64	0.6906
**X**	**Diseases of the respiratory system**	14978	777	0.95	0.82	1.10	0.4889
	Chronic obstructive bronchitis	16332	54	0.71	0.41	1.22	0.2157
**XI**	**Diseases of the digestive system**	15118	1305	0.91	0.81	1.01	0.0817
**XII**	**Diseases of the skin**	16129	147	1.04	0.75	1.45	0.8205
**XIII**	**Diseases of the musculoskeletal system**	14578	1751	0.99	0.90	1.09	0.7714
	Osteoarthritis	16340	609	0.86	0.73	1.01	0.0609
	Soft tissue disorders	16340	640	0.93	0.79	1.08	0.3344
**XIV**	**Diseases of the genitourinary system**	14851	1277	0.90	0.80	1.00	0.0562
**XV**	**Pregnancy complications: hypertension in pre**	12162	95	0.90	0.59	1.35	0.5975
**XVIII**	Digestive and abdominal symptoms	16340	320	**0.77**	**0.61**	**0.96**	**0.0214**
**XIX**	**Poisoning**	16340	66	1.05	0.64	1.72	0.843
**XX**	**Self-harm**	12496	57	1.35	0.79	2.30	0.2755

eTable 12. Baseline characteristics of the study population in 8 non-randomised modifications to neighbourhood characteristics in participants who did move residence. Figures are numbers unless otherwise stated.

	1. Favourable change in neighbourhood education		2. Favourable change in neighbourhood income		3. Favourable change in neighbourhood unemployment		4. Favourable change in green space	
Characteristics	Experimental group	Reference group	Experimental group	Reference group	Experimental group	Reference group	Experimental group	Reference group
Number of participants	4850	27159	2661	24293	7599	21744	4564	27490
Mean age (SD)	47.2 (9.3)	48.6 (9.5)	45.0 (9.8)	48.5 (9.9)	47.6 (9.5)	48.5 (9.6)	46.5 (9.6)	48.4 (9.6)
Sex, N (%)								
Men	1176 (24.3)	6367 (23.4)	643 (24.2)	5654 (23.3)	1798 (23.7)	5210 (24.0)	1131 (24.8)	6164 (22.4)
Women	3674 (75.8)	20792 (76.6)	2018 (75.8)	18639 (76.7)	5801 (76.3)	16534 (76.0)	3433 (75.2)	21326 (77.6)
Participant's education, N (%)								
Primary	548 (11.3)	4996 (18.4)	257 (9.7)	3914 (16.1)	816 (10.8)	3537 (16.3)	551 (12.1)	3848 (14.0)
Secondary	1796 (37.1)	12241 (45.2)	952 (35.9)	9888 (40.8)	2806 (37.0)	9177 (42.3)	1757 (38.6)	10331 (37.6)
Tertiary	2500 (51.6)	9871 (36.4)	1445 (54.5)	10460 (43.1)	3965 (52.3)	9001 (41.5)	2246 (49.3)	13287 (48.4)
Cohort, N (%)								
HeSSup	708 (14.6)	5834 (21.5)	464 (17.4)	4492 (18.5)	1363 (17.9)	3897 (17.9)	962 (21.1)	4205 (15.3)
FPS	4142 (85.4)	21325 (78.5)	2197 (82.6)	19801 (81.5)	6236 (82.1)	17847 (82.1)	3602 (78.9)	23285 (84.7)
Neighbourhood characteristics at baseline and follow-up								
Disadvantaged-advantaged	4850	0	2661	0	7599	0	4564	0
Disadvantaged- disadvantaged	0	27159	0	24293	0	21744	0	27490

	5. Unfavourable change in neighbourhood education		6. Unfavourable change in neighbourhood income		7. Unfavourable change in neighbourhood unemployment		8. Unfavourable change in green space	
Characteristics	Experimental group	Reference group	Experimental group	Reference group	Experimental group	Reference group	Experimental group	Reference group
Number of participants	6257	27358	4921	31217	9570	25096	5123	34424
Mean age (SD)	48.1 (9.4)	48.4 (9.0)	48.1 (9.7)	48.7 (8.6)	48.3 (9.3)	48.6 (8.9)	48.0 (9.6)	48.3 (8.9)
Sex, N (%)								
Men	1449 (23.2)	6266 (22.9)	1120 (22.8)	7157 (22.9)	2257 (23.6)	5570 (22.2)	1184 (23.1)	8481 (24.6)
Women	4808 (76.8)	21092 (77.1)	3801 (77.2)	24060 (77.1)	7313 (76.4)	19526 (77.8)	3939 (76.9)	25943 (75.4)
Participant's education, N (%)								
Primary	757 (12.1)	2174 (8.0)	739 (15.0)	3205 (10.3)	1278 (13.4)	2591 (10.3)	635 (12.4)	4514 (13.1)
Secondary	2507 (40.1)	8050 (29.4)	1947 (39.6)	10729 (34.4)	3665 (38.3)	8243 (32.9)	1854 (36.2)	13273 (38.6)
Tertiary	2985 (47.8)	17119 (62.6)	2230 (45.4)	17260 (55.3)	4616 (48.3)	14245 (56.8)	2630 (51.4)	16575 (48.2)
Cohort, N (%)								
HeSSup	1019 (16.3)	3444 (12.6)	856 (17.4)	4464 (14.3)	1474 (15.4)	3739 (14.9)	828 (16.2)	7336 (21.3)
FPS	5238 (83.7)	23914 (87.4)	4065 (82.6)	26753 (85.7)	8096 (84.6)	21357 (85.1)	4295 (83.8)	27088 (78.7)
Neighbourhood characteristics at baseline and follow-up								
Advantaged-disadvantaged	6257	0	4921	0	9570	0	5123	0
Advantaged- advantaged	0	27358	0	31217	0	25096	0	34424

*Only participants who did not move residental address were included in analyses emulating 'natural experiments'.

eFigure 5. Association of change in neighbourhood characteristics with health outcomes among participants with no change in residential address or employment

Population	Change in neighbourhood	Health outcome	N (total)	N (cases)	Hazard ratio (95% CI)	P-value
**Non-movers who also had stable employment**	**From unfavourable to favourable**					
	Neighbourhood unemployment	Diabetes	23,660	1521	0.88 (0.77-1.00)	0.06
		Diseases of the skin	23,912	203	0.40 (0.20-0.77)	0.007
	Neighbourhood income	Stroke	21,952	266	0.85 (0.75-0.96)	0.01
	Neighbourhood education	Osteoarthrtis	26,247	1832	0.73 (0.52-1.03)	0.08
	Neighbourhood green space	Diabetes	26,859	886	0.80 (0.64-0.99)	0.04
	**From favourable to unfavourable**					
	Neighbourhood unemployment	Diabetes	29,528	1629	1.22 (1.08-1.37)	0.001
		Diseases of the skin	29,743	246	1.26 (0.93-1.71)	0.14
	Neighbourhood income	Stroke	31,237	301	1.16 (1.04-1.29)	0.006
	Neighbourhood education	Osteoarthrtis	29,143	1633	1.48 (1.14-1.93)	0.003
	Neighbourhood green space	Diabetes	32,687	1792	1.15 (1.01-1.31)	0.03

Hazard ratio for health outcome

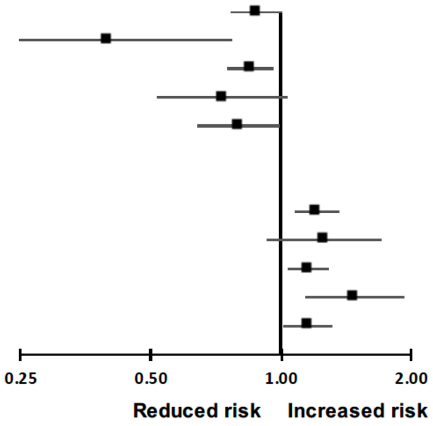

Hazard ratios are adjusted for age, sex, education and cohort.

eTable 13. Association of change in neighbourhood characteristics with subsequent health outcomes in non-movers by sex

Population	Exposure	Health outcome	N (total)	N (cases)	Hazard ratio (95% CI)			P-value
**Non-moving men**	**Neighbourhood unemployment**							
	From high to low vs stable high	Diabetes	28639	1911	0.73	0.60	0.90	0.0023
		Diseases of the skin	29015	282	0.49	0.26	0.93	0.0298
	From low to high vs stable low	Diabetes	34039	1913	1.15	0.97	1.37	0.1111
		Diseases of the skin	34336	294	1.11	0.68	1.80	0.6837
	**Neighbourhood income**							
	From low to high vs stable low	Stroke	35947	455	0.53	0.23	1.20	0.125
	From high to low vs stable high	Stroke	35982	384	1.40	0.89	2.23	0.1497
	**Neighbourhood education**							
	From low to high vs stable low	Osteoarthrtis	31952	2248	0.89	0.68	1.16	0.3795
	From high to low vs stable high	Osteoarthrtis	33592	1909	1.03	0.81	1.31	0.8047
	**Neighbourhood greennes**							
	From low to high vs stable low	Diabetes	31339	1996	0.88	0.70	1.12	0.3044
	From high to low vs stable high	Diabetes	38738	2190	1.14	0.92	1.40	0.2358
**Non-moving women**	**Neighbourhood unemployment**							
	From high to low vs stable high	Diabetes	23660	1521	0.88	0.78	1.01	0.0649
		Diseases of the skin	23912	203	0.81	0.58	1.14	0.2231
	From low to high vs stable low	Diabetes	29528	1629	1.12	0.99	1.26	0.0642
		Diseases of the skin	29743	246	1.35	1.02	1.79	0.0357
	**Neighbourhood income**							
	From low to high vs stable low	Stroke	21952	266	0.47	0.24	0.92	0.0271
	From high to low vs stable high	Stroke	31237	301	1.36	0.99	1.87	0.0573
	**Neighbourhood education**							
	From low to high vs stable low	Osteoarthrtis	26247	1832	0.86	0.75	0.99	0.0417
	From high to low vs stable high	Osteoarthrtis	29143	1633	1.21	1.07	1.37	0.0020
	**Neighbourhood greennes**							
	From low to high vs stable low	Diabetes	24311	1409	0.83	0.70	0.98	0.0263
	From high to low vs stable high	Diabetes	29396	1448	1.13	0.98	1.31	0.0862

*Hazard ratios are adjusted for age, sex, education and cohort.

eTable 14. Association of change in neighbourhood characteristics with subsequent health outcomes in non-movers who had more than one lifestyle risk factors and lived in urban areas

Population	Exposure	Health outcome	N (total)	N (cases)	Hazard ratio (95% CI)			P-value
Non-movers, ≥1 behavioral risks	**Neighbourhood unemployment**							
	From high to low vs stable high	Diabetes	28639	1911	0.87	0.77	0.98	0.027
		Diseases of the skin	29015	282	0.62	0.42	0.92	0.0182
	From low to high vs stable low	Diabetes	34039	1913	1.06	0.95	1.19	0.2902
		Diseases of the skin	34336	294	1.34	0.99	1.82	0.057
	**Neighbourhood income**							
	From low to high vs stable low	Stroke	35947	455	0.39	0.19	0.78	0.0082
	From high to low vs stable high	Stroke	35982	384	1.03	0.71	1.49	0.8687
	**Neighbourhood education**							
	From low to high vs stable low	Osteoarthrtis	31952	2248	0.94	0.80	1.10	0.4412
	From high to low vs stable high	Osteoarthrtis	33592	1909	1.19	1.03	1.37	0.0164
	**Neighbourhood greennes**							
	From low to high vs stable low	Diabetes	31339	1996	0.92	0.79	1.07	0.2883
	From high to low vs stable high	Diabetes	38738	2190	1.20	1.04	1.37	0.0104
Non-movers in urban areas	**Neighbourhood unemployment**							
	From high to low vs stable high	Diabetes	23660	1521	0.83	0.74	0.93	0.0015
		Diseases of the skin	23912	203	0.79	0.58	1.08	0.1330
	From low to high vs stable low	Diabetes	29528	1629	1.11	1.00	1.23	0.0485
		Diseases of the skin	29743	246	1.25	0.96	1.62	0.1009
	**Neighbourhood income**							
	From low to high vs stable low	Stroke	21952	266	0.49	0.28	0.86	0.0124
	From high to low vs stable high	Stroke	31237	301	1.32	0.99	1.75	0.0547
	**Neighbourhood education**							
	From low to high vs stable low	Osteoarthrtis	26247	1832	0.86	0.75	0.98	0.0247
	From high to low vs stable high	Osteoarthrtis	29143	1633	1.14	1.01	1.28	0.0308
	**Neighbourhood greennes**							
	From low to high vs stable low	Diabetes	29018	1874	0.86	0.74	1.01	0.0597
	From high to low vs stable high	Diabetes	29676	1745	1.12	0.99	1.27	0.0754

*Hazard ratios are adjusted for age, sex, education and cohort.

eTable 15. Association of change in neighbourhood characteristics with subsequent health outcomes in non-movers after multivariable adjustments.

		FPS only											
		Base model: adjusted for age, sex and education						Further adjustment for marital status, occupational status, type of residence, number of rooms, floor area, neighbourhood population density, place of residence (urban vs rural)					
Exposure	Health outcome	N (total)	N(cases)	Hazard Ratio	LowerCI	UpperCI	ProbChiSq	N (total)	N(cases)	Hazard Ratio	LowerCI	UpperCI	ProbChi Sq
**Neighbourhood education**													
From disadvantaged to advantaged	Osteoarthrtis	25644	1932	0.87	0.76	0.99	0.04	23442	1836	0.86	0.75	0.98	0.02
From advantaged to disadvantaged	Osteoarthrtis	29152	1735	1.19	1.06	1.33	0.003	26926	1645	1.19	1.05	1.34	0.005
**Neighbourhood income**													
From disadvantaged to advantaged	Stroke	21933	298	0.55	0.31	0.95	0.03	20213	287	0.53	0.29	0.97	0.04
From advantaged to disadvantaged	Stroke	30706	338	1.33	1.00	1.76	0.05	28408	312	1.23	0.87	1.72	0.24
**Neighbourhood unemployment**													
From disadvantaged to advantaged	Diabetes	23530	1624	0.87	0.77	0.97	0.02	21635	1538	0.93	0.82	1.05	0.24
	Diseases of the skin	23837	224	0.73	0.52	1.02	0.06	21926	206	0.64	0.44	0.91	0.01
From advantaged to disadvantaged	Diabetes	28956	1710	1.11	1.00	1.23	0.05	26807	1622	1.09	0.98	1.21	0.12
	Diseases of the skin	29183	249	1.39	1.07	1.81	0.01	27017	237	1.32	1.01	1.73	0.04
**Neighbourhood green space**													
From disadvantaged to advantaged	Diabetes	26312	1747	0.82	0.70	0.95	0.01	24377	1663	0.86	0.73	1.01	0.07
From advantaged to disadvantaged	Diabetes	30807	1821	1.16	1.03	1.32	0.02	28143	1711	1.2	1.04	1.37	0.01

eTable 16. Association of change in 750m x 750m grid neighbourhood characteristics with subsequent health outcomes in non-movers

ICD-chapter		Total	Cases	HazardRatio	Lower CI	Upper CI	ProbChiSq
		**Exposure: Change in neighbourhood characteristic**					
		**Increase in education**					
	**Death**	32233	1313	**0.822**	**0.679**	**0.994**	**0.043**
IV	**Endocrine diseases**	31194	2514	0.993	0.877	1.125	0.9159
	Diabetes	31507	2131	1.017	0.889	1.163	0.811
	Obesity	32233	131	0.75	0.422	1.334	0.3276
V	**Mental and behavioural disorders**	31761	582	0.835	0.633	1.101	0.2005
	Disorders due to substance abuse	32233	234	0.802	0.512	1.259	0.3382
	Psychotic disorders	31909	186	**0.533**	**0.296**	**0.958**	**0.0355**
	Mood disorders	32009	246	1.189	0.826	1.712	0.3514
VI	**Diseases of the nervous system**	30956	1759	0.994	0.856	1.154	0.9382
	Sleep disorders	32233	984	0.998	0.824	1.208	0.9837
IX	**Diseases of the circulatory system**	29647	2862	1.109	0.991	1.242	0.0722
	Hypertension	28851	1653	0.865	0.736	1.017	0.0789
	Ischemic heart diseases	31763	949	1.07	0.876	1.307	0.5075
	Angina pectoris	31918	391	1.185	0.877	1.6	0.2689
	Heart failure	32164	221	0.929	0.597	1.444	0.7421
	Cerebrovascular diseases	32071	489	1.058	0.797	1.405	0.697
	Stroke	32101	420	1.024	0.751	1.397	0.8786
	Intracerebral haemorrhage	32216	64	1.719	0.895	3.302	0.1041
X	**Diseases of the respiratory system**	30344	1742	0.922	0.792	1.074	0.2968
	Chronic obstructive bronchitis	32188	192	0.855	0.525	1.39	0.5268
XI	**Diseases of the digestive system**	29526	2963	**0.882**	**0.783**	**0.994**	**0.039**
XII	**Diseases of the skin**	31926	305	0.691	0.458	1.041	0.0773
XIII	**Diseases of the musculoskeletal system**	27530	4268	0.999	0.91	1.098	0.9905
	Osteoarthritis	32233	2268	0.971	0.85	1.109	0.6636
	Soft tissue disorders	32233	1702	1.119	0.969	1.292	0.1263
XIV	**Diseases of the genitourinary system**	28750	2800	1.064	0.952	1.191	0.2746
XV	**Pregnancy complications: hypertension in pre**	24530	28	0.934	0.323	2.707	0.9004
XVIII	Digestive and abdominal symptoms	32233	651	0.797	0.612	1.039	0.0932
XIX	**Poisoning**	32233	117	0.703	0.367	1.347	0.2878
XX	**Self-harm**	25484	101	0.929	0.507	1.701	0.8111
		**Decrease in education**					
	**Death**	33311	1103	0.986	0.839	1.16	0.8682
IV	**Endocrine diseases**	32453	2195	**1.144**	**1.025**	**1.276**	**0.016**
	Diabetes	32734	1761	**1.16**	**1.027**	**1.309**	**0.0168**
	Obesity	33311	97	**1.621**	**1.013**	**2.595**	**0.0442**
V	**Mental and behavioural disorders**	32908	529	1.101	0.881	1.376	0.3972
	Disorders due to substance abuse	33311	163	1.204	0.82	1.768	0.3438
	Psychotic disorders	33072	174	0.834	0.545	1.276	0.4037
	Mood disorders	33110	259	1.062	0.769	1.467	0.714
VI	**Diseases of the nervous system**	32121	1654	1.003	0.88	1.144	0.9595
	Sleep disorders	33311	849	1.191	0.998	1.421	0.0531
IX	**Diseases of the circulatory system**	30919	2663	1.064	0.96	1.179	0.2354
	Hypertension	30479	1700	0.993	0.87	1.133	0.9167
	Ischemic heart diseases	32902	861	0.914	0.756	1.105	0.3523
	Angina pectoris	33030	344	0.787	0.574	1.077	0.1349
	Heart failure	33264	172	0.84	0.545	1.295	0.4291
	Cerebrovascular diseases	33186	440	1.007	0.78	1.3	0.9577
	Stroke	33223	370	1.008	0.764	1.331	0.9539
	Intracerebral haemorrhage	33303	67	1.549	0.869	2.76	0.1378
X	**Diseases of the respiratory system**	31349	1561	0.989	0.862	1.133	0.8684
	Chronic obstructive bronchitis	33287	143	**1.563**	**1.061**	**2.302**	**0.0238**
XI	**Diseases of the digestive system**	30793	2959	1.026	0.93	1.133	0.6049
XII	**Diseases of the skin**	33012	287	0.711	0.499	1.014	0.0598
XIII	**Diseases of the musculoskeletal system**	29103	4091	0.992	0.911	1.08	0.8475
	Osteoarthritis	33311	1889	1.1	0.976	1.24	0.1183
	Soft tissue disorders	33311	1473	1.113	0.972	1.273	0.1214
XIV	**Diseases of the genitourinary system**	29978	2832	0.996	0.899	1.103	0.9328
XV	**Pregnancy complications: hypertension in pre**	25781	33	1.082	0.466	2.509	0.8545
XVIII	Digestive and abdominal symptoms	33311	528	0.982	0.777	1.241	0.8791
XIX	**Poisoning**	33311	109	0.977	0.588	1.623	0.9282
XX	**Self-harm**	29134	99	0.763	0.425	1.37	0.3656

ICD-chapter		Total	Cases	HazardRatio	Lower CI	Upper CI	ProbChiSq
		**Exposure: Change in neighbourhood characteristic**					
		**Increase in income**					
	**Death**	27758	1173	0.787	0.6	1.033	0.0842
**IV**	**Endocrine diseases**	26887	2140	0.938	0.787	1.117	0.4731
	Diabetes	27132	1796	0.952	0.785	1.153	0.6127
	Obesity	27758	96	0.962	0.464	1.994	0.9162
**V**	**Mental and behavioural disorders**	27313	542	0.807	0.566	1.149	0.2343
	Disorders due to substance abuse	27758	198	0.72	0.38	1.363	0.3127
	Psychotic disorders	27445	176	0.569	0.279	1.16	0.1206
	Mood disorders	27552	253	0.862	0.532	1.396	0.5461
**VI**	**Diseases of the nervous system**	26706	1489	**1.204**	**1.001**	**1.449**	**0.0492**
	Sleep disorders	27758	828	0.986	0.756	1.287	0.9186
**IX**	**Diseases of the circulatory system**	25662	2459	0.953	0.81	1.122	0.5649
	Hypertension	24964	1407	0.859	0.691	1.067	0.1686
	Ischemic heart diseases	27391	826	1.031	0.777	1.366	0.8342
	Angina pectoris	27527	321	1.21	0.79	1.853	0.3808
	Heart failure	27711	189	1.019	0.553	1.878	0.9508
	Cerebrovascular diseases	27636	448	0.853	0.56	1.3	0.4605
	Stroke	27665	388	0.817	0.515	1.298	0.3928
	Intracerebral haemorrhage	27745	76	0.865	0.315	2.375	0.7786
**X**	**Diseases of the respiratory system**	26116	1514	1.003	0.828	1.214	0.9787
	Chronic obstructive bronchitis	27716	186	1.125	0.625	2.025	0.6945
**XI**	**Diseases of the digestive system**	25501	2505	1.003	0.862	1.167	0.9709
**XII**	**Diseases of the skin**	27496	255	0.739	0.43	1.27	0.2733
**XIII**	**Diseases of the musculoskeletal system**	23844	3465	1.021	0.899	1.16	0.7509
	Osteoarthritis	27758	1832	0.958	0.79	1.16	0.6578
	Soft tissue disorders	27758	1335	**1.265**	**1.047**	**1.528**	**0.0148**
**XIV**	**Diseases of the genitourinary system**	24886	2332	1.044	0.898	1.213	0.5775
**XV**	**Pregnancy complications: hypertension in pre**	21085	30	**2.582**	**1.147**	**5.816**	**0.022**
**XVIII**	Digestive and abdominal symptoms	27758	518	0.787	0.546	1.134	0.199
**XIX**	**Poisoning**	27758	114	0.409	0.15	1.113	0.0801
**XX**	**Self-harm**	22514	96	0.359	0.113	1.136	0.0813
		**Decrease in income**					
	**Death**	35268	1175	1.04	0.881	1.228	0.6429
**IV**	**Endocrine diseases**	34320	2425	1.092	0.974	1.224	0.1296
	Diabetes	34647	1985	1.128	0.996	1.277	0.0588
	Obesity	35268	123	**1.659**	**1.068**	**2.577**	**0.0243**
**V**	**Mental and behavioural disorders**	34881	539	1.218	0.966	1.536	0.0959
	Disorders due to substance abuse	35268	183	1.375	0.943	2.007	0.0983
	Psychotic disorders	35044	174	1.267	0.85	1.89	0.2451
	Mood disorders	35071	234	1.152	0.803	1.651	0.4425
**VI**	**Diseases of the nervous system**	33973	1775	1.004	0.875	1.152	0.953
	Sleep disorders	35268	940	1.153	0.961	1.383	0.1258
**IX**	**Diseases of the circulatory system**	32564	2854	1.041	0.935	1.159	0.4617
	Hypertension	32079	1831	1.058	0.925	1.21	0.4094
	Ischemic heart diseases	34788	917	1.04	0.86	1.257	0.6885
	Angina pectoris	34927	381	1.228	0.93	1.622	0.1481
	Heart failure	35204	192	1.386	0.956	2.01	0.0848
	Cerebrovascular diseases	35114	447	1.175	0.907	1.522	0.2209
	Stroke	35149	375	1.246	0.945	1.643	0.1195
	Intracerebral haemorrhage	35256	50	1.434	0.696	2.956	0.3283
**X**	**Diseases of the respiratory system**	33215	1660	0.98	0.849	1.132	0.787
	Chronic obstructive bronchitis	35243	141	**1.569**	**1.037**	**2.375**	**0.0332**
**XI**	**Diseases of the digestive system**	32524	3186	1.009	0.91	1.118	0.8661
**XII**	**Diseases of the skin**	34949	311	1.017	0.734	1.408	0.921
**XIII**	**Diseases of the musculoskeletal system**	30610	4549	1.016	0.932	1.107	0.7263
	Osteoarthritis	35268	2166	**1.143**	**1.015**	**1.288**	**0.028**
	Soft tissue disorders	35268	1711	0.961	0.834	1.108	0.5867
**XIV**	**Diseases of the genitourinary system**	31625	3075	0.965	0.867	1.074	0.5138
**XV**	**Pregnancy complications: hypertension in pre**	27387	26	0.72	0.244	2.128	0.5524
**XVIII**	Digestive and abdominal symptoms	35268	602	1.109	0.883	1.392	0.3728
**XIX**	**Poisoning**	35268	101	1.181	0.691	2.017	0.543
**XX**	**Self-harm**	30301	97	**1.659**	**1.013**	**2.716**	**0.0443**

ICD-chapter		Total	Cases	HazardRatio	Lower CI	Upper CI	ProbChiSq
		**Exposure: Change in neighbourhood characteristic**					
		**Decrease in unemployment**					
	**Death**	29629	1164	0.884	0.752	1.039	0.1352
**IV**	**Endocrine diseases**	28663	2296	0.938	0.838	1.049	0.2599
	Diabetes	28938	1945	0.934	0.827	1.056	0.275
	Obesity	29629	116	1.072	0.672	1.708	0.7713
**V**	**Mental and behavioural disorders**	29182	557	**0.77**	**0.604**	**0.982**	**0.0351**
	Disorders due to substance abuse	29629	201	**0.627**	**0.402**	**0.978**	**0.0394**
	Psychotic disorders	29317	182	0.894	0.596	1.339	0.5855
	Mood disorders	29421	247	0.914	0.651	1.284	0.6051
**VI**	**Diseases of the nervous system**	28432	1607	0.95	0.832	1.086	0.453
	Sleep disorders	29629	840	**0.791**	**0.652**	**0.96**	**0.0179**
**IX**	**Diseases of the circulatory system**	27318	2691	1.018	0.921	1.125	0.728
	Hypertension	26540	1608	**0.843**	**0.735**	**0.967**	**0.0144**
	Ischemic heart diseases	29188	945	0.93	0.78	1.108	0.4176
	Angina pectoris	29340	373	0.992	0.755	1.305	0.9566
	Heart failure	29577	199	0.802	0.534	1.205	0.2886
	Cerebrovascular diseases	29490	458	0.819	0.629	1.066	0.1369
	Stroke	29522	397	0.756	0.565	1.01	0.0585
	Intracerebral haemorrhage	29615	71	0.784	0.401	1.533	0.4769
**X**	**Diseases of the respiratory system**	27878	1573	0.988	0.866	1.126	0.8526
	Chronic obstructive bronchitis	29591	180	0.78	0.502	1.209	0.2662
**XI**	**Diseases of the digestive system**	27202	2765	0.913	0.824	1.01	0.0774
**XII**	**Diseases of the skin**	29340	286	0.873	0.633	1.205	0.4099
**XIII**	**Diseases of the musculoskeletal system**	25400	3861	0.987	0.907	1.073	0.7545
	Osteoarthritis	29629	2000	0.913	0.809	1.031	0.1425
	Soft tissue disorders	29629	1518	**1.171**	**1.03**	**1.331**	**0.0158**
**XIV**	**Diseases of the genitourinary system**	26545	2535	1.053	0.951	1.165	0.3218
**XV**	**Pregnancy complications: hypertension in pre**	22423	28	1.567	0.688	3.569	0.2852
**XVIII**	Digestive and abdominal symptoms	29629	564	**0.777**	**0.612**	**0.986**	**0.0379**
**XIX**	**Poisoning**	29629	123	0.936	0.579	1.514	0.7883
**XX**	**Self-harm**	24225	107	1.08	0.656	1.777	0.7626
		**Increase in unemployment**					
	**Death**	34311	1201	0.988	0.861	1.134	0.8639
**IV**	**Endocrine diseases**	33436	2322	**1.121**	**1.019**	**1.234**	**0.0191**
	Diabetes	33740	1879	1.107	0.995	1.231	0.0616
	Obesity	34311	106	**2.087**	**1.407**	**3.095**	**0.0003**
**V**	**Mental and behavioural disorders**	33912	531	1.131	0.928	1.38	0.2226
	Disorders due to substance abuse	34311	184	**1.542**	**1.127**	**2.11**	**0.0068**
	Psychotic disorders	34076	173	0.868	0.598	1.258	0.4542
	Mood disorders	34110	248	0.953	0.703	1.292	0.7548
**VI**	**Diseases of the nervous system**	33110	1705	**1.222**	**1.095**	**1.363**	**0.0003**
	Sleep disorders	34311	949	1.154	0.995	1.338	0.0589
**IX**	**Diseases of the circulatory system**	31755	2694	**1.12**	**1.025**	**1.225**	**0.0126**
	Hypertension	31348	1672	**1.208**	**1.08**	**1.35**	**0.0009**
	Ischemic heart diseases	33890	824	0.994	0.842	1.174	0.9441
	Angina pectoris	34017	340	1.04	0.805	1.344	0.7629
	Heart failure	34252	185	1.047	0.742	1.478	0.7941
	Cerebrovascular diseases	34170	444	0.936	0.743	1.179	0.5751
	Stroke	34204	373	0.975	0.76	1.25	0.8394
	Intracerebral haemorrhage	34300	58	0.679	0.333	1.384	0.2862
**X**	**Diseases of the respiratory system**	32317	1653	**1.161**	**1.037**	**1.299**	**0.0094**
	Chronic obstructive bronchitis	34281	151	**1.552**	**1.099**	**2.193**	**0.0126**
**XI**	**Diseases of the digestive system**	31651	3012	**1.168**	**1.075**	**1.27**	**0.0002**
**XII**	**Diseases of the skin**	34011	290	0.881	0.66	1.175	0.3874
**XIII**	**Diseases of the musculoskeletal system**	29849	4283	**1.083**	**1.008**	**1.163**	**0.0301**
	Osteoarthritis	34311	2057	**1.155**	**1.043**	**1.278**	**0.0055**
	Soft tissue disorders	34311	1576	1.083	0.963	1.217	0.1834
**XIV**	**Diseases of the genitourinary system**	30788	2948	1.03	0.944	1.124	0.5073
**XV**	**Pregnancy complications: hypertension in pre**	26704	28	0.529	0.183	1.532	0.2407
**XVIII**	Digestive and abdominal symptoms	34311	569	**1.233**	**1.022**	**1.488**	**0.0291**
**XIX**	**Poisoning**	34311	95	1.465	0.946	2.271	0.0871
**XX**	**Self-harm**	29310	89	1.051	0.649	1.703	0.8398

ICD-chapter		Total	Cases	HazardRatio	Lower CI	Upper CI	ProbChiSq
		**Exposure: Change in neighbourhood characteristic**					
		**Increase in green space**					
	**Death**	32393	1301	0.847	0.714	1.006	0.0588
**IV**	**Endocrine diseases**	31453	2453	**0.82**	**0.725**	**0.927**	**0.0016**
	Diabetes	31735	2035	**0.833**	**0.728**	**0.953**	**0.008**
	Obesity	32393	99	1.009	0.582	1.751	0.9736
**V**	**Mental and behavioural disorders**	31910	583	**0.736**	**0.57**	**0.95**	**0.0187**
	Disorders due to substance abuse	32393	197	**0.618**	**0.389**	**0.983**	**0.0419**
	Psychotic disorders	32060	198	0.783	0.51	1.201	0.2618
	Mood disorders	32156	271	0.711	0.488	1.034	0.0742
**VI**	**Diseases of the nervous system**	31242	1683	1.11	0.972	1.267	0.1226
	Sleep disorders	32393	954	**1.246**	**1.052**	**1.476**	**0.0111**
**IX**	**Diseases of the circulatory system**	29977	2755	1.044	0.938	1.163	0.4306
	Hypertension	29349	1602	0.964	0.835	1.112	0.6116
	Ischemic heart diseases	31966	871	1.067	0.882	1.291	0.506
	Angina pectoris	32113	321	**1.342**	**1.002**	**1.796**	**0.0483**
	Heart failure	32342	205	0.888	0.58	1.362	0.5874
	Cerebrovascular diseases	32282	504	0.774	0.584	1.025	0.0736
	Stroke	32300	435	0.808	0.599	1.088	0.1603
	Intracerebral haemorrhage	32381	79	0.629	0.289	1.37	0.2429
**X**	**Diseases of the respiratory system**	30508	1667	1.035	0.905	1.184	0.6176
	Chronic obstructive bronchitis	32351	202	0.897	0.585	1.376	0.6186
**XI**	**Diseases of the digestive system**	29812	2922	1.046	0.944	1.158	0.3894
**XII**	**Diseases of the skin**	32102	292	1.11	0.811	1.519	0.5133
**XIII**	**Diseases of the musculoskeletal system**	28054	4015	**1.19**	**1.093**	**1.294**	**<.0001**
	Osteoarthritis	32393	2041	**1.17**	**1.037**	**1.321**	**0.0111**
	Soft tissue disorders	32393	1487	**1.181**	**1.03**	**1.355**	**0.0174**
**XIV**	**Diseases of the genitourinary system**	29061	2713	**1.201**	**1.084**	**1.33**	**0.0005**
**XV**	**Pregnancy complications: hypertension in pre**	24970	39	1.264	0.581	2.752	0.5546
**XVIII**	Digestive and abdominal symptoms	32393	565	1.236	0.993	1.538	0.0577
**XIX**	**Poisoning**	32393	115	0.527	0.275	1.01	0.0535
**XX**	**Self-harm**	27195	111	0.721	0.396	1.315	0.2859
		**Decrease in green space**					
	**Death**	39108	1285	1.033	0.902	1.183	0.6392
**IV**	**Endocrine diseases**	37991	2629	1.027	0.921	1.145	0.63
	Diabetes	38342	2151	1.023	0.907	1.154	0.7075
	Obesity	39108	148	0.802	0.49	1.314	0.3811
**V**	**Mental and behavioural disorders**	38639	606	0.885	0.695	1.126	0.3187
	Disorders due to substance abuse	39108	231	0.938	0.635	1.385	0.7459
	Psychotic disorders	38831	199	0.835	0.544	1.279	0.4067
	Mood disorders	38881	265	0.946	0.665	1.345	0.7576
**VI**	**Diseases of the nervous system**	37564	2052	0.941	0.829	1.069	0.3511
	Sleep disorders	39108	1038	**0.802**	**0.666**	**0.966**	**0.0199**
**IX**	**Diseases of the circulatory system**	36052	3235	0.958	0.867	1.059	0.4001
	Hypertension	35442	2051	1.09	0.966	1.23	0.1621
	Ischemic heart diseases	38570	1077	1.166	0.991	1.372	0.065
	Angina pectoris	38738	478	1.18	0.924	1.507	0.1838
	Heart failure	39032	222	0.907	0.613	1.341	0.6233
	Cerebrovascular diseases	38900	502	0.907	0.699	1.178	0.4635
	Stroke	38953	414	0.935	0.703	1.242	0.6421
	Intracerebral haemorrhage	39088	58	0.942	0.446	1.989	0.8748
**X**	**Diseases of the respiratory system**	36743	1954	**1.168**	**1.034**	**1.318**	**0.0121**
	Chronic obstructive bronchitis	39074	154	0.904	0.564	1.446	0.6726
**XI**	**Diseases of the digestive system**	35968	3553	0.994	0.905	1.093	0.9074
**XII**	**Diseases of the skin**	38749	351	0.926	0.679	1.263	0.626
**XIII**	**Diseases of the musculoskeletal system**	33731	5132	**0.903**	**0.833**	**0.979**	**0.0136**
	Osteoarthritis	39108	2485	0.935	0.834	1.049	0.2554
	Soft tissue disorders	39108	2019	0.891	0.782	1.015	0.0832
**XIV**	**Diseases of the genitourinary system**	34987	3462	0.975	0.886	1.073	0.5982
**XV**	**Pregnancy complications: hypertension in pre**	29603	32	1.068	0.437	2.607	0.8852
**XVIII**	Digestive and abdominal symptoms	39108	743	1.083	0.886	1.323	0.4369
**XIX**	**Poisoning**	39108	126	0.885	0.523	1.495	0.647
**XX**	**Self-harm**	31074	95	0.975	0.553	1.72	0.9302

eTable 17. Association of favourable change in neighbourhood characteristics and lifestyle factors in participants with unhealthy lifestyle and disadvantaged neighbourhoods at baseline (Part A) and association of unfavourable change in nneighbourhood characteristics and lifestyle factors in Participants with healthy lifestyle and advantaged neighbourhoods at baseline (Part B) in a population with stable employment.

Change in neighbourhood							Lifestyle change at follow-up (outcome)								
characteristic	N (total)	N(cases) (%)	OR (95% CI)	N (total)	N(cases) (%)	OR (95% CI)	N (total)	N(cases) (%)	OR (95% CI)	N (total)	N(cases) (%)	OR (95% CI)	N (total)	N(cases) (%)	OR (95% CI)
**A. Participants with unhealthy lifestyle factors at baseline**	**Quitting smoking in current smokers**			**Reduce drinking from heavy to moderate**			**Physical activity in physically inactive**			**>5% weight loss among overweighted or obese**			**Multiple risks reduce to no or single risk**		
Neighbourhood education															
Stable disadvantaged	7533	1728 (22.9)	1	8181	3033 (37.1)	1	8520	4207 (49.4)	1	19554	2838 (14.5)	1	7757	3018 (38.9)	1
From disadvantaged to advantaged	1787	532 (29.8)	1.30 (1.15-1.46)	2429	888 (36.6)	0.92 (0.83-1.02)	1973	1045 (53.0)	1.13 (1.02-1.25)	4640	601 (13.0)	1.13 (1.03-1.25)	1691	700 (41.4)	1.06 (0.95-1.18)
Neighbourhood income															
Stable disadvantaged	7655	1776 (23.2)	1	8965	3192 (35.6)	1	8022	3882 (48.4)	1	18410	2623 (14.3)	1	7708	2939 (38.1)	1
From disadvantaged to advantaged	1497	490 (32.7)	1.36 (1.20-1.54)	2010	817 (40.7)	1.12 (1.01-1.25)	1610	863 (53.6)	1.11 (1.00-1.25)	3550	461 (13.0)	1.14 (1.02-1.27)	1374	606 (44.1)	1.16 (1.03-1.30)
Neighbourhood unemployment															
Stable disadvantaged	6632	1519 (22.9)	1	7617	2687 (35.3)	1	7085	3421 (48.3)	1	16305	2279 (14.0)	1	6675	2556 (38.3)	1
From disadvantaged to advantaged	2325	692 (29.8)	1.29 (1.16-1.43)	3342	1258 (37.6)	1.05 (0.96-1.15)	2827	1531 (54.2)	1.19 (1.09-1.31)	6559	900 (13.7)	1.02 (0.94-1.11)	2399	1031 (43.0)	1.13 (1.03-1.24)
Neighbourhood green space															
Stable disadvantaged	7597	1837 (24.2)	1	10006	3519 (35.2)	1	8652	4287 (49.6)	1	19860	2809 (14.1)	1	7866	3028 (38.5)	1
From disadvantaged to advantaged	1902	535 (28.1)	1.11 (0.99-1.24)	2392	922 (38.6)	1.05 (0.96-1.15)	2016	1085 (53.8)	1.11 (1.00-1.23)	4640	665 (14.3)	0.99 (0.90-1.09)	1749	799 (45.7)	1.25 (1.12-1.39)
**B. Participants with healthy lifestyle factors at baseline**	**Smoking relapse in ex-smokers**			**Heavy drinking in moderate drinkers**			**Physical inactivity in physically active**			**>5% weight gain among non-obese**			**Multiple risks energe**		
Neighbourhood education															
Stable advantaged	7857	508 (6.5)	1	34532	2828 (8.2)	1	36323	4398 (12.1)	1	38096	9558 (25.1)	1	38453	2602 (6.8)	1
From advantaged to disadvantaged	2205	183 (8.3)	1.19 (1.00-1.43)	8852	816 (9.2)	1.08 (0.99-1.17)	9180	1324 (14.4)	1.20 (1.12-1.28)	9599	2702 (28.2)	1.09 (1.03-1.14)	9594	902 (9.4)	1.35 (1.24-1.46)
Neighbourhood income															
Stable advantaged	8252	504 (6.1)	1	36180	2811 (7.8)	1	36858	4628 (12.6)	1	38586	9468 (24.5)	1	39474	2748 (7.0)	1
From advantaged to disadvantaged	1761	183 (10.4)	1.66 (1.39-2.00)	7028	650 (9.3)	1.14 (1.04-1.25)	7342	1056 (14.4)	1.16 (1.07-1.24)	7649	2141 (28.0)	1.13 (1.07-1.20)	7589	674 (8.9)	1.24 (1.13-1.35)
Neighbourhood unemployment															
Stable advantaged	6993	432 (6.2)	1	30365	2500 (8.2)	1	31348	3953 (12.6)	1	32843	8419 (25.6)	1	33329	2363 (7.1)	1
From advantaged to disadvantaged	3102	261 (8.4)	1.34 (1.15-1.55)	12621	1100 (8.7)	1.03 (0.96-1.11)	12900	1762 (13.7)	1.07 (1.01-1.14)	13528	3625 (26.8)	0.95 (0.89-1.01)	13600	1117 (8.2)	1.12 (1.04-1.21)
Neighbourhood green space															
Stable advantaged	9007	598 (6.6)	1	38143	2898 (7.6)	1	38031	5187 (13.6)	1	40048	10195 (25.5)	1	40742	3025 (7.4)	1
From advantaged to disadvantaged	2196	201 (9.2)	1.27 (1.07-1.51)	8869	763 (8.6)	1.08 (1.00-1.18)	9152	1287 (14.1)	1.08 (1.01-1.15)	9659	2644 (27.4)	1.04 (0.98-1.09)	9628	850 (8.8)	1.21 (1.11-1.31)

*Odds ratios (OR) are from logistic regression models with generalized estimating equations (GEE) estimation and adjustment for age, sex, education, data cycle and cohort.

eTable 18. Association of favourable change in neighbourhood characteristics and lifestyle factors in participants with unhealthy lifestyle and disadvantaged neighbourhoods at baseline (Part A) and association of unfavourable change in nneighbourhood characteristics and lifestyle factors in Participants with healthy lifestyle and advantaged neighbourhoods at baseline (Part B) in a population living in urban areas.

Change in neighbourhood	Lifestyle change at follow-up (outcome)														
characteristic	N (total)	N(cases) (%)	OR (95% CI)	N (total)	N(cases) (%)	OR (95% CI)	N (total)	N(cases) (%)	OR (95% CI)	N (total)	N(cases) (%)	OR (95% CI)	N (total)	N(cases) (%)	OR (95% CI)
**A. Participants with unhealthy lifestyle factors at baseline**	**Quitting smoking in current smokers**			**Reduce drinking from heavy to moderate**			**Physical activity in physically inactive**			**>5% weight decrease among overweighted or o**			**Multiple risks reduce to no or single risk**		
Neighbourhood education															
Stable disadvantaged	7939	1830 (23.1)	1	8729	3192 (36.6)	1	9868	4674 (47.4)	1	21488	3290 (15.3)	1	8707	3356 (38.5)	1
From disadvantaged to advantaged	1866	540 (28.9)	1.25 (1.11-1.40)	2633	968 (36.8)	0.95 (0.86-1.04)	2199	1146 (52.1)	1.13 (1.03-1.24)	4979	648 (13.0)	1.19 (1.08-1.30)	1862	772 (41.5)	1.08 (0.98-1.20)
Neighbourhood income															
Stable disadvantaged	8867	2100 (23.7)	1	10705	3766 (35.2)	1	10399	4878 (46.9)	1	22737	3453 (15.2)	1	9542	3600 (37.7)	1
From disadvantaged to advantaged	1492	486 (32.6)	1.36 (1.20-1.53)	2128	874 (41.1)	1.14 (1.03-1.26)	1709	898 (52.6)	1.10 (0.99-1.22)	3608	470 (13.0)	1.18 (1.07-1.32)	1428	626 (43.8)	1.17 (1.05-1.32)
Neighbourhood unemployment															
Stable disadvantaged	7622	1760 (23.1)	1	8956	3149 (35.2)	1	9069	4262 (47.0)	1	19990	2961 (14.8)	1	8164	3092 (37.9)	1
From disadvantaged to advantaged	2378	717 (30.2)	1.33 (1.20-1.48)	3560	1337 (37.6)	1.04 (0.96-1.14)	3149	1629 (51.7)	1.14 (1.05-1.24)	6983	1006 (14.4)	1.02 (0.94-1.10)	2594	1102 (42.5)	1.14 (1.04-1.25)
Neighbourhood green space															
Stable disadvantaged	8970	2210 (24.6)	1	12138	4235 (34.9)	1	11368	5516 (48.5)	1	24823	3705 (14.9)	1	9916	3814 (38.5)	1
From disadvantaged to advantaged	1736	499 (28.7)	1.14 (1.01-1.28)	2279	858 (37.7)	1.02 (0.92-1.12)	1956	1018 (52.0)	1.08 (0.97-1.19)	4341	628 (14.5)	1.03 (0.94-1.13)	1672	736 (44.0)	1.18 (1.06-1.31)
**B. Participants with healthy lifestyle factors at baseline**	**Smoking relapse in ex-smokers**			**Heavy drinking in moderate drinkers**			**Physical inactivity in physically active**			**>5% weight increase among non-obese**			**Multiple risks appear**		
Neighbourhood education															
Stable advantaged	9097	552 (6.1)	1	39665	3152 (8.0)	1	41187	5324 (12.9)	1	43627	10521 (24.1)	1	44033	2988 (6.8)	1
From advantaged to disadvantaged	2298	184 (8.0)	1.18 (0.99-1.41)	9362	837 (8.9)	1.06 (0.98-1.15)	9539	1478 (15.5)	1.22 (1.14-1.30)	10113	2863 (28.3)	1.12 (1.06-1.17)	10088	953 (9.5)	1.33 (1.24-1.44)
Neighbourhood income															
Stable advantaged	8734	488 (5.6)	1	38610	2890 (7.5)	1	38729	5215 (13.5)	1	40913	9643 (23.6)	1	41953	2943 (7.0)	1
From advantaged to disadvantaged	1907	183 (9.6)	1.59 (1.32-1.91)	7603	676 (8.9)	1.12 (1.03-1.23)	7823	1185 (15.2)	1.14 (1.06-1.22)	8280	2269 (27.4)	1.12 (1.06-1.19)	8211	740 (89.0)	1.23 (1.13-1.34)
Neighbourhood unemployment															
Stable advantaged	7511	435 (5.8)	1	32520	2629 (8.1)	1	33216	4441 (13.4)	1	35129	8600 (24.5)	1	35646	2535 (7.1)	1
From advantaged to disadvantaged	3275	252 (7.7)	1.28 (1.08-1.50)	13506	1121 (8.3)	0.99 (0.92-1.07)	13568	1993 (14.7)	1.09 (1.03-1.16)	14438	3819 (26.5)	1.00 (0.93-1.06)	14486	1195 (8.3)	1.11 (1.04-1.20)
Neighbourhood green space															
Stable advantaged	8146	502 (6.2)	1	35159	2628 (7.5)	1	34704	5049 (14.6)	1	36824	8893 (24.2)	1	37540	2853 (7.6)	1
From advantaged to disadvantaged	2291	204 (8.9)	1.26 (1.05-1.50)	9202	780 (8.5)	1.05 (0.96-1.14)	9392	1386 (14.8)	1.06 (0.99-1.13)	9989	2721 (27.2)	1.05 (1.00-1.11)	9955	890 (8.9)	1.17 (1.08-1.26)

*Odds ratios (OR) are from logistic regression models with generalized estimating equations (GEE) estimation and adjustment for age, sex, education, data cycle and cohort.

SUPPLEMENTARY ANALYSES ON SKIN DISEASES IN RELATION TO NEIGHBOURHOOD UNEMPLOYMENT

According to analysis of detailed diagnoses, diseases related to hygiene (ICD-diagnoses L02 “Cutaneous abscess, furuncle and carbuncle”, L30 “Other dermatitis” and L72 “Follicular cysts of skin and subcutaneous tissue”) characterised cases of skin disease participants exposed to change in neighbourhood unemployment rates (**eTable 18**). The rate of new-onset cases of ICD-diagnosis L02 per 10,000 was 5.3 in the group with reducing neighbourhood unemployment compared to 18.4 in the group with stable high neighbourhood unemployment. The corresponding rates were 17.8 in the group of increasing neighbourhood unemployment versus 15.2 in the group of stable low neighbourhood unemployment. The four rates were 2.6, 6.0, 7.3, 5.2 per 10,000 for ICD-diagnosis L30 and 6.6, 12.9, 13.6 and 5.6 per 10,000 for L72. Infection is a common risk factor for diagnoses L02, L30 and L72.

eTable 19. Cases of skin disease among non-movers by neighbourhood change category

		Change in neighbourhood unemployment							
		From high to low		Stable high		From low to high		Stable low	
		Person-years	77012	Person-years	219594	Person-years	99355	Person-years	256495
ICD-10	Diseases of the skin	N	Incidence per 100,000 py	N	Incidence per 100,000 py	N	Incidence per 100,000 py	N	Incidence per 100,000 py
L01	Impetigo	0	0.00	1	0.46	0	0.00	0	0.00
L02	Cutaneous abscess, furuncle and carbuncle	4	5.19	40	18.22	17	17.11	28	10.92
L03	Cellulitis	6	7.79	16	7.29	11	11.07	19	7.41
L04	Acute lymphadenitis	2	2.60	6	2.73	1	1.01	5	1.95
L05	Pilonidal cyst	6	7.79	14	6.38	3	3.02	7	2.73
L08	Other local infections of skin and subcutaneous ti	0	0.00	6	2.73	0	0.00	4	1.56
L10	Pemphigus	0	0.00	0	0.00	1	1.01	0	0.00
L12	Pemphigoid	0	0.00	2	0.91	0	0.00	3	1.17
L13	Other bullous disorders	0	0.00	0	0.00	0	0.00	2	0.78
L20	Atopic dermatitis	3	3.90	4	1.82	1	1.01	5	1.95
L23	Allergic contact dermatitis	0	0.00	0	0.00	0	0.00	1	0.39
L26	Exfoliative dermatitis	1	1.30	0	0.00	0	0.00	0	0.00
L27	Dermatitis due to substances taken internally	3	3.90	7	3.19	2	2.01	0	0.00
L28	Lichen simplex chronicus and prurigo	1	1.30	1	0.46	0	0.00	1	0.39
L29	Pruritus	0	0.00	1	0.46	1	1.01	1	0.39
L30	Other dermatitis	2	2.60	13	5.92	7	7.05	13	5.07
L40	Psoriasis	1	1.30	18	8.20	8	8.05	21	8.19
L43	Lichen planus	1	1.30	2	0.91	3	3.02	3	1.17
L44	Other papulosquamous disorders	0	0.00	0	0.00	1	1.01	0	0.00
L50	Urticaria	4	5.19	16	7.29	2	2.01	8	3.12
L51	Erythema multiforme	3	3.90	1	0.46	0	0.00	2	0.78
L52	Erythema nodosum	0	0.00	2	0.91	1	1.01	2	0.78
L53	Other erythematous conditions	0	0.00	1	0.46	0	0.00	0	0.00
L57	Skin changes due to chronic exposure to nonionizin	1	1.30	6	2.73	1	1.01	3	1.17
L60	Nail disorders	1	1.30	4	1.82	1	1.01	8	3.12
L63	Alopecia areata	0	0.00	0	0.00	0	0.00	1	0.39
L64	Androgenic alopecia	0	0.00	0	0.00	0	0.00	2	0.78
L70	Acne	0	0.00	1	0.46	0	0.00	0	0.00
L71	Rosacea	0	0.00	0	0.00	1	1.01	1	0.39
L72	Follicular cysts of skin and subcutaneous tissue	5	6.49	28	12.75	13	13.08	14	5.46
L73	Other follicular disorders	0	0.00	4	1.82	2	2.01	2	0.78
L75	Apocrine sweat disorders	0	0.00	1	0.46	0	0.00	0	0.00
L81	Other disorders of pigmentation	1	1.30	3	1.37	0	0.00	0	0.00
L82	Seborrhoeic keratosis	0	0.00	1	0.46	1	1.01	0	0.00
L84	Corns and callosities	1	1.30	2	0.91	2	2.01	6	2.34
L88	Pyoderma gangrenosum	0	0.00	0	0.00	2	2.01	0	0.00
L89	Decubitus ulcer	1	1.30	3	1.37	4	4.03	7	2.73
L90	Atrophic disorders of skin	2	2.60	3	1.37	4	4.03	14	5.46
L91	Hypertrophic disorders of skin	2	2.60	6	2.73	6	6.04	1	0.39
L92	Granulomatous disorders of skin and subcutaneous t	0	0.00	1	0.46	0	0.00	0	0.00
L93	Lupus erythematosus	0	0.00	1	0.46	0	0.00	1	0.39
L95	Vasculitis limited to skin, not elsewhere classifi	0	0.00	2	0.91	1	1.01	1	0.39
L97	Ulcer of lower limb, not elsewhere classified	0	0.00	5	2.28	0	0.00	4	1.56
L98	Other disorders of skin and subcutaneous tissue, n	4	5.19	5	2.28	2	2.01	5	1.95

STATISTICAL CODE

SAS version 9.4

```
**************************************************************************;
** Figures 2-5, eFigure 4, eTables 5,7,9,10,12-17 **;
**************************************************************************;
** disease=dg, exposure=aluealt, output file=resfile **;
** data sets: fh_taudit (diseases), hlot2 (persons) **;
**************************************************************************;
%macro coxit2 (dg,aluealt,resfile);
data tauti;
     set fh_taudit;
     IF dgnro=&dg;
data t1;
     merge hlot2(in=i) tauti;
     by tutknro;
     if i;
data t2;
     set t1;
     if slaalkupvm>. then status=1; else status=0;
     if kohortti=1 then seuraika=(min(slaalkupvm,kuolinpvm,mdy(12,31,2016))-
alkupvm+1)/365.25;
     if kohortti=1 and &dg=79 then
seuraika=(min(slaalkupvm,kuolinpvm,mdy(12,31,2018))-alkupvm+1)/365.25;
     if kohortti=2 then seuraika=(min(slaalkupvm,kuolinpvm,mdy(12,31,2012))-
alkupvm+1)/365.25;
     if &dg IN (4,5,6,7,8,9,10,11,12,15,18,20,21,22,24,25,26,
28,33,34,35,36,38,39,40,41,42,43,44,
        48,49,52,59,60,66,1,13,14,17,23,30,31,32,46,50,56,58,65,67) and
exslaalkupvm>. then extauti=1;
     if extauti=1 or (sex=1 and &dg IN (8,67,68,69,70)) or (sex=2 and &dg=9)
        then do; status=.; seuraika=.; end;
     if kohortti=2 and &dg IN (75,76,77,78) then do; status=.; seuraika=.; end;
proc phreg data=t2;
     class &aluealt;
     model seuraika*status(0) = sex age educ kohortti &aluealt / rl;
     ods output ParameterEstimates=pe CensoredSummary=cs;
     data pe; set pe(firstobs=5);
     data res; merge pe cs ; dgnro=&dg;
     keep parameter ClassVal0 dgnro Total Event HazardRatio HRLowerCL HRUpperCL
ProbChiSq;
     data res; merge res(in=i) lib.Tautiselitteet; by dgnro; if i;
run;
proc append base=&resfile data=res;
run;
%mend;

** all diseases **;
proc datasets lib=work memtype=data nolist; delete ases_results_all; quit;
%MACRO coxkaikki;
%DO I = 1 %TO 80;
    %coxit2(&I,ndvi5_rL2,ases_results_all);
%END;
%MEND coxkaikki;
%coxkaikki;
proc print data=ases_results_all;
     id dgnro;
     var selite parameter Total Event HazardRatio HRLowerCL HRUpperCL ProbChiSq;
run;

**************************************************************************;
** eTable 6 **;
```

```
*************************************************************************;
**  Cohort interactions  **;
*************************************************************************;
%macro coxinter (dg,aluealt,resfile);
data tauti;
     set fh_taudit;
     IF dgnro=&dg;
data t1;
     merge hlot2(in=i) tauti;
     by tutknro;
     if i;
data t2;
     set t1;
     if slaalkupvm>. then status=1; else status=0;
     if kohortti=1 then seuraika=(min(slaalkupvm,kuolinpvm,mdy(12,31,2016))-
alkupvm+1)/365.25;
     if kohortti=1 and &dg=79 then
seuraika=(min(slaalkupvm,kuolinpvm,mdy(12,31,2018))-alkupvm+1)/365.25;
     if kohortti=2 then seuraika=(min(slaalkupvm,kuolinpvm,mdy(12,31,2012))-
alkupvm+1)/365.25;
     if &dg IN (4,5,6,7,8,9,10,11,12,15,18,20,21,22,24,25,26,
28,33,34,35,36,38,39,40,41,42,43,44,
        48,49,52,59,60,66,1,13,14,17,23,30,31,32,46,50,56,58,65,67) and
exslaalkupvm>. then extauti=1;
     if extauti=1 or (sex=1 and &dg IN (8,67,68,69,70)) or (sex=2 and &dg=9)
        then do; status=.; seuraika=.; end;
     if kohortti=2 and &dg IN (75,76,77,78) then do; status=.; seuraika=.; end;
proc phreg data=t2;
     model seuraika*status(0) = sex age educ kohortti &aluealt kohortti*&aluealt
/ rl;
     ods output ParameterEstimates=pe CensoredSummary=cs;
      data pe; set pe(firstobs=6);
     data res; merge cs pe; dgnro=&dg;
     keep dgnro parameter Total Event ProbChiSq;
     data res; merge res(in=i) lib.Tautiselitteet; by dgnro; if i;
proc append base=&resfile data=res;
run;
%mend;

** all diseases **;
proc datasets lib=work memtype=data nolist; delete ases_results_all; quit;
%MACRO coxkaikki;
%DO I = 1 %TO 80;
    %coxinter(&I,aluestovuodet5_rL2,ases_results_all);
%END;
%MEND coxkaikki;
%coxkaikki;
proc print data=ases_results_all;
     id dgnro;
     var selite Total Event Parameter ProbChiSq;
run;

*************************************************************************;
** eTable 8 **;
*************************************************************************;
** Movers vs. non-movers: prevalent diseases **;
*************************************************************************;
%macro logreg (dg,aluealt,resfile);
data tauti;
     set fh_taudit;
     IF dgnro=&dg;
data t1;
     merge hlot2(in=i) tauti;
     by tutknro;
```

```
     if i;
data t2;
     set t1;
     if exslaalkupvm>. then extauti=1; else extauti=0;
     if (sex=1 and &dg IN (8,67,68,69,70)) or (sex=2 and &dg=9) then extauti=.;
     if kohortti=2 and &dg IN (75,76,77,78) then extauti=.;
proc logistic data=t2 descending;
     class &aluealt(ref='1') / param=ref;
     model extauti = &aluealt sex age educ kohortti;
     ods output ParameterEstimates=pe OddsRatios=or ResponseProfile=rp;
     data pe; set pe; if Variable='eimuuttaja5'; data or; set or; if
Effect='eimuuttaja5 0 vs 1';
     data rp1; set rp; if Outcome=1; rename count=Event;
     data rp0; set rp; if Outcome=0;
     data res; merge pe or rp1 rp0; dgnro=&dg; Total=count+event;
     keep Variable dgnro Total Event OddsRatioEst LowerCL UpperCL ProbChiSq;
     data res; merge res(in=i) lib.Tautiselitteet; by dgnro; if i;
proc append base=&resfile data=res;
run;
%mend;

** all diseases **;
proc datasets lib=work memtype=data nolist; delete ases_results_all; quit;
%MACRO coxkaikki;
%DO I = 1 %TO 80;
    %logreg(&I,eimuuttaja5,ases_results_all);
%END;
%MEND coxkaikki;
%coxkaikki;
proc print data=ases_results_all;
     id dgnro;
     var selite Variable Total Event OddsRatioEst LowerCL UpperCL ProbChiSq;
run;

*************************************************************************;
** eTable 2, eFigure 3 **;
*************************************************************************;
**  Testing the proportional hazard assumption in Cox models  **;
*************************************************************************;
%macro coxph (dg,aluealt,resfile);
data tauti;
     set fh_taudit;
     IF dgnro=&dg;
data t1;
     merge hlot2(in=i) tauti;
     by tutknro;
     if i;
data t2;
     set t1;
     if slaalkupvm>. then status=1; else status=0;
     if kohortti=1 then seuraika=(min(slaalkupvm,kuolinpvm,mdy(12,31,2016))-
alkupvm+1)/365.25;
     if kohortti=1 and &dg=79 then
seuraika=(min(slaalkupvm,kuolinpvm,mdy(12,31,2018))-alkupvm+1)/365.25;
     if kohortti=2 then seuraika=(min(slaalkupvm,kuolinpvm,mdy(12,31,2012))-
alkupvm+1)/365.25;
     if &dg IN (4,5,6,7,8,9,10,11,12,15,18,20,21,22,24,25,26,
28,33,34,35,36,38,39,40,41,42,43,44,
        48,49,52,59,60,66,1,13,14,17,23,30,31,32,46,50,56,58,65,67) and
exslaalkupvm>. then extauti=1;
     if extauti=1 or (sex=1 and &dg IN (8,67,68,69,70)) or (sex=2 and &dg=9)
        then do; status=.; seuraika=.; end;
     if kohortti=2 and &dg IN (75,76,77,78) then do; status=.; seuraika=.; end;
proc phreg data=t2;
```

```
      model seuraika*status(0) = sex age educ kohortti &aluealt alttime / rl;
      alttime=&aluealt*log(seuraika);
      proportionality_test: test alttime;
      ods output CensoredSummary=cs TestStmts=ph;
      data res; merge cs ph; dgnro=&dg;
      keep dgnro Total Event label WaldChiSq DF ProbChiSq;
      data res; merge res(in=i) lib.Tautiselitteet; by dgnro; if i;
proc append base=&resfile data=res;
run;
%mend;

** all diseases **;
proc datasets lib=work memtype=data nolist; delete ases_results_all; quit;
%MACRO coxkaikki;
%DO I = 1 %TO 80;
    %coxph(&I,aluestovuodet5_rL2,ases_results_all);
%END;
%MEND coxkaikki;
%coxkaikki;
proc print data=ases_results_all;
      id dgnro;
      var selite Total Event label WaldChiSq DF ProbChiSq;
run;

** log-log plot **;
proc lifetest data=t2 notable plots=(logsurv, lls);
time seuraika*status(0);
strata aluestovuodet5_rL2;
run;

*************************************************************************;
** Table 2-3 **;
*************************************************************************;
** A. Participants with healthy lifestyle factors at baseline **;
** Smoking relapse in ex-smokers **;
proc freq data=p2;
where ndvi0_rL2=0 and smoke1=1;
      tables ndvi5_rL2*smoking2/ nopercent nocol;
run;
proc genmod data=p2;
where ndvi0_rL2=0 and smoke1=1;
      class tutknro educ1 ndvi5_rL2;
      model smoking2(ref='0') = kohortti trial sex age1 educ1 ndvi5_rL2 /
dist=bin type3;
      repeated subject=tutknro / type=un;
      estimate 'From advantaged to disadvantaged' ndvi5_rL2 -1 1 / exp;
run;
** B. Participants with unhealthy lifestyle factors at baseline **;
** Quitting smoking in current smokers **;
proc freq data=p2;
      where ndvi0_rL2=1 and smoking1=1;
      tables ndvi5_rL2*smoking2/ nopercent nocol;
run;
proc genmod data=p2;
where ndvi0_rL2=1 and smoking1=1;
      class tutknro educ1 ndvi5_rL2;
      model smoking2(ref='1') = kohortti trial sex age1 educ1 ndvi5_rL2 /
dist=bin type3;
      repeated subject=tutknro / type=un;
      estimate 'From disadvantaged to advantaged' ndvi5_rL2 1 -1 / exp;
run;

*************************************************************************;
** Appendix 2 **;
```

```
***********************************************************************;
** Continuous exposure **;
***********************************************************************;
%macro coxitC (dg,aluealt,resfile);
data tauti;
     set fh_taudit;
     IF dgnro=&dg;
data t1;
     merge hlot2(in=i) tauti;
     by tutknro;
     if i;
data t2;
     set t1;
     if slaalkupvm>. then status=1; else status=0;
     if kohortti=1 then seuraika=(min(slaalkupvm,kuolinpvm,mdy(12,31,2016))-
alkupvm+1)/365.25;
     if kohortti=1 and &dg=79 then
seuraika=(min(slaalkupvm,kuolinpvm,mdy(12,31,2018))-alkupvm+1)/365.25;
     if kohortti=2 then seuraika=(min(slaalkupvm,kuolinpvm,mdy(12,31,2012))-
alkupvm+1)/365.25;
     if &dg IN (4,5,6,7,8,9,10,11,12,15,18,20,21,22,24,25,26,
28,33,34,35,36,38,39,40,41,42,43,44,
        48,49,52,59,60,66,1,13,14,17,23,30,31,32,46,50,56,58,65,67) and
exslaalkupvm>. then extauti=1;
     if extauti=1 or (sex=1 and &dg IN (8,67,68,69,70)) or (sex=2 and &dg=9)
        then do; status=.; seuraika=.; end;
     if kohortti=2 and &dg IN (75,76,77,78) then do; status=.; seuraika=.; end;
proc phreg data=t2; *where eimuuttaja5=1;
     model seuraika*status(0) = sex age educ kohortti &aluealt / rl;
     ods output ParameterEstimates=pe CensoredSummary=cs;
     data pe; set pe(firstobs=5);
     data res; merge pe cs ; dgnro=&dg;
     keep parameter dgnro Total Event HazardRatio HRLowerCL HRUpperCL ProbChiSq;
     data res; merge res(in=i) lib.Tautiselitteet; by dgnro; if i;
proc append base=&resfile data=res;
run;
%mend;

** all diseases **;
proc datasets lib=work memtype=data nolist; delete ases_results_all; quit;
%MACRO coxkaikki;
%DO I = 1 %TO 80;
    %coxitC(&I,stalueovuodet5,ases_results_all);
%END;
%MEND coxkaikki;
%coxkaikki;
proc print data=ases_results_all;
     id dgnro;
     var selite parameter Total Event HazardRatio HRLowerCL HRUpperCL ProbChiSq;
run;
```

REFERENCES

1. Korkeila K, Suominen S, Ahvenainen J, et al. Non-response and related factors in a nation-wide health survey. European journal of epidemiology 2001;17:991-9.
2. Piepoli MF, Hoes AW, Agewall S, et al. 2016 European Guidelines on cardiovascular disease prevention in clinical practice: The Sixth Joint Task Force of the European Society of Cardiology and Other Societies on Cardiovascular Disease Prevention in Clinical Practice (constituted by representatives of 10 societies and by invited experts)Developed with the special contribution of the European Association for Cardiovascular Prevention & Rehabilitation (EACPR). European heart journal 2016;37:2315-81.
3. Goff DC, Jr., Lloyd-Jones DM, Bennett G, et al. 2013 ACC/AHA guideline on the assessment of cardiovascular risk: a report of the American College of Cardiology/American Heart Association Task Force on Practice Guidelines. Journal of the American College of Cardiology 2014;63:2935-59.
4. Environmental_Protection_Agency. What is Open Space/Green Space?2018.
5. Gorelick N, Hancher M, Dixon M, Ilyushchenko S, Thau D, Moore R. Google Earth Engine: Planetary-scale geospatial analysis for everyone. Remote Sensing of Environment 2017;202:18-27.
6. Su JG, Dadvand P, Nieuwenhuijsen MJ, Bartoll X, Jerrett M. Associations of green space metrics with health and behavior outcomes at different buffer sizes and remote sensing sensor resolutions. Environ Int 2019;126:162-70.
7. Ren Z, Pu R, Zheng H, Zhang D, He X. Spatiotemporal analyses of urban vegetation structural attributes using multitemporal Landsat TM data and field measurements. Annals of Forest Science 2017;74.
8. Gascon M, Cirach M, Martínez D, et al. Normalized difference vegetation index (NDVI) as a marker of surrounding greenness in epidemiological studies: The case of Barcelona city. Urban Forestry & Urban Greening 2016;19:88-94.
9. Crouse DL, Pinault L, Balram A, et al. Urban greenness and mortality in Canada's largest cities: a national cohort study. Lancet Planet Health 2017;1:e289-e97.
10. Engemann K, Pedersen CB, Arge L, Tsirogiannis C, Mortensen PB, Svenning JC. Residential green space in childhood is associated with lower risk of psychiatric disorders from adolescence into adulthood. Proceedings of the National Academy of Sciences of the United States of America 2019;116:5188-93.
11. Kivimaki M, Nyberg ST, Batty GD, et al. Job strain as a risk factor for coronary heart disease: a collaborative meta-analysis of individual participant data. Lancet 2012;380:1491-7.
12. Department_of_Health. Sensible drinking: Report of an inter-departmental working group. . London1995.
13. Kujala UM, Kaprio J, Sarna S, Koskenvuo M. Relationship of leisure-time physical activity and mortality: the Finnish twin cohort. Jama 1998;279:440-4.
14. WHO. International Statistical Classification of Diseases and Related Health Problems 10th Revision. Geneva: World Health Organization; 2010.
15. Kivimaki M, Batty GD, Pentti J, et al. Association between socioeconomic status and the development of mental and physical health conditions in adulthood: a multi-cohort study. Lancet Public Health 2020;5:e140-e9.
16. Noble WS. How does multiple testing correction work? Nat Biotechnol 2009;27:1135-7.
17. Rothman KJ. No adjustments are needed for multiple comparisons. Epidemiology 1990;1:43-6.
18. Amrhein V, Greenland S, McShane B. Scientists rise up against statistical significance. Nature 2019;567:305-7.
